# General electrochemical Minisci alkylation of *N*-heteroarenes with alkyl halides†

**DOI:** 10.1039/d2sc01799g

**Published:** 2022-05-06

**Authors:** Roberto del Río-Rodríguez, Lorena Fragoso-Jarillo, Alberto F. Garrido-Castro, M. Carmen Maestro, Jose A. Fernández-Salas, José Alemán

**Affiliations:** Organic Chemistry Department, Universidad Autónoma de Madrid Módulo 2 28049 Madrid Spain jose.aleman@uam.es j.fernandez@uam.es; Institute for Advanced Research in Chemical Sciences (IAdChem), Universidad Autónoma de Madrid Madrid Spain; Center for Innovation in Advanced Chemistry (ORFEO-CINQA), Universidad Autónoma de Madrid Spain

## Abstract

Herein, we report, a general, facile and environmentally friendly Minisci-type alkylation of *N*-heteroarenes under simple and straightforward electrochemical conditions using widely available alkyl halides as radical precursors. Primary, secondary and tertiary alkyl radicals have been shown to be efficiently generated and coupled with a large variety of *N*-heteroarenes. The method presents a very high functional group tolerance, including various heterocyclic-based natural products, which highlights the robustness of the methodology. This applicability has been further proved in the synthesis of various interesting biologically valuable building blocks. In addition, we have proposed a mechanism based on different proofs and pieces of electrochemical evidence.

## Abstract 2

Herein, we report, a general, facile and environmentally friendly Minisci-type alkylation of *N*-heteroarenes under simple and straightforward electrochemical conditions using widely available alkyl halides as radical precursors.

## Graphical abstract

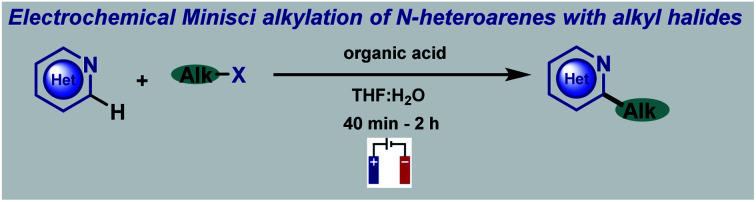

## Introduction

**Fig. 1:**
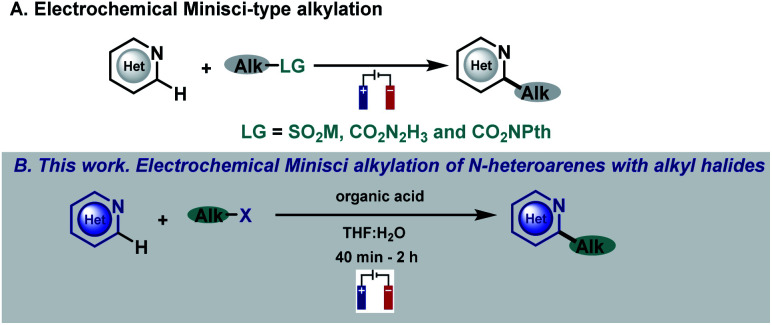
Previous work. (A) Electrochemical Minisci-type alkylation. (B) This work. Electrochemical Minisci alkylation of *N*-heteroarenes with alkyl halides.

*N*-heterocycles have attracted the attention of organic chemists over the years as they are versatile intermediates in organic synthesis and prevalent structures in natural and synthetic products with a wide spectrum of biological properties.^1,2^ Indeed, more than 85% of all biologically active compounds are heterocycles, highlighting their importance in organic and medicinal chemistry.^1^ Due to their relevance, the development of straightforward functionalizations of heterocyclic structures has been a focal point in organic chemistry research, with special emphasis on strategies capable of replacing C–H bonds with new and exciting functionalities in a single, direct and selective operation.^3,4^ In this context, the venerable Minisci reaction stands as a powerful and appealing synthetic tool for the direct and rapid modification of heteroaromatic units. Based on their innate reactivity, addition of a nucleophilic carbon radical species followed by a H atom loss provides the desired C–H functionalized derivatives, a reaction pathway which can afford a quick and efficient increase in molecular complexity.^4–6^ As a consequence of the importance of this challenge, a plethora of research groups have focused their efforts on developing new Minisci-type alkylation methodologies.^4–6^ Spurred by the resurgence in the field of photocatalysis, several reports have surfaced based on visible-light-mediated Minisci-type alkylations using a variety of radical precursors including, for example, peroxides,^7^ alcohols,^8^ boronic acids,^9,10^ alkyltrifluoroborates,^11^ carboxylic acids,^12–15^ redox-active esters (RAEs),^16–20^ secondary alkanes^21–27^ and alkyl halides.^28–33^ However, these strategies involve the use of expensive metal-based photocatalysts, an excess of additives (silanes, oxidants, surfactants, *etc.*), an inert atmosphere and long reaction times that hamper their broad applicability. Among the multiple strategies based on the single-electron activation of organic substrates, electrochemistry is quickly becoming one of the most popular avenues to access radical intermediates. The use of simple electrons as reagents through the application of an electrical current offers the possibility to promote redox events and carry out reactions under mild conditions with enhanced atom economy.^34–38^ In this context, electrochemical Minisci-type processes have recently begun to attract considerable attention employing different kinds of alkyl radical precursors (Fig. 1A).^39–42^

In this context, alkyl halides are of particular interest when looking at their radical precursor potential since they are readily available and inexpensive. Despite the great interest in including alkyl halides in the Minisci reaction electrochemical portfolio, this objective has not been accomplished. In fact, the electroreduction of radical precursors to engage in the formation of new C–C bonds is now being pursued and studied by various research groups^39–41^^,^^43–47^ as they allow the development of new methodologies with high generality and functional group tolerance when compared with less indulgent oxidative reaction conditions but have never been applied in Minisci-type alkylation processes. In this sense, reduction of halides under electrochemical conditions is still very underdeveloped probably due to the high potentials required for their direct reduction which has hampered its general applicability because of the very low functional group tolerance associated with those extreme potentials.^47–49^ Thus, we envisioned that the development of a facile and operationally simple Minisci-alkylation *via* electroreduction of readily available alkyl halides would be highly appealing, which may also provide new opportunities using this straightforward approach to generate carbon-centered radicals.

Herein, we describe a Minisci-type alkylation using alkyl halides under simple, mild and easy-to-handle electrochemical conditions with a high functional group tolerance (Fig. 1B). The electrochemically generated aliphatic radicals efficiently engage with nitrogen-based heteroarenes in the presence of a Brønsted acid acting as the sole promoter.

## Results

### Optimization of the model reaction

We began our investigations by studying the reaction of 4-methylquinoline (1a) as the model substrate with cyclohexyl iodide (2a) as a radical precursor (Table 1). By using diphenyl phosphate (PA), NH_4_PF_6_ as electrolyte at 10 mA constant current for 120 min in an undivided cell, the alkylated quinoline derivative 3a was isolated in excellent yield (92%, entry 1). Operationally, the setup uses a simple commercial potentiostat, without any of the ordinary precautions required in radical chemistry, such as meticulous exclusion of O_2_ or water, since the reaction takes place in a mixture of THF : H_2_O under air. For the development of a synthetically useful electrochemical Minisci-type alkylation, various parameters such as the additive or promoter, solvent, electrolyte, electrode material and electrochemical parameters have been studied and are summarized in Table 1 (see the ESI† for details). When the reaction was stopped after 42 min (2.4 F mol^−1^), 73% conversion of the desired alkylated heteroarene was obtained (entry 2). In the presence of other acids such as trifluoroacetic acid (TFA) or *para*-toluenesulfonic acid (*p*TsOH) the reaction took place less efficiently and lower reactivities were observed (entries 4 and 5), which demonstrates the special capability of PA to carry out the activation of quinoline derivatives.^17^ Different solvent systems commonly used under electrochemical reaction conditions (entries 6–9, see the ESI† for details) were then evaluated, showing that H_2_O as a co-solvent was necessary to competently perform the alkylation. Modification of the electrolyte showed that the use of a Brønsted acid-based electrolyte had a considerable effect on the reaction as it might also be involved in the activation of the heterocycle (entries 10 and 11). The electrode material selection had a great impact on the reaction as well. Thus, Zn or carbon-based electrodes (RVC) cathodes did not promote the reaction (entries 12 and 13). Finally, the reaction did not take place in the absence of electrical current (entry 14).

**Table 1:** Optimization of the electrochemical Minisci alkylation of 4-methylquinoline (1a)^a^

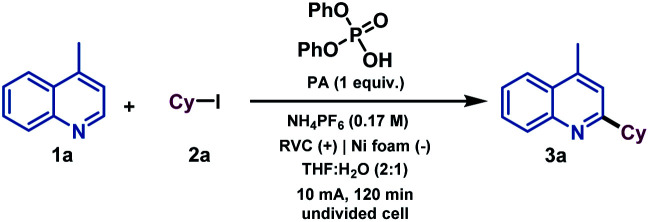		
Entry	Deviation from optimized conditions	Conversion^b^ (%)
1	No deviation	>98% (92%)^c^
2	42 min (2.4 F mol^−1^) instead of 120 min	73%
3^d^	No PA	24%
4^d^	TFA instead of PA	38%
5^d^	*p*TsOH instead of PA	43%
6^d^	THF instead of THF : H_2_O	17%
7^d^	DMF instead of THF : H_2_O	65%
8^d^	DMF : H_2_O instead of THF : H_2_O	41%
9^d^	MeOH : H_2_O instead of THF : H_2_O	23%
10^d^	TBAPF_6_ instead of NH_4_PF_6_	30%
11^d^	NH_4_BF_4_ instead of NH_4_PF_6_	65%
12^d^	RVC (+)|Zn (−) instead of RVC (+)|Ni (−)	n.r
13^d^	RVC (+)|RVC (−) instead of RVC (+)|Ni (−)	n.r
14	No current	n.r

a Reaction conditions: 1a (0.1 mmol) and 2a (0.5 mmol) at constant current (10 mA) and 7.4 F mol^−1^, electrolyte (0.5 mmol), THF : H_2_O (2 : 1, 3 mL), r.t, in air.

b Conversions were determined by ^1^H NMR.

c Isolated yield in brackets.

d Reaction performed at 2.4 F mol^−1.^

### Substrate scope

Once the reaction conditions had been optimized, a variety of alkyl iodides were tested under the electrochemical reaction conditions in an undivided cell with a readily available RVC anode and a nickel foam cathode. The initial aim of this study was to evaluate the generality of the system using 4-methylquinoline as the model substrate (Table 2). This exploration demonstrated that secondary alkyl radical precursors efficiently performed the alkylation in good yields (3a–3f). Notably, secondary alkyl iodides bearing heteroatoms were very well tolerated (3c, 3d and 3f). A sterically encumbered tertiary alkyl radical also led to the desired alkylated quinoline with a very good yield (3g). Furthermore, primary alkyl radicals were competently generated under the electrochemical conditions presented and furnished the desired 2-substituted quinolines in good yields (3h and 3i). We then explored the heteroaromatic radical acceptor. First, differently substituted quinolines were subjected to the optimized reaction conditions. Thus, methoxy- and bromide-bearing 4-methyl-substituted quinolines provided the desired alkylated heterocyclic systems (3j, 3k and 3l). Notably, aromatic bromides were perfectly compatible with the electrochemical reaction conditions, which should be highlighted as the presence of bromides was very often circumvented in the substrate scope evaluation of previous Minisci-type alkylation studies using alkyl halides,^28,30–33^ probably to avoid other reduction byproducts. We then evaluated the substitution at the 4-position of the quinoline. In addition to a phenyl substituent (3m), elusive moieties in previous Minisci alkylation protocols such as bromide (3o), ketone (3n), ester (3p) and amide (3q)^28,30–33^ were very well tolerated, giving rise to the alkylated quinolines with good yields. Additionally, we envisioned that 2-methylquinolines could lead to the desired 4-alkylated products as well (3r–t). When unsubstituted quinoline was subjected to the optimized reaction conditions, a 1 : 1 mixture of the 2- and 4-alkylated products was obtained (3u) as was observed for other Minisci reactions.^5^ Consequently, various *N*-heteroarenes were tested, such as isoquinoline (3v),^50^ phenantroline (3w), phenanthridine (3x), benzothiazole (3y) and benzimidazole (3z), which were selectively alkylated at the most electrophilic position in good yields. Moreover, in order to extend the applicability of the method, other recognizable quinolines were also studied under these electrochemical conditions. Thus, late-stage alkylations of complex natural products decorated with various functionalities such as quinoxyfen (fungicide, 3aa), quinine (3ab), cinchonidine (3ac) and (*S*)-(+)-Camptothecin (antitumor activity, 3ad) provided the corresponding C2 or C4 alkylation products in an efficient fashion. Notably, the model reaction of lepidine (1a) proceeded efficiently starting from 1 mmol (upscaling 10 times) and led to the desired alkylated quinoline derivative 3a in good yield (75%, see ESI†). It should be noted, that when 2 equiv. of the radical precursor (Cy-I, 2a) were employed, the reaction still showed a reasonable performance (3a, 75%).

**Table 2:** Substrate scope of the electrochemical Minisci alkylation^a^

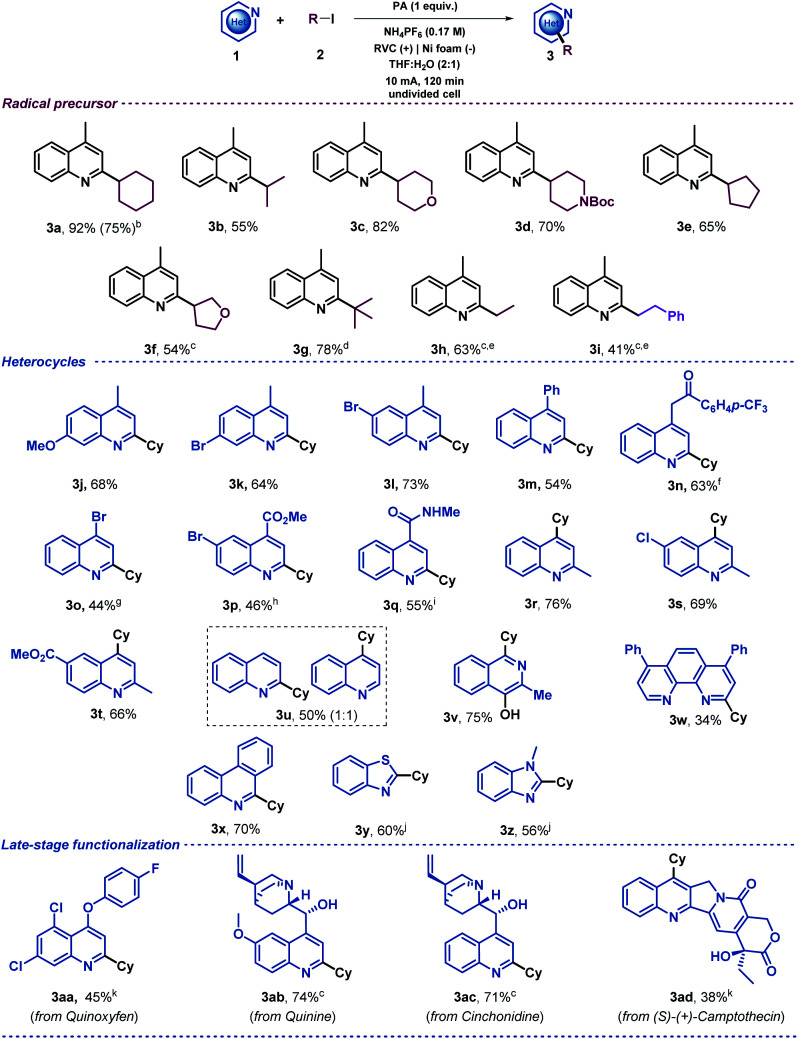

a Reaction conditions: 1 (0.1 mmol), 2 (0.5 mmol), PA (0.1 mmol) and NH_4_PF_6_ (0.5 mmol), THF : H_2_O (2 : 1, 3 mL), r.t, in air, undivided cell (RVC anode and Ni foam cathode) at constant current (10 mA) for 120 min. Isolated yields.

b Reaction performed with 1 mmol of 1a or under standard conditions using 0.2 mmol (2 equiv.) of Cyl (2a).

c 240 min.

d Zn cathode instead of Ni foam.

e 1 (0.05 mmol), PA (0.05 mmol), NH_4_PF_6_ (0.25 mmol).

f Constant current (7.5 mA) for 240 min.

g Constant current (5 mA) for 60 min.

h Constant current (5 mA) for 90 min.

i Constant current (15 mA) for 60 min.

j 480 min.

k Constant current (5 mA) for 120 min.

With the idea to expand the applicability of the method, we identified acridines as potential substrates for their selective and straightforward alkylation at C9 by using the presented electrochemical methodology.^51^ Acridine derivatives constitute a class of compounds with a broad spectrum of biological activity and are of great interest to the organic and medicinal chemistry fields.^52,53^ Therefore, due to the lack of straightforward approaches to accomplish the direct functionalization of acridines in the literature, the synthetic modification of this prized heterocyclic core could be particularly appealing. Gratifyingly, under slightly modified reaction conditions (see the ESI† for details), acridine (1v) was found to be a suitable alkyl radical acceptor. Thus, the *tert*-butyl radical was added to the C-9 position of acridine, giving rise to the corresponding dihydroacridine derivative in good yields (Table 3, 4a).

**Table 3:** Substrate scope of the electrochemical acridine alkylation^a^

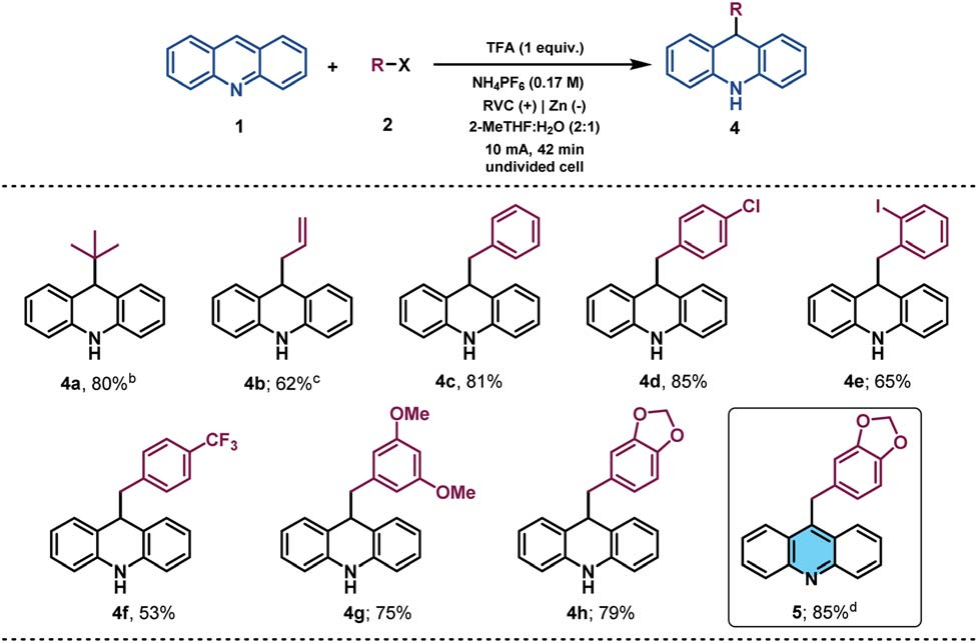

a Reaction conditions: 1 (0.1 mmol), 2 (0.5 mmol), NH_4_PF_6_ (0.5 mmol), TFA (0.1 mmol), 2-MeTHF : H_2_O (2 : 1, 3 mL), r.t, in air, undivided cell (RVC anode and Ni foam cathode) at constant current (10 mA) for 42 min. Isolated yields.

b THF instead of 2-MeTHF.

c 1 (0.1 mmol) and 2 (1.0 mmol), NH_4_PF_6_ (1.0 mmol), TFA (0.2 mmol), THF : H_2_O (2 : 1, 3 mL), r.t, in air, undivided cell (RVC anode and Ni foam cathode) at constant current (10 mA) for 42 min.

d From 4h: MnO_2_ (10 equiv.), THF (0.06 M), r.t., 16 hours.

**Scheme 1:**
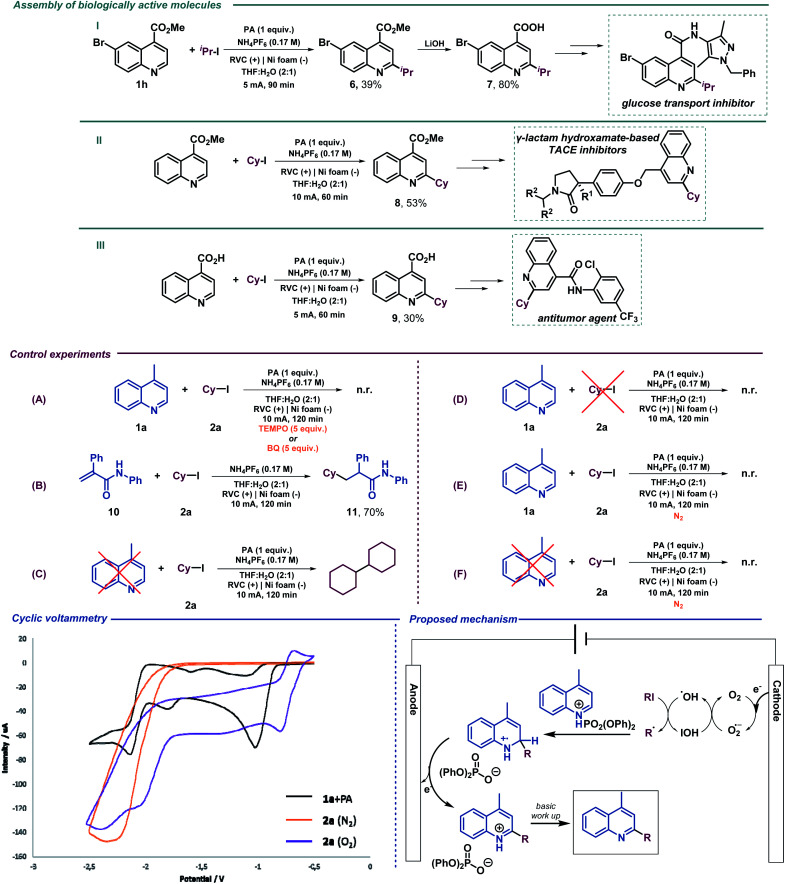
Assembly of biologically active molecules using the electrochemical Minisci alkylation. Control experiments, cyclic voltammetry and the proposed reaction mechanism for the electrochemical Minisci alkylation.

We next examined the allylation and benzylation of acridine, which would give access to products whose synthesis, to the best of our knowledge, has never been accomplished by a Minisci-type alkylation protocol.^54^ Therefore, allyl bromide was efficiently reduced under the electrochemical reaction conditions and led to the desired allylated product 4b in good yield. Different benzyl bromides with varying electronic natures (4c–h) were tolerated using 2-MeTHF (included in the “green” solvent list) as an environmentally friendly solvent. Notably, synthetically versatile halides such as chloride (4d) and even iodides (4e) were compatible to the electrochemical reaction setup, leading to benzylated dihydroacridine derivatives in good yields. Furthermore, the system was easily rearomatized under mild oxidative conditions to give the corresponding C9-functionalized acridine (5) in excellent yield. As shown before, interesting and versatile carboxylic acid derivatives allocated at the 4-position of quinoline were remarkably compatible under the electrochemical reaction conditions (Table 2). Encouraged by these results and in an attempt to expand the applicability of the method, we targeted the formal synthesis of various interesting drug molecules. Thus, the *iso*-propyl installation at the 2 position of ester-bearing quinoline 1h using our optimized electrochemical protocol led to the desired alkylated quinoline 6 with excellent selectivity. Simple hydrolysis of the ester moiety gave rise to carboxylic acid derivative 7, which has been used as a template for the construction of a more complex amide-substituted quinoline featured as a glucose transport inhibitor^55^ (Scheme 1, I). In addition, alkylated product 8, achieved directly following the electrochemical procedure, gave direct access to an intermediate in the synthesis of a tumor necrosis factor-α-converting enzyme (TACE) inhibitor^56^ (Scheme 1, II). To our delight, this electrochemical system could also provide the corresponding alkylated quinoline 9 in the presence of a free carboxylic acid in a synthetically useful yield, which is itself an intermediate in the synthesis of an antitumor agent^57^ (Scheme 1, III) or used as a template for the construction of anti-tuberculosis agents.^58^

### Mechanistic studies

We then performed a series of preliminary control experiments in order to gain an insight into the reaction mechanism. As shown in Scheme 2, in the presence of a radical scavenger such as TEMPO (2,2,6,6-tetramethylpiperidinooxy), the reaction was inhibited as only starting materials were observed untouched (Scheme 1(A)). In addition, when an acrylamide derivative (10) was used as a radical acceptor^41,47^ instead of lepidine, the corresponding Giese-type product was obtained in good yield (11) (Scheme 1(B)). These results strongly indicate that radical species are involved in the reaction pathway. When the reaction under the standard conditions was carried out in the absence of lepidine (1a), the alkyl halide was completely consumed, and only the homocoupling product, resulting from the cyclohexyl radical dimerization, was observed as it was previously observed when studying the scope of the reaction (Scheme 1(C)). However, when performed in the absence of 2a, lepidine activated with PA did not suffer any change or reduction process (Scheme 1(D)), which shows how the optimized electrochemical reaction conditions are unable to reach the required reduction potential (with and without PA, see the ESI†) for such a transformation (see cyclic voltammetry, Scheme 1). Therefore, PA is only enhancing the reactivity of the C=N bond.^17^ This may show a reductive pathway for the aliphatic halide, which might be generating carbon-centered radical species involved in the C–C bond forming event to yield the alkylated products. In addition, we tested the reaction under inert atmosphere conditions in order to evaluate if oxygen may be involved. In fact, under such conditions the reaction did not take place and only the starting materials were observed (Scheme 1(E)). Moreover, as expected based on the high reduction potential shown by halide 2a (see cyclic voltammetry, Scheme 1), when a control experiment under inert conditions in the absence of lepidine was carried out, no homocoupling product was observed (Scheme 1(F)). Therefore, oxygen should be involved in the generation of the initial reactive radical species. Moreover, as shown in Scheme 1A, in the presence of a superoxide scavenger such as benzoquinone (BQ), the reaction was inhibited and the radical precursor was observed untouched.

Based on the above-mentioned findings and supported by the literature,^40,47^ we propose the mechanism shown in Scheme 1. We suggest that aerobic oxygen is responsible for the initiation of the process. Upon reduction, shown to be feasible with the lowest reduction potential of the reaction components (see cyclic voltammetry, Scheme 1), the superoxide anion is formed and protonated to generate highly reactive peroxy radical species. These intermediates could be responsible for the generation of the carbon-centered radical *via* halogen atom abstraction of the alkyl halide. Following alkyl radical generation, addition to the activated (protonated) *N*-heteroarene would result in the formation of a new carbon–carbon bond. Finally, the putative radical intermediate would then undergo rearomatization to deliver the final Minisci-type adduct.

## Conclusions

In conclusion, we have described a general and facile Minisci-type alkylation of *N*-heteroarenes under simple and straightforward electrochemical conditions using available alkyl halides as radical precursors. The reaction system has demonstrated its robustness and generality as primary, secondary and tertiary alkyl radical precursors and a variety of heterocycles have shown their compatibility within this electrochemical system. In addition, various heterocyclic-based natural products have been successfully integrated into the reaction scope. Moreover, as a consequence of the high functional group tolerance of the method, we have shown how the electrochemical Minisci-type alkylation methodology can be efficiently used to achieve biologically valuable compounds.

## Data availability

All experimental data, and detailed experimental procedures are available in the ESI.†

## Author contributions

R. R. R. carried out the optimization and scope of the reaction. L. F. J. and A. F. G. C. participated in the optimization. M. C. M., J. A. F. S. and J. A. conceived the project and prepared the manuscript, which was edited by all other authors.

## Conflicts of interest

There are no conflicts to declare.

## Supplementary Material

SC-013-D2SC01799G-s001

## References

[cit1] Heravi M. M., Zadsirjan V. (2020). RSC Adv..

[cit2] Eicher T. , Hauptmann S. and Speicher A., The Chemistry of Heterocycles, Wiley-VCH Verlag GmbH & Co, Weinheim, 2nd edn, 2003

[cit3] Fujiwara Y. and Baran P. S., Radical-Based Late Stage C–H Functionalization of Heteroaromatics in Drug Discovery, in New Horizons of Process Chemistry, ed. K. Tomioka, T. Shioiri and H. Sajiki, Springer Nature, Singapore, 2017, pp. 103–120

[cit4] Duncton M. A. J. (2011). Med. Chem. Commun..

[cit5] Proctor R. S. J., Phipps R. J. (2019). Angew. Chem., Int. Ed..

[cit6] Sun A. C., McAtee R. C., McClain E. J., Stephenson C. R. J. (2019). Synthesis.

[cit7] DiRocco D. A., Dykstra K., Krska S., Vachal P., Conway D. V., Tudge M. (2014). Angew. Chem., Int. Ed..

[cit8] Jin J., MacMillan D. W. C. (2015). Nature.

[cit9] Li G.-X., Morales-Rivera C. A., Wang Y., Gao F., He G., Liu P., Chen G. (2016). Chem. Sci..

[cit10] Dong J., Yue F., Song H., Liu Y., Wang Q. (2020). Chem. Commun..

[cit11] Matsui J. K., Primer D. N., Molander G. A. (2017). Chem. Sci..

[cit12] Garza- Sanchez R. A., Tlahuext-Aca A., Tavakoli G., Glorius F. (2017). ACS Catal..

[cit13] Wang J., Li G.-X., He G., Chen G. (2018). Asian J. Org. Chem..

[cit14] Tian W.-F., Hu C.-H., He K.-H., He X.-Y., Li Y. (2019). Org. Lett..

[cit15] Papaioannou N., Fray M. J., Rennhack A., Sanderson T. J., Stokes J. E. (2020). J. Org. Chem..

[cit16] Parida S. K., Mandal T., Das S., Hota S. K., Sarkar S. D., Murarka S. (2021). ACS Catal..

[cit17] Jin S., Geng X., Li Y., Zheng K. (2021). Eur. J. Org. Chem..

[cit18] Hagui W., Cordier M., Boixel J., Soulé J.-F. (2021). Chem. Commun..

[cit19] Dong J., Wang X., Song H., Liu Y., Wang Q. (2020). Adv. Synth. Catal..

[cit20] Qin P.-T., Sun J., Wang F., Wang J.-Y., Wang H., Zhoua M.-D. (2020). Adv. Synth. Catal..

[cit21] Capaldo L., Quadri L. L., Merli D., Ravelli D. (2021). Chem. Commun..

[cit22] Proctor R. S. J., Chuentragool P., Colgan A. C., Phipps R. J. (2021). J. Am. Chem. Soc..

[cit23] Deng Z., Li G.-X., He G., Chen G. (2019). J. Org. Chem..

[cit24] Wang Z., Ji X., Han T., Deng G.-J., Huang H. (2019). Adv. Synth. Catal..

[cit25] Tian H., Yang H., Tian C., An G., Li G. (2020). Org. Lett..

[cit26] Rammal F., Gao D., Boujnah S., Gaumont A. −C., Hussein A. A., Lakhdar S. (2020). Org. Lett..

[cit27] Li G., Gao Y., Jia C., Wang S., Yan B., Fang Y., Yang S. (2020). Org. Lett..

[cit28] Santos M. S., Cybularczyk-Cecotka M., König B., Giedyk M. (2020). Chem. - Eur. J..

[cit29] Jung S., Shin S., Park S., Hong S. (2020). J. Am. Chem. Soc..

[cit30] Dong J., Lyu X., Wang Z., Wang X., Song H., Liua Y., Wang Q. (2019). Chem. Sci..

[cit31] Perkins J. J., Schubert J. W., Streckfuss E. C., Balsells J., ElMarrouni A. (2020). Eur. J. Org. Chem..

[cit32] Nuhant P., Oderinde M. S., Genovino J., Juneau A., Gagné Y., Allais C., Chinigo G. M., Choi C., Sach N. W., Bernier L., Fobian Y. M., Bundesmann M. W., Khunte B., Frenette M., Fadeyi O. O. (2017). Angew. Chem., Int. Ed..

[cit33] Bissonnette N. B., Boyd M. J., May G. D., Giroux S., Nuhant P. (2018). J. Org. Chem..

[cit34] Möhle S., Zirbes M., Rodrigo E., Gieshoff T., Wiebe A., Waldvogel S. R. (2018). Angew. Chem., Int. Ed..

[cit35] Meyer T. H., Choi I., Tian C., Ackermann L. (2020). Chem.

[cit36] Yan M., Kawamata Y., Baran P. S. (2017). Chem. Rev..

[cit37] Horn E. J., Rosen B. R., Baran P. S. (2016). ACS Cent. Sci..

[cit38] Pollok D., Waldvogel S. R. (2020). Chem. Sci..

[cit39] O'Brien A. G., Maruyama A., Inokuma Y., Fujita M., Baran P. S. (2014). Angew. Chem., Int. Ed..

[cit40] Liu Y., Xue L., Shi B., Bu F., Wang D., Lu L., Shi R., Lei A. (2019). Chem. Commun..

[cit41] Niu K., Song L., Hao Y., Liu Y., Wang Q. (2020). Chem. Commun..

[cit42] Gao Y., Wu Z., Yu L., Wang Y., Pan Y. (2020). Angew. Chem., Int. Ed..

[cit43] Yang D.-T., Zhu M., Schiffer Z. J., Williams K., Song X., Liu X., Manthiram K. (2019). ACS Catal..

[cit44] Huang J.-M., Wang X.-X., Dong Y. (2011). Angew. Chem., Int. Ed..

[cit45] Huang B., Guo L., Xia W. (2021). Green Chem..

[cit46] Lian F., Xu K., Meng W., Zhang H., Tana Z., Zeng C. (2019). Chem. Commun..

[cit47] Li D., Ma T.-K., Scott R. J., Wilden J. D. (2020). Chem. Sci..

[cit48] Paddon C. A., Bhatti F. L., Donohoe T. J., Compton R. G. (2007). J. Phys. Org. Chem..

[cit49] Minisci F., Vismara E., Fontana F. (1989). J. Org. Chem..

[cit50] Russell G. A., Rajaratnam R., Wang L., Shi B. Z., Kim B. H., Yao C. F. (1993). J. Am. Chem. Soc..

[cit51] For tert-butylation of acridines using tert-butyl mercury halides, please see ref. 50.

[cit52] Gensicka-Kowalewska M., Cholewinski G., Dzierzbicka K. (2017). RSC Adv..

[cit53] Gabriel I. (2020). Molecules.

[cit54] Dong J., Wang X., Wang Z., Song H., Liu Y., Wang Q. (2019). Org. Chem. Front..

[cit55] Heisler I. , Müller T., Siebeneicher H., Buchmann B., Cleve A. and Günther J., et al., WO2015091428, 2015

[cit56] Argade A., Bahekar R., Desai J., Thombare P., Shah K., Gite S. (2011). et al.. Med. Chem. Commun..

[cit57] Chen X., Sun W., Huang S., Zhang H., Lin G., Li H., Qiao J., Li L., Yang S. (2020). J. Med. Chem..

[cit58] Nayyar A., Malde A., Jain R., Coutinho E. (2006). Bioorg. Med. Chem..

