# Supplementary material for: General electrochemical Minisci alkylation of N-heteroarenes with alkyl halides
Source: Chem Sci. 2022 May 6;13(22):6512–8. doi: 10.1039/d2sc01799g (PMC9172443; doi:10.1039/d2sc01799g)
Supplement: SC-013-D2SC01799G-s001 [file SC-013-D2SC01799G-s001.pdf]

Electronic Supplementary Material (ESI) for Chemical Science.
This journal is © The Royal Society of Chemistry 2022

General Electrochemical Minisci Alkylation of *N*-Heteroarenes with Alkyl Halides

Roberto del Río-Rodríguez,[1] Lorena Fragoso-Jarillo,[1] Alberto F. Garrido-Castro,[1] Carmen Maestro-Rubio,[1] Jose A. Fernández-Salas,*[1,2] and José Alemán*[1,2,3]

[1]*Organic Chemistry Department, Módulo 2, Universidad Autónoma de Madrid, 28049 Madrid (Spain).*

[2]*Institute for Advanced Research in Chemical Sciences (IAdChem), Universidad Autónoma de Madrid, Madrid (Spain).*

[3]*Center for Innovation in Advanced Chemistry (ORFEO-CINQA), Universidad Autónoma de Madrid (Spain).*

* E-mail: j.fernandez@uam.es; jose.aleman@uam.es; web page: www.uam.es/jose.aleman

1. General methods and starting materials....3
2. Optimization tables....4
3. General procedure A: Alkylation of heteroaryl compounds....6
4. General procedure B: Alkylation, allylation and benzylation of acridine....18
5. General procedure C: Derivatizations....22
6. Nuclear magnetic resonance spectra....24
7. Cyclic Voltammetry....61
8. References....63

1. General methods and starting materials

Starting materials **1a-h**, **1k-1y**, **2a-p** as well as solvents for the reactions, were acquired from commercial sources (tetrahydrofuran was inhibitor free, water was tab water). Starting materials **1i**, **1j** and **10** were synthesized following a procedure described in the literature.[1] For thin layer chromatography (TLC), silica gel plates with fluorescence indicator 254 nm were used and compounds were visualized by irradiation with UV light and/or by treatment with a solution of potassium permanganate in water followed by heating. Flash column chromatography was performed using Geduran® Silica Gel 60 (0.040-0.063 nm). Cyclohexane, ethyl acetate, dichloromethane and methanol for flash chromatography were acquired from commercial sources and were used without previous purification. NMR spectra were acquired on a *Bruker Avance 300 MHz spectrometer*, running at 300 and 75 MHz for ^{1}H and ^{13}C, respectively. ^{19}F-NMR spectra were acquired on a *Bruker Avance 500 MHz spectrometer*, running at 471 MHz. Chemical shifts (δ) are reported in ppm relative to residual solvent signals ($CDCl_3$, 7.26 ppm for ^{1}H-NMR and 77.2 ppm for ^{13}C-NMR). ^{13}C-NMR was acquired on a broad band decoupled mode. The following abbreviations are used to describe peak patterns when appropriate: s (singlet), d (doublet), t (triplet), q (quartet), quint (quintet), m (multiplet), bs (broad singlet), tt (triplet of triplets), td (triplet of doublets). Electrospray ionization has been used for measuring the exact mass (indicated for each case): MS (ESI) (Electrospray ionization mass spectroscopy) was acquired with an *Agilent Technologies 6120 Quadrupole LC/MS*. In this technique, *MassWorks software ver. 4.0.0.0 (Cerno Bioscience*) was used for the formula identification. *MassWorks* is a MS calibration software which calibrates for isotope profile as well as for mass accuracy, allowing highly accurate comparisons between calibrated and theoretical spectra.[2]

2. Optimization tables

Table 1. Alkylation of 4-methylquinoline.

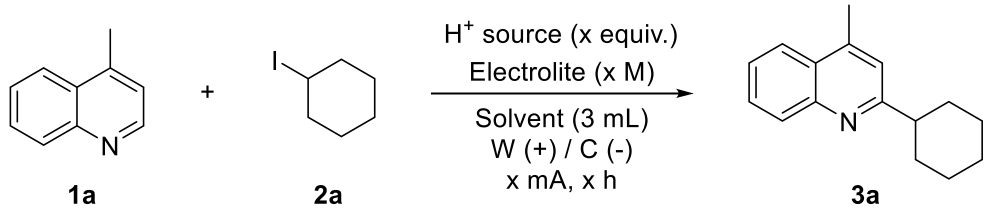

Entry[a]	*Solvent (3 mL)*	*Current (mA)*	*Time (min)*	*W (+) / C (-)*	*Conv. (%)*[b]
1	THF/H_2O (2:1)	-	42	RVC / Ni foam	-
2	THF/H_2O (2:1)	10	42	RVC / Ni foam	73
3[d]	THF/H_2O (2:1)	10	42	RVC / Ni foam	24
4[e]	THF/H_2O (2:1)	10	42	RVC / Ni foam	38
5[f]	THF/H_2O (2:1)	10	42	RVC / Ni foam	43
6	THF/H_2O (2:1)	30	42	RVC / Ni foam	56
7	THF/H_2O (2:1)	3	42	RVC / Ni foam	46
8	MeTHF / H_2O (2:1)	10	42	RVC / Ni foam	27
9	MeOH / H_2O (2:1)	10	42	RVC / Ni foam	23
10	DMF / H_2O (2:1)	10	42	RVC / Ni foam	41
11	THF	10	42	RVC / Ni foam	17
12	DMF	10	42	RVC / Ni foam	65
13[g]	THF/H_2O (2:1)	10	42	RVC / Ni foam	30
14[h]	THF/H_2O (2:1)	10	42	RVC / Ni foam	65
15	THF/H_2O (2:1)	10	42	RVC / Zn	-
16	THF/H_2O (2:1)	10	42	RVC / RVC	-
17	**THF/H_2O (2:1)**	**10**	**120**	**RVC / Ni foam**	**>98 (92)**[c]
18[i]	THF/H_2O (2:1)	10	120	RVC / Ni foam	88 (80)[c]
19[j]	THF/H_2O (2:1)	10	120	RVC / Ni foam	87 (75)[c]

[a]Standard reaction conditions: 0.1 mmol of **1a** and 0.5 mmol of **2a** in THF:H_2O (2:1, 3mL) with NH_4PF_6 (0.5 mmol) and diphenyl phosphate (0.1 mmol), under air atmosphere, were set at constant current for indicated time at room temperature. [b]Conversions were determined by ^{1}H-NMR. [c]Isolated yield in brackets. [d]No diphenyl phosphate. [e]TFA instead of diphenyl phosphate. [f]*p*TsOH instead of diphenyl phosphate. [g]$TBAPF_6$ instead of NH_4PF_6. [h]NH_4BF_4 instead of NH_4PF_6. [i]0.02 mmol of diphenyl phosphate. [j]0.02 mmol of diphenyl phosphate and 0.2 equiv. of **2a**.

Table 2. Alkylation, allylation and benzylation of acridine.

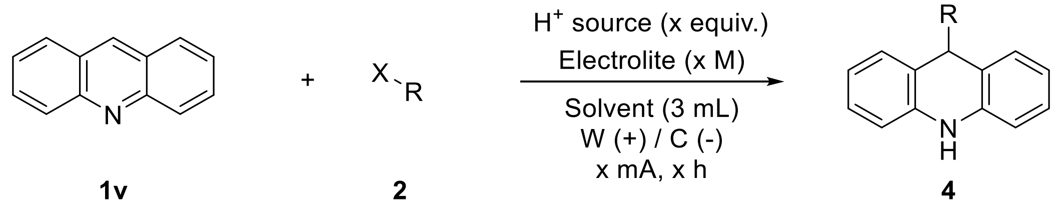

1v **2** **4**

Entry[a]	*X-R*	*Solvent (3 mL)*	*Current (mA)*	*Time (min)*	*W (+) / C (-)*	*Conv. (%)*[b]
1	**I-^{t}Bu**	**THF/H_2O (2:1)**	**10**	**42**	**RVC / Zn**	**>98 (80)[c]**
2	Br-Allyl	THF/H_2O (2:1)	10	42	RVC / Zn	-
3[d]	Br-Allyl	THF/H_2O (2:1)	10	42	RVC / Ni foam	70
4[d,e]	**Br-Allyl**	**THF/H_2O (2:1)**	**10**	**42**	**RVC / Ni foam**	**90 (62)[c]**
5[d,e]	Br-Allyl	DMF	10	42	RVC / Ni foam	-
6[d,e,f]	Br-Allyl	THF/H_2O (2:1)	10	42	RVC / Ni foam	-
7[d,e]	Br-Allyl	THF/H_2O (2:1)	3	42	RVC / Ni foam	-
8[d,e]	Br-Allyl	THF/H_2O (2:1)	15	42	RVC / Ni foam	55
9[d]	Br-Bn	THF/H_2O (2:1)	10	42	RVC / Ni foam	66
10[d]	Br-Bn	THF/H_2O (2:1)	10	42	RVC / Zn	100 (70)[c]
11[d]	**Br-Bn**	**MeTHF/H_2O (2:1)**	**10**	**42**	**RVC / Zn**	**100 (81)[c]**

[a]Standard reaction conditions: 0.1 mmol of **1v** and 0.5 mmol of **2a** in THF:H_2O (2:1, 3mL) with NH_4PF_6 (0.5 mmol) and diphenyl phosphate (0.1 mmol), under air atmosphere, were set at constant current for indicated time at room temperature. [b]Conversions were determined by ^{1}H-NMR. [c]Isolated yield in brackets. [d]TFA instead of diphenyl phosphate. [e]1 mmol of allyl bromide and NH_4PF_6. [f]$TBAPF_6$ instead of NH_4PF_6.

3. General procedure A: Alkylation of heteroaryl compounds

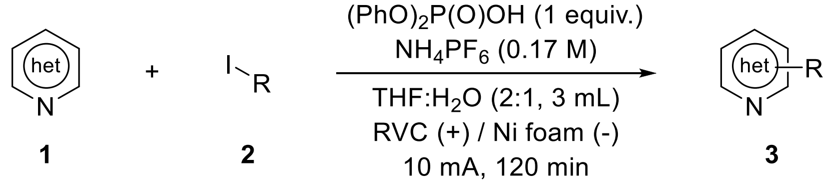

Diphenyl phosphate (25.0 mg, 1 equiv.), ammonium hexafluorophosphate (81.5 mg, 5 equiv.) and a magnetic stirrer were added to a 5 mL ElectraSyn vial. Reagents were dissolved in THF (2 mL) and to the stirred solution were added **1** (0.1 mmol) and **2** (5 equiv.), followed by water (1 mL). The vial was closed, reticulated vitreous carbon was used as working electrode and nickel foam as counter electrode, ElectraSyn 2.0 was set at constant current (10 mA) during 120 min. The crude mixture was then diluted with ethyl acetate, extracted with saturated aqueous solution of $NaHCO_3$ (2 x 5 mL), washed with brine (3 x 30 mL), dried over anhydrous $MgSO_4$, filtered and the solvent was evaporated under reduced pressure. The crude mixture was purified by flash column chromatography using silica gel and the eluent indicated in each case.

2-Cyclohexyl-4-methylquinoline (3a)

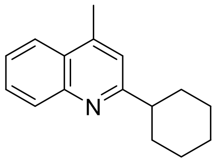

Following the general procedure A; 4-methylquinoline **1a** (13.2 µL, 0.1 mmol) and iodocyclohexane **2a** (64.7 µL, 0.5 mmol) gave product **3a** (92% yield) as a colorless oil. Eluent: cyclohexane: ethyl acetate from 100:0 to 80:20.

^{1}H-NMR: δ 8.05 (d, *J* = 8.2 Hz, 1H), 7.94 (d, *J* = 8.3 Hz, 1H), 7.70 – 7.61 (m, 1H), 7.52 – 7.45 (m, 1H), 7.17 (s, 1H), 2.88 (tt, *J* = 11.9, 3.4 Hz, 1H), 2.68 (s, 3H), 2.07 – 1.96 (m, 2H), 1.95 – 1.84 (m, 2H), 1.83 – 1.74 (m, 1H), 1.72 – 1.55 (m, 2H), 1.54 – 1.37 (m, 2H), 1.37 – 1.24 (m, 1H) ppm.
Spectra data are consistent with those reported in the literature.[3]

<u>The reaction was scaled up to 1.0 mmol.</u> Procedure A was followed using a 10 mL ElectraSyn vial as 6 mL of THF and 3 mL of water were used as solvents. The reaction was carried out at 10 mA for 16 hours. After workup and purification as described above, **3a** (167 mg, 75% yield) was obtained as a slightly yellow oil.

2-Isopropyl-4-methylquinoline (3b)

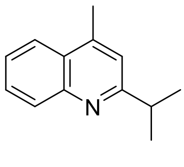

Following the general procedure A; 4-methylquinoline **1a** (13.2 µL, 0.1 mmol) and 2-iodopropane **2b** (49.1 µL, 0.5 mmol) gave product **3b** (55% yield) as a colorless oil. Eluent: cyclohexane: ethyl acetate from 100:0 to 80:20.

^{1}H-NMR: δ 8.08 (d, *J* = 8.4 Hz, 1H), 7.95 (d, *J* = 8.3 Hz, 1H), 7.72 – 7.63 (m, 1H), 7.54 – 7.46 (m, 1H), 7.18 (s, 1H), 3.32 – 3.15 (m, 1H), 2.69 (s, 3H), 1.39 (d, *J* = 7.0 Hz, 6H) ppm.

Spectra data are consistent with those reported in the literature. [3]

4-Methyl-2-(tetrahydro-2H-pyran-4-yl)quinoline (3c)

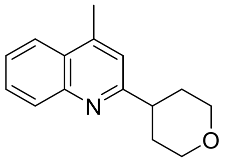

Following the general procedure A; 4-methylquinoline **1a** (13.2 µL, 0.1 mmol) and 4-iodotetrahydro-2H-pyran **2c** (106 mg, 0.5 mmol) gave product **3c** (82% yield) as a colorless oil. Eluent: cyclohexane: ethyl acetate from 100:0 to 80:20.

^{1}H-NMR: δ 8.06 (d, *J* = 8.4 Hz, 1H), 7.96 (d, *J* = 8.3 Hz, 1H), 7.72 – 7.64 (m, 1H), 7.56 – 7.47 (m, 1H), 7.18 (s, 1H), 4.13 (dd, *J* = 11.0, 2.9 Hz, 2H), 3.60 (td, *J* = 11.6, 2.5 Hz, 2H), 3.14 (tt, *J* = 11.7, 4.2 Hz, 1H), 2.70 (s, 3H), 2.11 – 1.86 (m, 4H) ppm.

Spectra data are consistent with those reported in the literature.[3]

***tert*-Butyl 4-(4-methylquinolin-2-yl)piperidine-1-carboxylate (3d)**

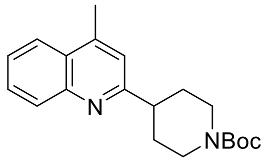

Following the general procedure A; 4-methylquinoline **1a** (13.2 µL, 0.1 mmol) and tert-butyl 4-iodopiperidine-1-carboxylate **2d** (155.5 mg, 0.5 mmol) gave product **3d** (70% yield) as a colorless oil. Eluent: cyclohexane: ethyl acetate from 100:0 to 80:20.

^{1}H-NMR: δ 8.03 (d, *J* = 8.5 Hz, 1H), 7.95 (d, *J* = 8.3 Hz, 1H), 7.68 (t, *J* = 7.6 Hz, 1H), 7.51 (t, *J* = 7.6 Hz, 1H), 7.14 (s, 1H), 4.38 – 4.19 (m, 2H), 3.01 (tt, *J* = 11.9, 3.9 Hz, 1H), 2.88 (t, *J* = 12.2 Hz, 2H), 2.69 (s, 3H), 2.02 – 1.92 (m, 2H), 1.91 – 1.75 (m, 2H), 1.49 (s, 9H) ppm.

Spectra data are consistent with those reported in the literature. [4]

2-Cyclopentyl-4-methylquinoline (3e)

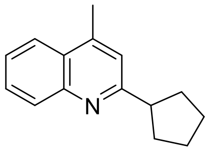

Following the general procedure A; 4-methylquinoline **1a** (13.2 µL, 0.1 mmol) and iodocyclopentane **2e** (57.8 µL, 0.5 mmol) gave product **3e** (65% yield) as a colorless oil. Eluent: cyclohexane: ethyl acetate from 100:0 to 80:20.

^{1}H-NMR: δ 8.05 (d, *J* = 8.4 Hz, 1H), 7.94 (d, *J* = 8.3 Hz, 1H), 7.70 – 7.62 (m, 1H), 7.53 – 7.45 (m, 1H), 7.18 (s, 1H), 3.42 – 3.26 (m, 1H), 2.68 (s, 3H), 2.24 – 2.09 (m, 2H), 1.94 – 1.80 (m, 4H), 1.80 – 1.68 (m, 2H) ppm.

Spectra data are consistent with those reported in the literature.[3]

4-Methyl-2-(tetrahydrofuran-3-yl)quinoline (3f)

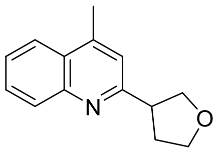

Following a slightly modified procedure A; 4-methylquinoline **1a** (13.2 µL, 0.1 mmol) and 3-iodotetrahydrofuran **2f** (44.0 µL, 0.5 mmol) gave product **3f** (54% yield) as a colorless oil when the reaction was carried out at 10 mA for 4 hours. Eluent: cyclohexane: ethyl acetate from 100:0 to 80:20.

^{1}H-NMR: δ 8.03 (d, *J* = 8.5 Hz, 1H), 7.96 (d, *J* = 8.2 Hz, 1H), 7.73 – 7.64 (m, 1H), 7.57 – 7.48 (m, 1H), 7.21 (s, 1H), 4.28 – 4.11 (m, 2H), 4.06 (dd, *J* = 8.6, 6.7 Hz, 1H), 3.95 (q, *J* = 7.9 Hz, 1H), 3.81 – 3.67 (m, 1H), 2.69 (s, 3H), 2.54 – 2.40 (m, 1H), 2.38 – 2.23 (m, 1H) ppm.
Spectra data are consistent with those reported in the literature.[3]

2-(*tert*-Butyl)-4-methylquinoline (3g)

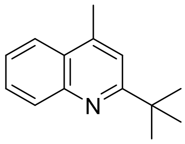

Following a slightly modified procedure A; 4-methylquinoline **1a** (13.2 µL, 0.1 mmol) and 2-iodo-2-methylpropane **2g** (59.6 µL, 0.5 mmol) gave product **3g** (78% yield) as a colorless oil, when Zn was used as counterelectrode. Eluent: cyclohexane: ethyl acetate from 100:0 to 80:20.

^{1}H-NMR: δ 8.07 (d, *J* = 8.3 Hz, 1H), 7.94 (d, *J* = 8.3 Hz, 1H), 7.71 – 7.62 (m, 1H), 7.54 – 7.45 (m, 1H), 7.36 (s, 1H), 2.69 (s, 3H), 1.46 (s, 9H) ppm.
Spectra data are consistent with those reported in the literature.[3]

2-Ethyl-4-methylquinoline (3h)

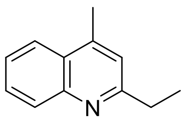

Following a slightly modified procedure A; 4-methylquinoline **1a** (6.6 µL, 0.05 mmol) and iodoetane **2h** (40.2 µL, 0.5 mmol) gave product **3h** (63% yield) as a colorless oil when the reaction was carried out at 10 mA for 4 hours in 2 mL of THF and 1 mL of H_2O with 1 equivalent of diphenyl phosphate and 5 equivalents of electrolyte. Eluent: cyclohexane: ethyl acetate from 100:0 to 80:20.

^{1}H-NMR: δ 8.11 (d, *J* = 8.4 Hz, 1H), 7.96 (d, *J* = 8.3 Hz, 1H), 7.73 – 7.64 (m, 1H), 7.56 – 7.47 (m, 1H), 7.18 (s, 1H), 2.99 (q, *J* = 7.6 Hz, 2H), 2.70 (s, 3H), 1.39 (t, *J* = 7.6 Hz, 3H) ppm.
Spectra data are consistent with those reported in the literature.[3]

4-Methyl-2-phenethylquinoline (3i)

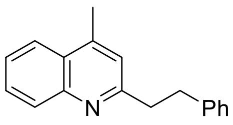

Following a slightly modified procedure A; 4-methylquinoline **1a** (6.6 µL, 0.05 mmol) and (2-iodoethyl)benzene **2i** (72.4 µL, 0.5 mmol) gave product **3i** (41% yield) as a colorless oil when the reaction was carried

out at 10 mA for 4 hours in 2 mL of THF and 1 mL of H_2O with 1 equivalent of diphenyl phosphate and 5 equivalents of electrolyte. Eluent: cyclohexane: ethyl acetate from 100:0 to 80:20.

^{1}H-NMR: δ 8.07 (d, *J* = 8.5 Hz, 1H), 7.97 (d, *J* = 8.2 Hz, 1H), 7.69 (t, *J* = 7.7 Hz, 1H), 7.52 (t, *J* = 7.6 Hz, 1H), 7.33 – 7.26 (m, 5H), 7.24 – 7.16 (m, 1H), 7.11 (s, 1H), 3.30 – 3.20 (m, 2H), 3.19 – 3.07 (m, 2H), 2.67 (s, 3H) ppm.
Spectra data are consistent with those reported in the literature. [5]

2-Cyclohexyl-7-methoxy-4-methylquinoline (3j)

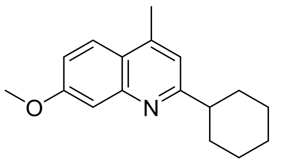

Following the general procedure A; 7-methoxy-4-methylquinoline **1b** (20.9 mg, 0.1 mmol) and iodocyclohexane **2a** (64.7 µL, 0.5 mmol) gave product **3j** (68% yield) as a colorless oil. Eluent: cyclohexane: ethyl acetate from 100:0 to 80:20.

^{1}H-NMR: δ 7.82 (d, *J* = 9.1 Hz, 1H), 7.39 (d, *J* = 2.6 Hz, 1H), 7.14 (dd, *J* = 9.1, 2.6 Hz, 1H), 7.03 (s, 1H), 3.94 (s, *J* = 4.8 Hz, 1H), 2.83 (tt, *J* = 11.9, 3.4 Hz, 1H), 2.64 (s, *J* = 0.6 Hz, 1H), 2.06 – 1.96 (m, 1H), 1.95 – 1.84 (m, 1H), 1.83 – 1.73 (m, 1H), 1.69 – 1.57 (m, 1H), 1.56 – 1.28 (m, 1H) ppm.

^{13}C-NMR: δ 167.12, 160.5, 149.6, 144.4, 124.9, 122.2, 118.5, 118.4, 107.8, 55.7, 47.9, 33.1 (2C), 26.8 (2C), 26.3, 19.0 ppm.

HRMS (ESI$^+$): calculated for $C_{17}H_{22}NO$ $[M-H]^+$: 256.1696; found: 256.1638.

7-Bromo-2-cyclohexyl-4-methylquinoline (3k)

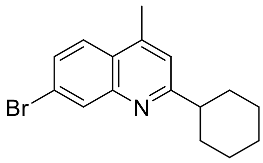

Following the general procedure A; 7-bromo-4-methylquinoline **1c** (22.2 mg, 0.1 mmol) and iodocyclohexane **2a** (64.7 µL, 0.5 mmol) gave product **3k** (64% yield) as a colorless oil. Eluent: cyclohexane: ethyl acetate from 100:0 to 80:20.

^{1}H-NMR: δ 8.23 (d, *J* = 2.0 Hz, 1H), 7.79 (d, *J* = 8.9 Hz, 1H), 7.56 (dd, *J* = 8.9, 2.0 Hz, 1H), 7.16 (d, *J* = 0.8 Hz, 1H), 2.84 (tt, *J* = 11.9, 3.4 Hz, 1H), 2.66 (d, *J* = 0.8 Hz, 3H), 2.04 – 1.94 (m, 2H), 1.94 – 1.84 (m, 2H), 1.84 – 1.74 (m, 1H), 1.71 – 1.54 (m, 3H), 1.54 – 1.35 (m, 2H) ppm.

^{13}C-NMR: δ 167.9, 148.7, 144.5, 132.0, 128.9, 125.9, 125.2, 123.2, 121.0, 47.6, 32.9 (2C), 26.7 (2C), 26.3, 18.9 ppm.

HRMS (ESI$^+$): calculated for $C_{16}H_{19}BrN$ $[M-H]^+$: 304.0695; found: 304.0639.

6-Bromo-2-cyclohexyl-4-methylquinoline (3l)

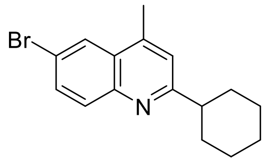

Following the general procedure A; 6-bromo-4-methylquinoline **1d** (22.2 mg, 0.1 mmol) and iodocyclohexane **2a** (64.7 µL, 0.5 mmol) gave product **3l** (73% yield) as a colorless oil. Eluent: cyclohexane: ethyl acetate from 100:0 to 90:10.

^{1}H-NMR: δ 8.08 (d, *J* = 2.2 Hz, 1H), 7.90 (d, *J* = 8.9 Hz, 1H), 7.72 (dd, *J* = 8.9, 2.2 Hz, 1H), 7.17 (s, 1H), 2.84 (tt, *J* = 11.8, 3.4 Hz, 1H), 2.64 (s, 3H), 2.05 – 1.95 (m, 2H), 1.95 – 1.84 (m, 2H), 1.84 – 1.73 (m, 2H), 1.69 – 1.51 (m, 3H), 1.50 – 1.30 (m, 2H) ppm.

^{13}C-NMR: δ 167.2, 146.5, 143.6, 132.5, 131.5, 128.5, 126.2, 121.3, 119.4, 47.7, 32.9 (2C), 26.7 (2C), 26.3, 19.0 ppm.

HRMS (ESI$^+$): calculated for $C_{16}H_{19}BrN$ $[M-H]^+$: 304.0695; found: 304.0670.

2-Cyclohexyl-4-phenylquinoline (3m)

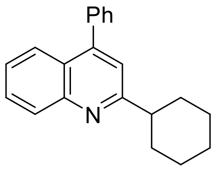

Following the general procedure A; 4-phenylquinoline **1e** (20.5 mg, 0.1 mmol) and iodocyclohexane **2a** (64.7 µL, 0.5 mmol) gave product **3m** (54% yield) as a colorless oil. Eluent: cyclohexane: ethyl acetate from 100:0 to 95:5.

^{1}H-NMR: δ 8.13 (d, *J* = 8.4 Hz, 1H), 7.86 (d, *J* = 8.4 Hz, 1H), 7.68 (ddd, *J* = 8.4, 6.9, 1.2 Hz, 1H), 7.55 – 7.47 (m, 5H), 7.43 (ddd, *J* = 8.4, 6.9, 1.2 Hz, 1H), 7.27 (s, 1H), 3.05 – 2.88 (m, 1H), 2.14 – 2.02 (m, 2H), 1.98 – 1.84 (m, 2H), 1.84 – 1.75 (m, 1H), 1.75 – 1.59 (m, 2H), 1.58 – 1.41 (m, 2H), 1.39 – 1.28 (m, 1H) ppm.

Spectra data are consistent with those reported in the literature.[3]

2-(2-Cyclohexylquinolin-4-yl)-1-(4-(trifluoromethyl)phenyl)ethan-1-one (3n)

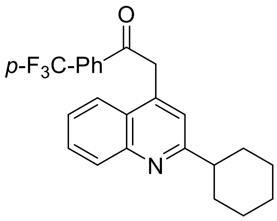

Following a slightly modified procedure A; 2-(quinolin-4-yl)-1-(4-(trifluoromethyl)phenyl)ethan-1-one **1f** (31.5 mg, 0.1 mmol) and iodocyclohexane **2a** (64.7 µL, 0.5 mmol) gave product **3n** (63% yield) as a yellow oil when it was carried out at 7.5 mA for 4 hours. Eluent: cyclohexane: ethyl acetate from 100:0 to 95:5 to 90:10.

^{1}H-NMR: δ 8.16 (d, *J* = 8.6 Hz, 2H), 8.10 (d, *J* = 8.4 Hz, 1H), 7.82 – 7.74 (m, 3H), 7.68 (ddd, *J* = 8.4, 6.9, 1.2 Hz, 1H), 7.48 (ddd, *J* = 8.4, 6.9, 1.2 Hz, 1H), 7.19 (s, 1H), 4.73 (s, 2H), 2.88 (tt, *J* = 11.7, 3.3 Hz, 1H), 2.07 – 1.95 (m, 2H), 1.94 – 1.83 (m, 2H), 1.83 – 1.73 (m, 1H), 1.69 – 1.53 (m, 2H), 1.53 – 1.24 (m, 3H) ppm.

^{13}C-NMR: δ 195.6, 166.6, 148.3, 140.4, 139.2, 135.08 (q, *J* = 32.8 Hz), 130.1, 129.5, 129.0 (2C), 126.4, 126.3, 126.1 (q, *J* = 3.7 Hz, 2C), 123.7 (q, *J* = 272.8 Hz), 123.3, 121.6, 47.6, 42.9, 32.9 (2C), 26.7 (2C), 26.2 ppm.

^{19}F-NMR: δ -63.2 ppm.

HRMS (ESI$^+$): calculated for $C_{24}H_{23}F_3NO$ $[M-H]^+$: 398.1726; found: 398.1787.

4-Bromo-2-cyclohexylquinoline (3o)

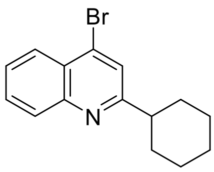

Following a slightly modified procedure A; 4-bromoquinoline **1g** (20.8 mg, 0.1 mmol) and iodocyclohexane **2a** (64.7 µL, 0.5 mmol) gave product **3o** (44% yield) as a colorless oil when it was carried out at 5 mA for 1 hour. Eluent: cyclohexane: ethyl acetate from 100:0 to 90:10.

^{1}H-NMR: δ 8.13 (d, *J* = 8.4 Hz, 1H), 8.03 (d, *J* = 8.4 Hz, 1H), 7.77 – 7.68 (m, 1H), 7.63 (s, 1H), 7.61 – 7.53 (m, 1H), 2.88 (tt, *J* = 11.8, 3.3 Hz, 1H), 2.09 – 1.98 (m, 2H), 1.97 – 1.85 (m, 2H), 1.84 – 1.74 (m, 1H), 1.70 – 1.54 (m, 2H), 1.54 – 1.40 (m, 2H), 1.40 – 1.24 (m, 1H) ppm.
Spectra data are consistent with those reported in the literature. [5]

Methyl 6-bromo-2-cyclohexylquinoline-4-carboxylate (3p)

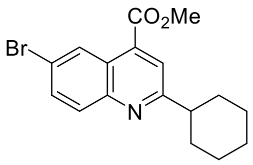

Following a slightly modified procedure A; ethyl 6-bromoquinoline-4-carboxylate **1h** (26.6 mg, 0.1 mmol) and iodocyclohexane **2a** (64.7 µL, 0.5 mmol) gave product **3p** (46% yield) as a colorless oil when the reaction was carried out at 5 mA for 90 minutes. Eluent: cyclohexane: ethyl acetate from 100:0 to 95:5.

^{1}H-NMR: δ 8.93 (d, *J* = 2.1 Hz, 1H), 7.95 (d, *J* = 9.0 Hz, 1H), 7.87 (s, 1H), 7.79 (dd, *J* = 9.0, 2.2 Hz, 1H), 4.04 (s, 3H), 2.93 (tt, *J* = 11.9, 3.4 Hz, 1H), 2.07 – 1.97 (m, 2H), 1.96 – 1.86 (m, 2H), 1.85 – 1.75 (m, 1H), 1.74 – 1.58 (m, 2H), 1.55 – 1.42 (m, 2H), 1.37 – 1.27 (m, 1H) ppm.

^{13}C-NMR: δ 166.9, 166.6, 147.6, 134.2, 133.2, 131.4, 128.0, 125.0, 122.3, 121.8, 53.0, 47.5, 32.7 (2C), 26.6 (2C), 26.2 ppm.

HRMS (ESI$^+$): calculated for $C_{17}H_{19}BrNO_2$ $[M-H]^+$: 348.0594; found: 348.0600.

2-Cyclohexyl-N-methylquinoline-4-carboxamide (3q)

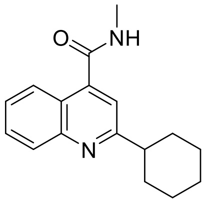

Following a slightly modified procedure A; *N*-methylquinoline-4-carboxamide **1j** (18.6 mg, 0.1 mmol) and iodocyclohexane **2a** (64.7 µL, 0.5 mmol) gave product **3q** (55% yield) as a colorless oil when the reaction was carried out at 15 mA for 1 hour. Eluent: cyclohexane: ethyl acetate from

100:0 to 80:20.

^{1}H-NMR: δ 8.10 (d, *J* = 8.3 Hz, 1H), 8.04 (d, *J* = 8.5 Hz, 1H), 7.74 – 7.64 (m, 1H), 7.55 – 7.45 (m, 1H), 7.34 (s, 1H), 6.25 (s, 1H), 3.07 (d, *J* = 4.7, 3H), 2.87 (t, *J* = 10.9 Hz, 1H), 2.03 – 1.73 (m, 6H), 1.68 – 1.21 (m, 4H) ppm.

^{13}C-NMR: δ 168.7, 166.5, 148.4, 142.6, 130.0, 129.5, 126.8, 125.1, 123.3, 117.3, 47.6, 32.9 (2C), 26.9, 26.6 (2C), 26.2 ppm.

HRMS (ESI$^+$): calculated for $C_{17}H_{21}N_2O$ $[M-H]^+$: 269.1648; found: 269.1605.

4-Cyclohexyl-2-methylquinoline (3r)

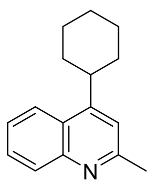

Following the general procedure A; 2-methylquinoline **1l** (13.1 µL, 0.1 mmol) and iodocyclohexane **2a** (64.7 µL, 0.5 mmol) gave product **3r** (76% yield) as a colorless oil. Eluent: cyclohexane: ethyl acetate from 100:0 to 80:20.

^{1}H-NMR: δ 8.09 – 7.98 (m, 2H), 7.70 – 7.58 (m, 1H), 7.57 – 7.42 (m, 1H), 7.16 (s, 1H), 3.39 – 3.19 (m, 1H), 2.72 (s, 3H), 2.09 – 1.79 (m, 5H), 1.64 – 1.44 (m, 3H), 1.44 – 1.23 (m, 2H) ppm.

Spectra data are consistent with those reported in the literature.[3]

6-Chloro-4-cyclohexyl-2-methylquinoline (3s)

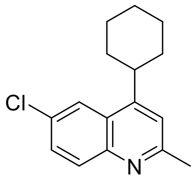

Following the general procedure A; 6-chloro-2-methylquinoline **1m** (17.8 mg, 0.1 mmol) and iodocyclohexane **2a** (64.7 µL, 0.5 mmol) gave product **3s** (69% yield) as a colorless oil. Eluent: cyclohexane: ethyl acetate from 100:0 to 90:10.

^{1}H-NMR: δ 8.00 – 7.93 (m, 2H), 7.58 (dd, *J* = 9.0, 2.3 Hz, 1H), 7.18 (s, 1H), 3.24 – 3.12 (m, 1H), 2.70 (s, 3H), 2.03 – 1.81 (m, 5H), 1.64 – 1.46 (m, 4H), 1.43 – 1.24 (m, 1H) ppm.

^{13}C-NMR: δ 159.4, 152.8, 146.7, 131.3, 131.3, 129.8, 126.1, 122.2, 119.4, 39.0, 33.7 (2C), 27.0 (2C), 26.4, 25.7 ppm.

HRMS (ESI$^+$): calculated for $C_{16}H_{19}ClN$ $[M-H]^+$: 260.1201; found: 260.1190.

Methyl 4-cyclohexyl-2-methylquinoline-6-carboxylate (3t)

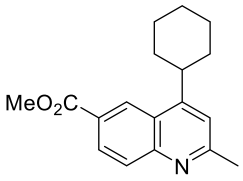

Following the general procedure A; ethyl 2-methylquinoline-6-carboxylate **1n** (20.1 mg, 0.1 mmol) and iodocyclohexane **2a** (64.7 µL, 0.5 mmol) gave product **3t** (66% yield) as a colorless oil. Eluent: cyclohexane: ethyl acetate from 100:0 to 90:10.

^{1}H-NMR: δ 8.80 (d, *J* = 1.6 Hz, 1H), 8.23 (dd, *J* = 8.8, 1.6 Hz, 1H), 8.04 (d, *J* = 8.8 Hz, 1H), 7.23 (s, 1H), 4.00 (s, 3H), 3.46 – 3.30 (m, 1H), 2.74 (s, 3H), 2.07 – 1.82 (m, 5H), 1.70 – 1.49 (m, 4H), 1.47 – 1.28 (m, 1H) ppm.

^{13}C-NMR: δ 167.3, 161.6, 155.1, 150.4, 130.0, 128.6, 127.0, 126.3, 124.6, 119.4, 52.6, 38.8, 34.0 (2C), 27.0 (2C), 26.4, 25.9 ppm.

HRMS (ESI$^+$): calculated for $C_{18}H_{22}NO_2$ $[M-H]^+$: 284.1645; found: 284.1637.

2-Cyclohexylquinoline (3u) and 4-Cyclohexylquinoline (3u')

Following a slightly modified procedure A; quinoline **1w** (11.8 µL, 0.1 mmol) and iodocyclohexane **2a** (64.7 µL, 0.5 mmol) gave product **3u** (26% yield) and **3u'** (24% yield) as a colorless oil when the reaction was carried out at 5 mA for 60 minutes. Eluent: cyclohexane: ethyl acetate from 100:0 to 80:20.

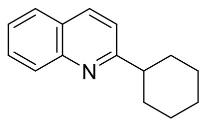

^{1}H-NMR: δ 8.13 – 7.99 (m, 2H), 7.82 – 7.73 (m, 1H), 7.73 – 7.58 (m, 1H), 7.47 (dd, *J* = 8.5, 6.9 Hz, 1H), 7.33 (d, *J* = 8.5 Hz, 1H), 3.01 – 2.84 (m, 1H), 2.11 – 1.97 (m, 2H), 1.97 – 1.85 (m, 2H), 1.82 – 1.63 (m, 1H), 1.57 – 1.45 (m, 2H), 1.38 – 1.10 (m, 2H), 0.97 – 0.80 (m, 1H) ppm.

Spectra data are consistent with those reported in the literature.[9]

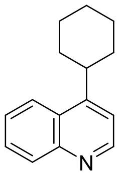

^{1}H-NMR: δ 8.85 (d, *J* = 4.7 Hz, 1H), 8.16 – 8.06 (m, 2H), 7.69 (ddd, *J* = 8.4, 6.8, 1.4 Hz, 1H), 7.56 (ddd, *J* = 8.4, 6.8, 1.4 Hz, 1H), 7.28 (d, *J* = 4.7 Hz, 1H), 3.42 – 3.28 (m, 1H), 2.08 – 1.81 (m, 6H), 0.99 – 0.79 (m, 4H) ppm.

Spectra data are consistent with those reported in the literature.[9]

1-Cyclohexyl-3-methylisoquinolin-4-ol (3v)

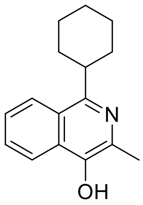

Following the general procedure A; 3-methylisoquinoline **1x** (14.3 mg, 0.1 mmol) and iodocyclohexane **2a** (64.7 µL, 0.5 mmol) gave product **3v** (77% yield) as a slightly yellow solid. Eluent: cyclohexane: ethyl acetate from 100:0 to 80:20.

^{1}H-NMR: δ 8.21 – 8.05 (m, 2H), 7.70 – 7.57 (m, 1H), 7.57 – 7.42 (m, 1H), 3.43 (tt, *J* = 11.2, 3.3 Hz, 1H), 2.63 (s, 3H), 1.97 – 1.84 (m, 4H), 1.86 – 1.72 (m, 3H), 1.63 – 1.45 (m, 2H), 1.42 – 1.28 (m, 1H) ppm.

^{13}C-NMR: δ 157.3, 142.2, 133.0, 128.8, 128.0, 126.0 (2C), 124.8, 121.1, 41.4, 32.8 (2C), 27.1 (2C), 26.4, 18.7 ppm.

HRMS (ESI$^+$): calculated for $C_{16}H_{20}NO$ $[M-H]^+$: 242.1539; found: 242.1544.

2-Cyclohexyl-4,7-diphenyl-1,10-phenanthroline (3w)

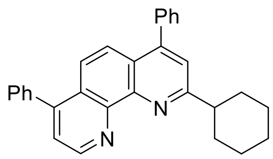

Following a slightly modified procedure A; 4,7-diphenyl-1,10-phenanthroline **1y** (33.2 mg, 0.1 mmol) and iodocyclohexane **2a** (64.7 µL, 0.5 mmol) gave product **3w** (35% yield) as a yellowish oil when the reaction was carried out at 5 mA. Eluent: cyclohexane: ethyl acetate from 100:0 to 50:50.

^{1}H-NMR: δ 9.28 (dd, *J* = 4.5, 0.7 Hz, 1H), 7.79 (dd, *J* = 3.0, 0.7 Hz, 2H), 7.59 – 7.47 (m, 12H), 3.41 (tt, *J* = 12.0, 3.4 Hz, 1H), 2.25 – 2.14 (m, 2H), 1.99 – 1.77 (m, 2H), 1.74 – 1.45 (m, 5H), 1.45 – 1.20 (m, 1H) ppm.

Spectra data are consistent with those reported in the literature.[10]

6-Cyclohexylphenanthridine (3x)

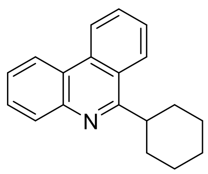

Following the general procedure A; phenanthridine **1o** (17.9 mg, 0.1 mmol) and iodocyclohexane **2a** (64.7 µL, 0.5 mmol) gave product **3x** (70% yield) as a white solid. Eluent: cyclohexane: ethyl acetate from 100:0 to 95:5.

^{1}H-NMR: δ 8.66 (d, *J* = 8.2 Hz, 1H), 8.54 (dd, *J* = 8.2, 1.3 Hz, 1H), 8.32 (d, *J* = 8.2 Hz, 1H), 8.14 (d, *J* = 7.6 Hz, 1H), 7.82 (t, *J* = 7.4 Hz, 1H), 7.75 – 7.66 (m, 2H), 7.64 – 7.57 (m, 1H), 3.62 (t, *J* = 11.3 Hz, 1H), 2.14 – 2.04 (m, 2H), 2.03 – 1.91 (m, 3H), 1.90 – 1.80 (m, 1H), 1.68 – 1.40 (m, 4H) ppm.

Spectra data are consistent with those reported in the literature.[6]

2-Cyclohexylbenzo[d]thiazole (3y)

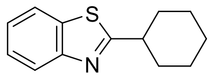

Following a slightly modified procedure A; benzo[*d*]thiazole **1p** (10.9 µL, 0.1 mmol) and iodocyclohexane **2a** (64.7 µL, 0.5 mmol) gave product **3y** (60% yield) as a colorless oil when the reaction was carried out during 8 hours. Eluent: cyclohexane: ethyl acetate from 100:0 to 95:5.

^{1}H-NMR: δ 8.00 – 7.93 (m, 1H), 7.89 – 7.80 (m, 1H), 7.48 – 7.40 (m, 1H), 7.38 – 7.29 (m, 1H), 3.11 (tt, *J* = 11.6, 3.6 Hz, 1H), 2.31 – 2.15 (m, 2H), 1.99 – 1.83 (m, 2H), 1.82 – 1.59 (m, 3H), 1.53 – 1.22 (m, 3H) ppm.

Spectra data are consistent with those reported in the literature.[6]

2-Cyclohexyl-1-methyl-1*H*-benzo[*d*]imidazole (3z)

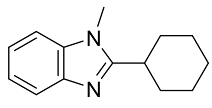

Following a slightly modified procedure A; 1-methyl-1*H*-benzo[*d*]imidazole **1q** (13.2 mg, 0.1 mmol) and iodocyclohexane **2a** (64.7 µL, 0.5 mmol) gave product **3z** (56% yield) as a colorless oil when the reaction was carried out during 8 hours. Eluent: cyclohexane: ethyl acetate from 100:0 to 90:10.

^{1}H-NMR: δ 7.80 – 7.70 (m, 1H), 7.33 – 7.18 (m, 3H), 3.75 (s, 3H), 2.85 (tt, *J* = 11.6, 3.4 Hz, 1H), 2.05 – 1.87 (m, 4H), 1.87 – 1.73 (m, 3H), 1.52 – 1.32 (m, 3H) ppm.

Spectra data are consistent with those reported in the literature.[3]

5,7-Dichloro-2-cyclohexyl-4-(4-fluorophenoxy)quinoline (3aa)

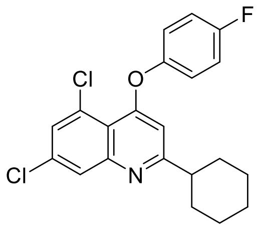

Following a slightly modified procedure A; 5,7-dichloro-4-(4-fluorophenoxy)quinoline **1r** (30.8 mg, 0.1 mmol) and iodocyclohexane **2a** (64.7 µL, 0.5 mmol) gave product **3aa** (45% yield) as a white solid when the reaction was carried out at 5 mA. Eluent: cyclohexane: ethyl acetate from 100:0 to 95:5 to 90:10.

^{1}H-NMR: δ 7.96 (d, *J* = 2.1 Hz, 1H), 7.51 (d, *J* = 2.1 Hz, 1H), 7.21 – 7.05 (m, 4H), 6.52 (s, 1H), 2.78 – 2.58 (m, 1H), 1.94 – 1.69 (m, 5H), 1.54 – 1.21 (m, 5H) ppm.

Spectra data are consistent with those reported in the literature.[3]

(*R*)-(2-Cyclohexyl-6-methoxyquinolin-4-yl)((1*S*,2*S*,4*S*,5*R*)-5-vinylquinuclidin-2-yl)methanol (3ab)

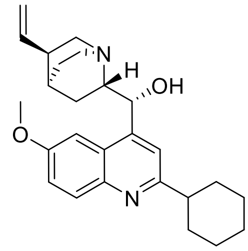

Following a slightly modified procedure A; (*R*)-(6-methoxyquinolin-4-yl)((1*S*,2*S*,4*S*,5*R*)-5-vinylquinuclidin-2-yl)methanol **1s** (32.4 mg, 0.1 mmol) and iodocyclohexane **2a** (64.7 µL, 0.5 mmol) gave product **3ab** (74% yield) as a white solid when it was carried out during 4 hours. Eluent: dichloromethane: methanol from 97:3 to 93:7.

^{1}H-NMR: δ 7.96 (d, *J* = 9.2 Hz, 1H), 7.48 (s, 1H), 7.31 (dd, *J* = 9.2, 2.6 Hz, 1H), 7.19 (s, 1H), 5.82 – 5.66 (m, 1H), 5.62 – 5.56 (m, 1H), 5.04 – 4.88 (m, 2H), 3.88 (s, 3H), 3.58 – 3.41 (m, 1H), 3.20 – 3.05 (m, 2H), 2.91 – 2.77 (m, 1H), 2.76 – 2.61 (m, 2H), 2.38 – 2.19 (m, 1H), 2.07 – 1.92 (m, 3H), 1.94 – 1.68 (m, 6H), 1.61 – 1.44 (m, 4H), 1.37 – 1.19 (m, 3H) ppm.

Spectra data are consistent with those reported in the literature.[6]

(*R*)-(2-Cyclohexylquinolin-4-yl)((1*S*,2*S*,4*S*,5*R*)-5-vinylquinuclidin-2-yl)methanol (3ac)

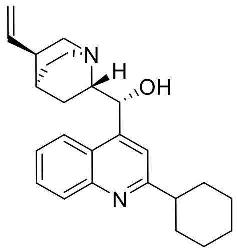

Following a slightly modified procedure A; (*R*)-quinolin-4-yl((1*S*,2*S*,4*S*,5*R*)-5-vinylquinuclidin-2-yl)methanol **1t** (29.4 mg, 0.1 mmol) and iodocyclohexane **2a** (64.7 µL, 0.5 mmol) gave product **3ac** (71% yield) as a white solid when the reaction was carried out during 4 hours. Eluent: dichloromethane: methanol from 97:3 to 93:7.

^{1}H-NMR: δ 8.07 (d, *J* = 8.3 Hz, 1H), 7.94 (d, *J* = 8.2 Hz, 1H), 7.65 (ddd, *J* = 8.3, 7.0, 1.2 Hz, 1H), 7.53 (s, 1H), 7.44 (ddd, *J* = 8.2, 7.0, 1.2 Hz, 1H), 5.82 – 5.63 (m, 2H), 5.03 – 4.86 (m, 2H), 3.54 –

3.39 (m, 1H), 3.18 – 3.04 (m, 2H), 2.90 (tt, *J* = 12.0, 3.4 Hz, 1H), 2.75 – 2.60 (m, 2H), 2.32 – 2.20 (m, 1H), 2.04 – 1.95 (m, 2H), 1.94 – 1.83 (m, 2H), 1.83 – 1.59 (m, 7H), 1.59 – 1.38 (m, 5H) ppm.

^{13}C-NMR: δ 166.7, 149.4, 148.0, 142.0, 129.9, 129.0, 125.9, 124.4, 122.7, 116.7, 114.5, 72.1, 60.4, 57.3, 47.9, 43.5, 40.1, 33.0, 28.2, 27.7, 27.1, 26.7 (2C), 26.2, 21.0 ppm.

HRMS (ESI$^+$): calculated for $C_{25}H_{33}N_2O$ [M-H]$^+$: 377.2587; found: 377.2518.

(*S*)-11-Cyclohexyl-4-ethyl-4-hydroxy-1,12-dihydro-14*H*-pyrano[3',4':6,7]indolizino[1,2-*b*] quinoline-3,14(4*H*)-dione (3ad)

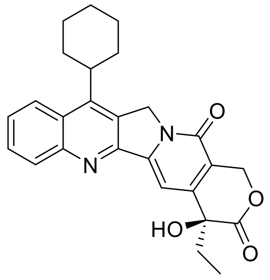

Following a slightly modified procedure A; (*S*)-4-ethyl-4-hydroxy-1,12-dihydro-14*H*-pyrano[3',4':6,7]indolizino[1,2-*b*]quinoline-3,14(4*H*)-dione **1u** (34.8 mg, 0.1 mmol) and iodocyclohexane **2a** (64.7 µL, 0.5 mmol) gave product **3ad** (38% yield) as a slightly yellow solid when the reaction was carried out at 5 mA. Eluent: dichloromethane: methanol from 100:0 to 95:5.

^{1}H-NMR: δ 8.29 – 8.19 (m, 2H), 7.79 (t, *J* = 7.0 Hz, 1H), 7.71 – 7.62 (m, 2H), 5.76 (d, *J* = 16.3 Hz, 1H), 5.42 (s, 1H), 5.31 (d, *J* = 16.3 Hz, 1H), 3.75 (bs, 1H), 3.64 (bs, 1H), 2.06 – 1.96 (m, 4H), 1.96 – 1.78 (m, 4H), 1.72 – 1.53 (m, 4H), 1.04 (t, *J* = 7.4 Hz, 3H) ppm.
Spectra data are consistent with those reported in the literature. [6]

Methyl 6-bromo-2-isopropylquinoline-4-carboxylate (6)

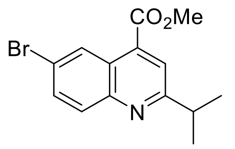

Following a slightly modified procedure A; ethyl 6-bromoquinoline-4-carboxylate **1h** (26.6 mg, 0.1 mmol) and 2-iodopropane **2b** (49.1 µL, 0.5 mmol) gave product **6** (39% yield) as a colorless oil when it was carried out at 5 mA for 90 minutes. Eluent: cyclohexane: ethyl acetate from 100:0 to 95:5.

^{1}H-NMR: δ 8.93 (d, *J* = 2.2 Hz, 1H), 7.96 (d, *J* = 9.0 Hz, 1H), 7.87 (s, 1H), 7.79 (dd, *J* = 9.0, 2.2 Hz, 1H), 4.05 (s, 3H), 3.38 – 3.16 (m, 1H), 1.40 (d, *J* = 6.9 Hz, 6H) ppm.

^{13}C-NMR: δ 167.7, 166.6, 147.6, 134.3, 133.2, 131.4, 126.0, 125.0, 121.9, 121.8, 53.0, 37.3, 22.4 (2C) ppm.

HRMS (ESI$^+$): calculated for $C_{14}H_{15}BrNO_2$ [M-H]$^+$: 308.0281; found: 308.0259.

Methyl 2-cyclohexylquinoline-4-carboxylate (8)

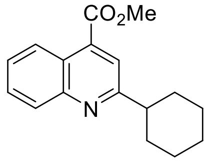

Following a slightly modified procedure A; ethyl quinoline-4-carboxylate **1i** (18.7 mg, 0.1 mmol) and iodocyclohexane **2a** (64.7 µL, 0.5 mmol) gave product **8** (53% yield) as a colorless oil when the reaction was carried out

during 1 hour. Eluent: cyclohexane: ethyl acetate from 100:0 to 95:5.

^{1}H-NMR: δ 8.68 (d, *J* = 8.2 Hz, 1H), 8.10 (d, *J* = 8.5 Hz, 1H), 7.83 (s, 1H), 7.76 – 7.68 (m, 1H), 7.62 – 7.53 (m, 1H), 4.04 (s, 3H), 2.96 (tt, *J* = 11.9, 3.4 Hz, 1H), 2.10 – 1.98 (m, 2H), 1.96 – 1.86 (m, 2H), 1.84 – 1.75 (m, 1H), 1.74 – 1.59 (m, 2H), 1.57 – 1.30 (m, 3H) ppm.

Spectra data are consistent with those reported in the literature. [7]

2-Cyclohexylquinoline-4-carboxylic acid (9)

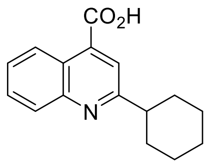

Following a slightly modified procedure A; quinoline-4-carboxylic acid **1k** (17.3 mg, 0.1 mmol) and iodocyclohexane **2a** (64.7 µL, 0.5 mmol) gave product **9** (30% yield) as a colorless oil when the reaction was carried out at 5 mA for 1 hour. Eluent: cyclohexane: ethyl acetate from 100:0 to 80:20.

^{1}H-NMR: δ 8.77 (d, *J* = 8.3 Hz, 1H), 8.14 (d, *J* = 8.3 Hz, 1H), 7.94 (s, 1H), 7.73 (t, *J* = 7.5 Hz, 1H), 7.58 (t, *J* = 7.5 Hz, 1H), 3.00 (t, *J* = 11.9 Hz, 1H), 2.08 – 2.00 (m, 2H), 1.96 – 1.85 (m, 2H), 1.83 – 1.74 (m, 1H), 1.73 – 1.58 (m, 2H), 1.56 – 1.29 (m, 3H) ppm.

Spectra data are consistent with those reported in the literature. [8]

3-Cyclohexyl-*N*,2-diphenylpropanamide (11)

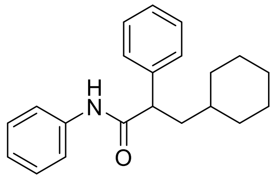

Following the general procedure A; *N*,2-diphenylacrylamide **10** (22.3 mg, 0.1 mmol) and iodocyclohexane **2a** (64.7 µL, 0.5 mmol) gave product **11** (70% yield) as a colorless oil. Eluent: cyclohexane: ethyl acetate from 100:0 to 95:5

^{1}H-NMR: δ 7.43 (d, *J* = 7.7 Hz, 2H), 7.36 (d, *J* = 4.3 Hz, 4H), 7.34 – 7.25 (m, 3H), 7.19 (s, 1H), 7.06 (t, *J* = 7.3 Hz, 1H), 3.65 (t, *J* = 7.6 Hz, 1H), 2.21 – 2.02 (m, 1H), 1.83 – 1.59 (m, 6H), 1.24 – 1.07 (m, 4H), 1.04 – 0.85 (m, 2H) ppm.

^{13}C-NMR: δ 172.1, 140.1, 138.1, 129.2 (2C), 129.1 (2C), 128.2 (2C), 127.6, 124.4, 119.9 (2C), 51.6, 40.9, 35.3, 33.8, 33.0, 26.7, 26.3, 26.2 ppm.

HRMS (ESI^{+}): calculated for $C_{21}H_{26}NO$ $[M-H]^{+}$: 308.2009; found: 308.2040.

4. General procedure B: Alkylation, allylation and benzylation of acridine

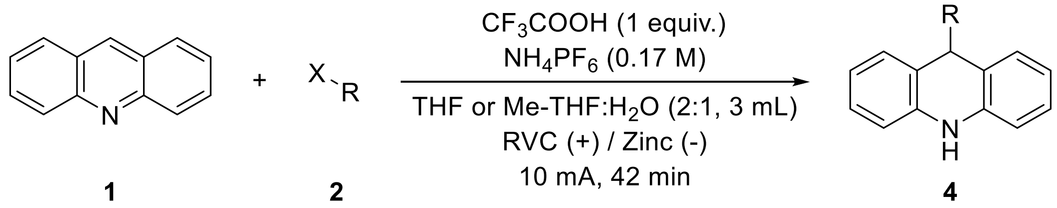

Acridine **1** (17.9 mg, 1 equiv.), ammonium hexafluorophosphate (82 mg, 5 equiv.) and a magnetic stirrer were added to a 5 mL ElectraSyn vial. Reagents were dissolved in 2 mL of THF (or Me-THF) and to the stirred solution were added trifluoroacetic acid (7.3 µL, 1 equiv.) and **2** (5 equiv.), followed by water (1 mL). The vial was closed, reticulated vitreous carbon was used as working electrode and zinc as counterelectrode, ElectraSyn 2.0 was set at constant current (10 mA) during 42 min. The crude mixture was then diluted with diethyl ether, extracted with saturated aqueous solution of $NaHCO_3$ (2 x 5 mL), wahsed with brine (3 x 30 mL), dried over anhydrous $MgSO_4$, filtered and the solvent was evaporated under reduced pressure. The crude mixture was purified by flash column chromatography using the eluent indicated in each case.

9-(*tert*-Butyl)-9,10-dihydroacridine (4a)

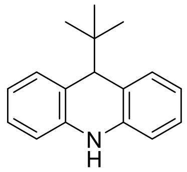

Following the general procedure B; acridine **1v** (17.9 mg, 0.1 mmol) and 2-iodo-2-methypropane **2g** (59.6 µL, 0.5 mmol) gave product **4a** (80% yield) as a white solid. Eluent: cyclohexane: ethyl acetate from 100:0 to 95:5.

^{1}H-NMR: δ 7.18 – 7.10 (m, 4H), 6.94 – 6.87 (m, 2H), 6.79 – 6.73 (m, 2H), 6.00 (s, 1H), 3.63 (s, 1H), 0.81 (s, 9H) ppm.

^{13}C-NMR: δ 141.4 (2C), 131.3 (2C), 127.0 (2C), 121.8 (2C), 120.1 (2C), 113.7 (2C), 53.4, 38.5, 27.5 (3C) ppm.

HRMS (ESI$^+$): calculated for $C_{17}H_{20}N$ $[M-H]^+$: 238.1590; found: 238.1603.

9-Allyl-9,10-dihydroacridine (4b)

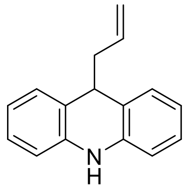

Following a modified procedure B; acridine **1v** (17.9 mg, 0.1 mmol) and 3-bromoprop-1-ene **2j** (86.6 µL, 1.0 mmol) when employing ammonium hexafluorophosphate (164 mg, 10 equiv.) and trifluoroacetic acid (14.5 µL, 2 equiv.) with a Ni foam counterelectrode instead of Zn, gave product **4b** (62% yield) as a slightly yellow solid. Eluent: cyclohexane: ethyl acetate from 100:0 to 95:5.

^{1}H-NMR: δ 7.17 – 7.07 (m, 4H), 6.89 (td, *J* = 7.4, 1.0 Hz, 2H), 6.73 (d, *J* = 7.9 Hz, 2H), 6.03 (s, 1H), 5.71 (ddt, *J* = 17.1, 10.1, 6.9 Hz, 1H), 4.87 (dd, *J* = 19.1, 17.1 Hz, 1H), 4.03 (t, *J* = 6.9 Hz, 1H), 2.36 (t, *J* =6.9 Hz, 2H) ppm.

^{13}C-NMR: δ 139.8 (2C), 135.9, 129.0 (2C), 127.1 (2C), 123.8 (2C), 120.8 (2C), 117.0, 113.6 (2C), 44.4, 43.1 ppm.

HRMS (ESI^{+}): calculated for $C_{16}H_{16}N$ $[M-H]^{+}$: 222.1277; found: 222.1237.

9-Benzyl-9,10-dihydroacridine (4c)

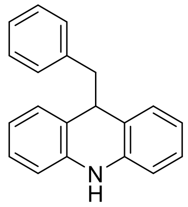

Following a slightly modified procedure B; acridine **1v** (17.9 mg, 0.1 mmol) and (bromomethyl)benzene **2k** (59.5 µL, 0.5 mmol) in Me-THF:H_2O (2:1) gave product **4c** (81% yield) as a white solid. Eluent: cyclohexane: ethyl acetate from 100:0 to 90:10.

^{1}H-NMR: δ 7.18 – 7.14 (m, 3H), 7.13 – 7.07 (m, 2H), 6.87 (dd, *J* = 7.4, 1.2 Hz, 2H), 6.84 – 6.75 (m, 4H), 6.70 (d, *J* = 7.9 Hz, 2H), 5.99 (s, 1H), 4.18 (t, *J* = 7.0 Hz, 1H), 2.85 (d, *J* = 7.0 Hz, 2H) ppm.

^{13}C-NMR: δ 139.9 (2C), 139.2, 130.1 (2C), 129.1 (2C), 127.9 (2C), 127.1 (2C), 126.1, 123.5 (2C), 120.6 (2C), 113.5 (2C), 46.2, 45.4 ppm.

HRMS (ESI^{+}): calculated for $C_{20}H_{18}N$ $[M-H]^{+}$: 272.1434; found: 272.1494.

9-(4-Chlorobenzyl)-9,10-dihydroacridine (4d)

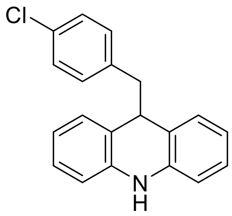

Following a slightly modified procedure B; acridine **1v** (17.9 mg, 0.1 mmol) and 1-(bromomethyl)-4-chlorobenzene **2l** (102.7 mg, 0.5 mmol) in Me-THF:H_2O (2:1) gave product **4d** (85% yield) as a white solid. Eluent: cyclohexane: ethyl acetate from 100:0 to 95:5.

^{1}H-NMR: δ 7.15 – 7.00 (m, 4H), 6.91 (dd, *J* = 7.4, 1.3 Hz, 2H), 6.83 (td, *J* = 7.4, 1.1 Hz, 2H), 6.75 – 6.60 (m, 4H), 5.94 (s, 1H), 4.19 (t, *J* = 6.7 Hz, 1H), 2.82 (d, *J* = 6.7 Hz, 1H) ppm.

^{13}C-NMR: δ 139.9 (2C), 137.5, 132.0, 131.4 (2C), 129.0 (2C), 128.0 (2C), 127.3 (2C), 123.0 (2C), 120.7 (2C), 113.5 (2C), 45.6, 45.1 ppm.

HRMS (ESI^{+}): calculated for $C_{20}H_{17}ClN$ $[M-H]^{+}$: 306.1044; found: 306.1045.

9-(2-Iodobenzyl)-9,10-dihydroacridine (4e)

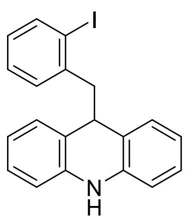

Following a slightly modified procedure B; acridine **1v** (17.9 mg, 0.1 mmol) and 1-(bromomethyl)-2-iodobenzene **2m** (148.5 mg, 0.5 mmol) in Me-THF:H_2O (2:1) gave product **4e** (65% yield) as a yellowish solid. Eluent: cyclohexane: ethyl acetate from 100:0 to 80:20.

^{1}H-NMR: δ 7.84 (dd, *J* = 7.9, 1.2 Hz, 1H), 7.16 – 7.09 (m, 2H), 7.05 (td, *J* = 7.4, 1.2 Hz, 1H), 6.91 – 6.75 (m, 7H), 6.49 (dd, *J* = 7.6, 1.7 Hz, 1H), 6.16 (s, 1H), 4.32 (t, *J* = 7.6 Hz, 1H), 2.94 (d, *J* = 7.6 Hz, 1H) ppm.

^{13}C-NMR: δ 141.7, 140.0 (2C), 139.4, 132.1, 129.2 (2C), 128.1, 127.7, 127.3 (2C), 123.2 (2C), 120.7 (2C), 113.6 (2C), 101.4, 49.4, 43.1 ppm.

HRMS (ESI$^+$): calculated for $C_{20}H_{17}IN$ $[M-H]^+$: 398.0400; found: 396.0370.

9-(4-(Trifluoromethyl)benzyl)-9,10-dihydroacridine (4f)

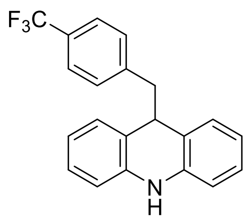

Following a slightly modified procedure B; acridine **1v** (17.9 mg, 0.1 mmol) and 1-(bromomethyl)-4-(trifluoromethyl)benzene **2n** (119.5 mg, 0.5 mmol) in Me-THF:H_2O (2:1) gave product **4f** (53% yield) as a slightly yellow solid. Eluent: cyclohexane: ethyl acetate from 100:0 to 95:5.

^{1}H-NMR: δ 7.37 (d, *J* = 8.0 Hz, 2H), 7.11 (td, *J* = 7.9, 1.7 Hz, 2H), 6.92 – 6.79 (m, 6H), 6.69 (d, *J* = 7.9 Hz, 2H), 5.95 (s, 1H), 4.23 (t, *J* = 6.7 Hz, 1H), 2.89 (d, *J* = 6.7 Hz, 2H) ppm.

^{13}C-NMR: δ 143.2, 139.9 (2C), 130.3 (2C), 129.0 (2C), 128.6 (q, *J* = 9.0 Hz), 127.4 (2C), 127.3 (q, *J* = 137.1 Hz), 124.7 (q, *J* = 3.8 Hz, 2C), 122.8 (2C), 120.8 (2C), 113.6 (2C), 46.0, 45.0 ppm.

^{19}F-NMR: δ -62.1 ppm.

HRMS (ESI$^+$): calculated for $C_{21}H_{17}F_3N$ $[M-H]^+$: 340.1308; found: 340.1263.

9-(3,5-Dimethoxybenzyl)-9,10-dihydroacridine (4g)

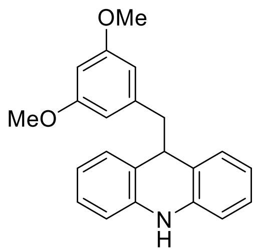

Following a slightly modified procedure B; acridine **1v** (17.9 mg, 0.1 mmol) and 1-(bromomethyl)-3,5-dimethoxybenzene **2o** (115.5 mg, 0.5 mmol) in Me-THF:H_2O (2:1) gave product **4g** (75% yield) as a white solid. Eluent: cyclohexane: ethyl acetate from 100:0 to 95:5.

^{1}H-NMR: δ 7.10 (td, *J* = 7.6, 1.5 Hz, 2H), 6.94 (d, *J* = 7.4 Hz, 2H), 6.83 (td, *J* = 7.4, 1.1 Hz, 2H), 6.69 (d, *J* = 7.9 Hz, 2H), 6.39 – 6.26 (m, 1H), 6.00 (s, 1H), 5.90 (d, *J* = 2.2 Hz, 2H), 4.19 (t, *J* = 6.8 Hz, 1H), 3.62 (s, 6H), 2.78 (d, *J* = 6.8 Hz, 2H) ppm.

^{13}C-NMR: δ 160.3 (2C), 141.3, 139.9 (2C), 129.1 (2C), 127.2 (2C), 123.4 (2C), 120.7 (2C), 113.5 (2C), 107.8 (2C), 106.7, 99.0, 55.3 (2C), 46.5, 45.2 ppm.

HRMS (ESI$^+$): calculated for $C_{22}H_{22}NO_2$ $[M-H]^+$: 332.1645; found: 332.1673.

9-(Benzo[*d*][1,3]dioxol-5-ylmethyl)-9,10-dihydroacridine (4h)

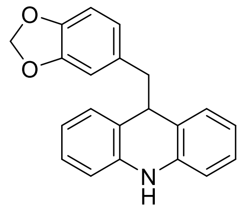

Following a slightly modified procedure B; acridine **1v** (17.9 mg, 0.1 mmol) and 5-(bromomethyl)benzo[*d*][1,3]dioxole **2p** (107.5 mg, 0.5 mmol) in Me-THF:H_2O (2:1) gave product **4h** (79% yield) as a white solid. Eluent: cyclohexane: ethyl acetate from 100:0 to 95:5.

^{1}H-NMR: δ 7.10 (t, *J* = 7.5 Hz, 2H), 6.90 (d, *J* = 7.5 Hz, 2H), 6.82 (t, *J* = 7.5 Hz, 2H), 6.70 (d, *J* = 7.5 Hz, 2H), 6.60 (d, *J* = 7.8 Hz, 1H), 6.30 (s, 1H), 6.20 (d, *J* = 7.8 Hz, 1H), 6.00 (s, 1H), 5.90 (s, 2H), 4.13 (t, *J* = 6.9 Hz, 1H), 2.75 (d, *J* = 6.9 Hz, 2H) ppm.

^{13}C-NMR: δ 147.2, 145.9, 139.8 (2C), 133.0, 129.1 (2C), 127.2 (2C), 123.4 (2C), 123.0, 120.7 (2C), 113.5 (2C), 110.4, 107.8, 100.8, 45.9, 45.5 ppm.

HRMS (ESI$^+$): calculated for $C_{21}H_{18}NO_2$ $[M-H]^+$: 316.1332; found: 316.1269.

5. General procedure C: Derivatizations

6-Bromo-2-isopropylquinoline-4-carboxylic acid (7)

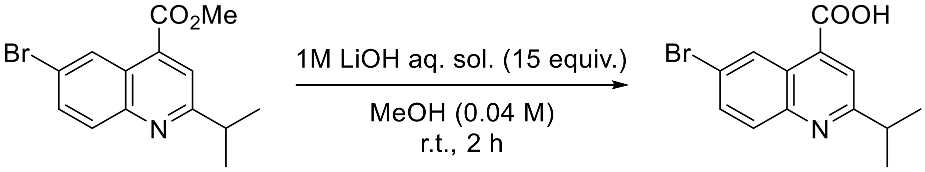

6 **7**

1M lithium hydroxide aqueous solution (0.3 mL) was added to a vial containing methyl 6-bromo-2-isopropylquinoline-4-carboxylate (**6**, 6 mg, 0.05 mmol) in methanol (0.5 mL). The reaction was stirred at room temperature for two hours. Then 1M HCl aqueous solution was added until pH= 1. The crude was extracted with EtOAc (2 x 5 mL). Organic layer was dried over anhydrous Na_2SO_4, filtered and concentrated. Crude was purified by flash column chromatography using cyclohexane: ethyl acetate/methanol/acetic acid (3/1/2%) from 100:0 to 80:20. 6-Bromo-2-isopropylquinoline-4-carboxylic acid (**7**, 80% yield) was obtained as a colorless oil.

^{1}H-NMR ($CDCl_3$ + few drops CD_3OD): δ 9.01 (d, *J* = 1.9 Hz, 1H), 8.05 – 7.90 (m, 2H), 7.79 (dd, *J* = 9.0, 2.0 Hz, 1H), 3.41 – 3.22 (m, 1H), 1.40 (d, *J* = 6.9 Hz, 6H) ppm.

^{13}C-NMR ($CDCl_3$ + few drops CD_3OD): δ 167.9 (2C), 147.3, 133.1 (2C), 130.9, 128.2, 125.3, 122.0, 121.6, 37.2, 22.3 (2C) ppm.

HRMS (ESI$^+$): calculated for $C_{13}H_{13}BrNO_2$ $[M-H]^+$: 293.0046; found: 293.0090.

9-(Benzo[d][1,3]dioxol-5-ylmethyl)acridine (5)

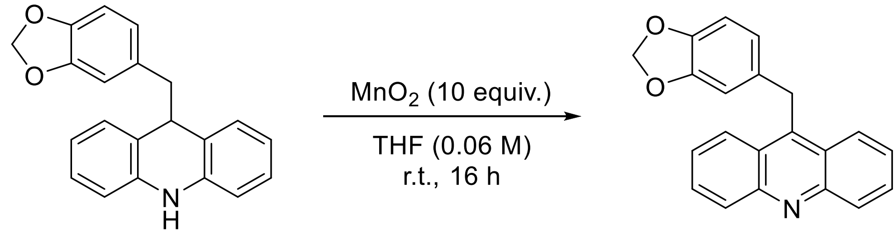

4h **5**

MnO_2 (55 mg, 10 equiv.) was added to a vial containing 9-(Benzo[*d*][1,3]dioxol-5-ylmethyl)-9,10-dihydroacridine (**4h**, 20 mg, 0.06 mmol) in tetrahydrofuran (1.0 mL). The reaction was stirred at room temperature 16 h. Crude was purified by flash column chromatography using cyclohexane: ethyl acetate from 100:0 to 95:5 to 90:10. 9-(Benzo[d][1,3]dioxol-5-ylmethyl)acridine (**5**, 85% yield) was obtained as a white solid.

^{1}H-NMR: δ 8.31 – 8.18 (m, 4H), 7.84 – 7.71 (m, 2H), 7.59 – 7.48 (m, 2H), 6.69 – 6.63 (m, 1H), 6.62 – 6.54 (m, 2H), 5.86 (s, 2H), 4.91 (s, 2H) ppm.

^{13}**C-NMR**: δ 149.1 (2C), 148.1, 146.3, 143.6, 133.4, 130.6 (2C), 130.0 (2C), 126.3 (2C), 125.8 (2C), 124.8 (2C), 121.3, 108.8, 108.6, 101.1, 32.9 ppm.

HRMS (ESI$^+$): calculated for $C_{21}H_{16}NO_2$ $[M-H]^+$: 314.1176; found: 314.1157.

6. Nuclear magnetic resonance spectra

2-Cyclohexyl-4-methylquinoline (3a)

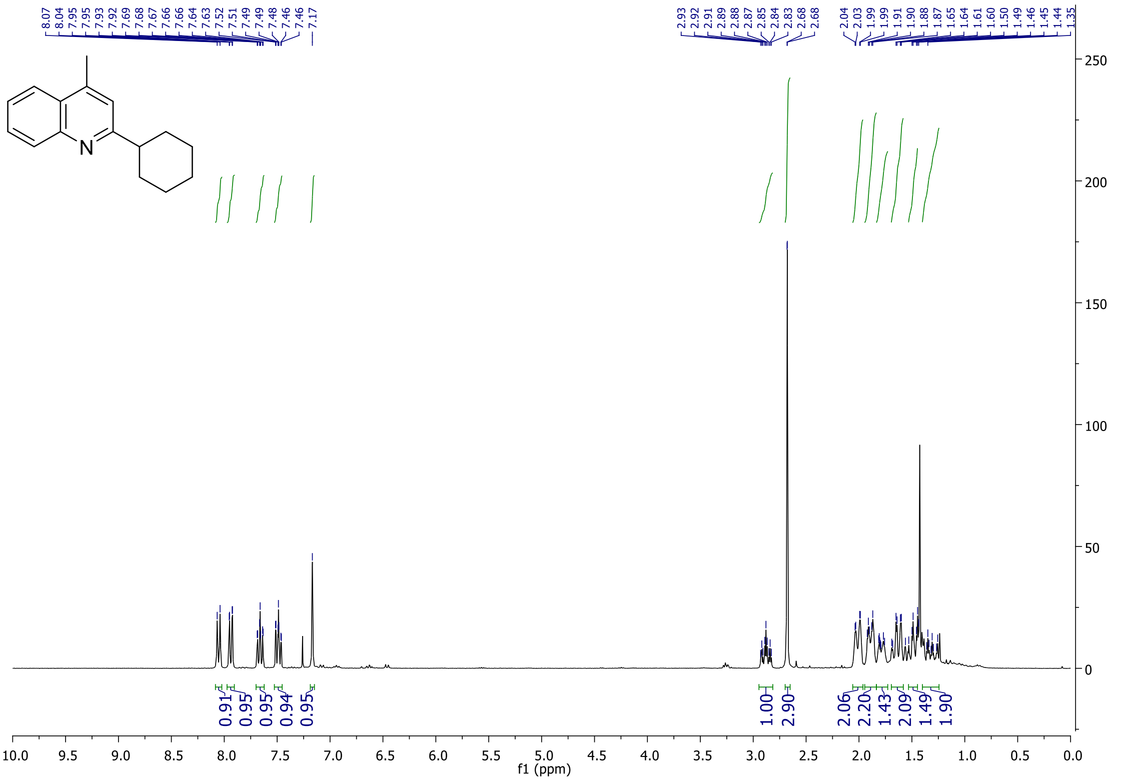

2-Isopropyl-4-methylquinoline (3b)

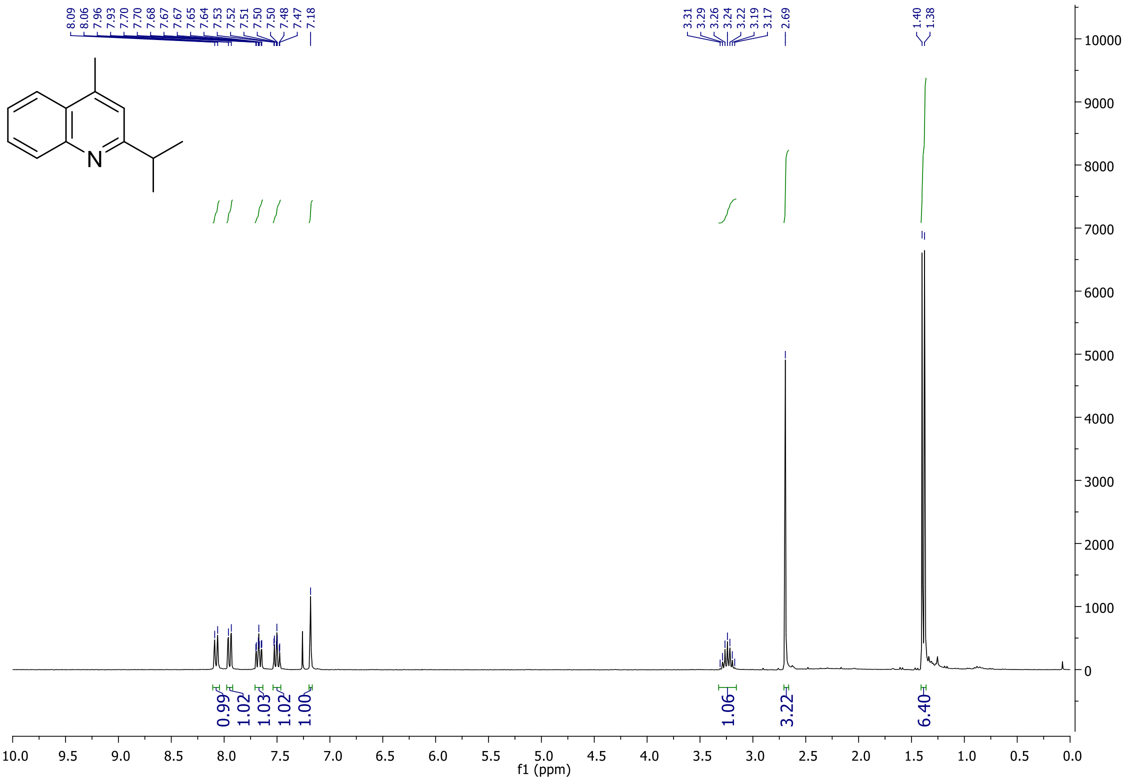

4-Methyl-2-(tetrahydro-2*H*-pyran-4-yl)quinoline (3c)

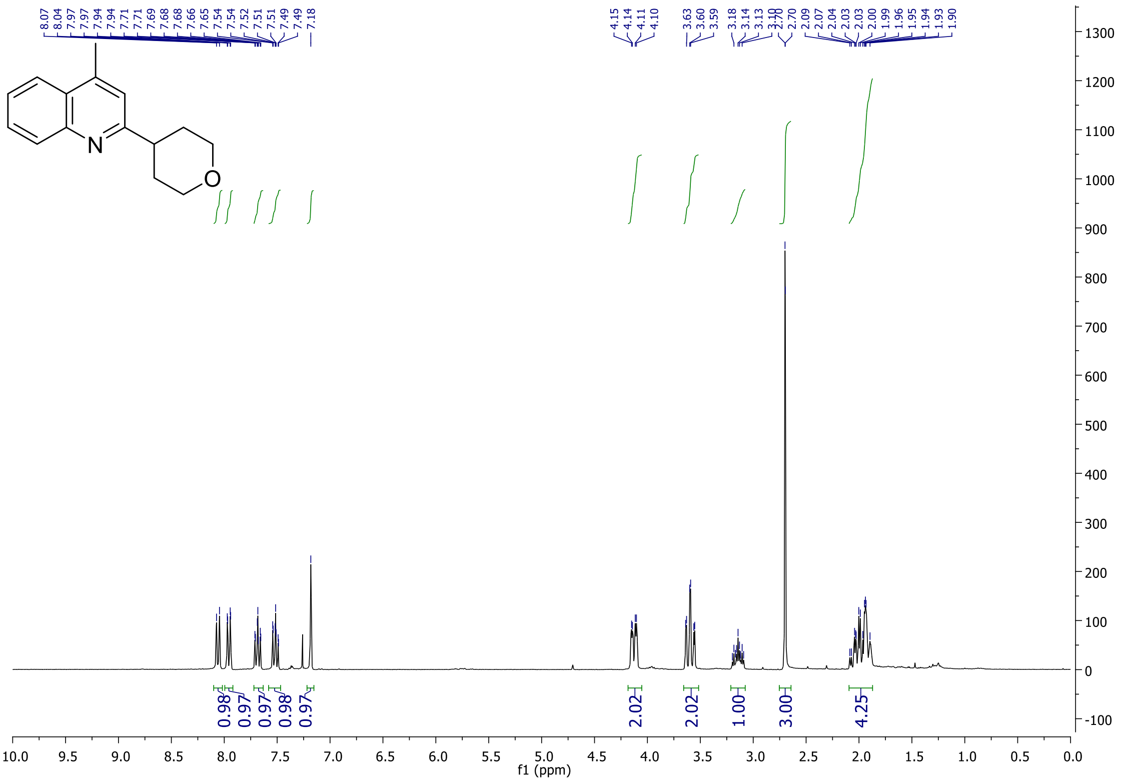

tert-Butyl 4-(4-methylquinolin-2-yl)piperidine-1-carboxylate (3d)

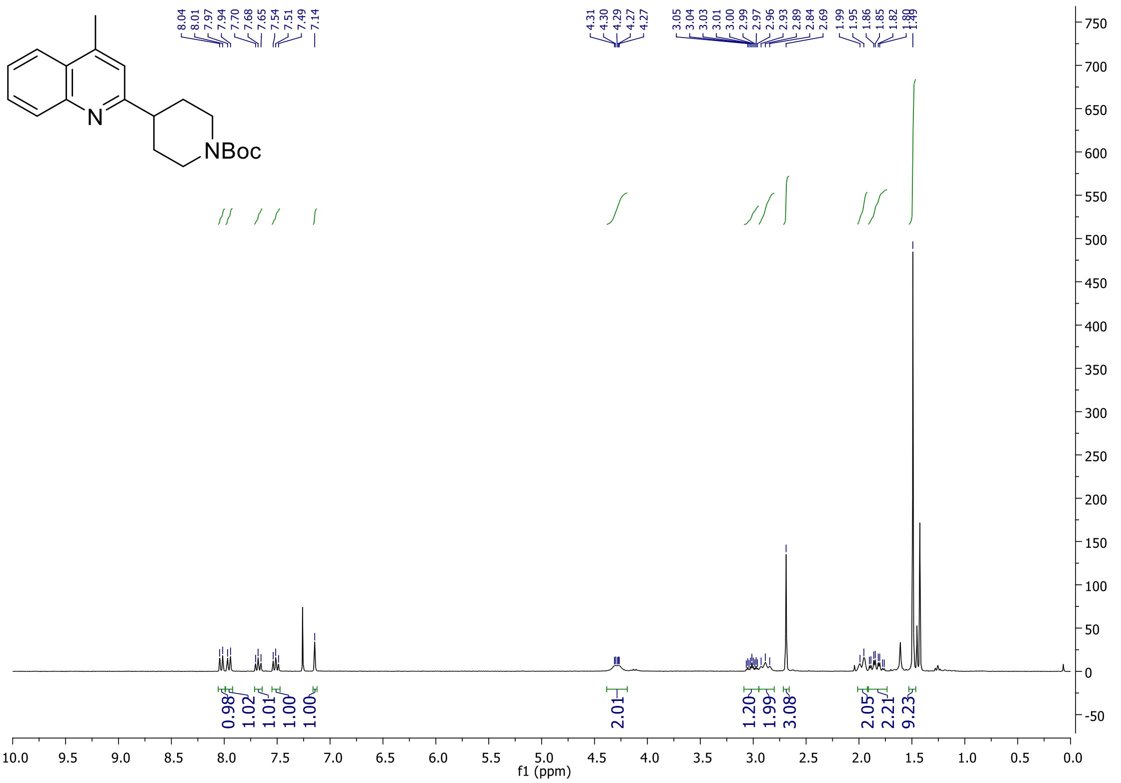

2-Cyclopentyl-4-methylquinoline (3e)

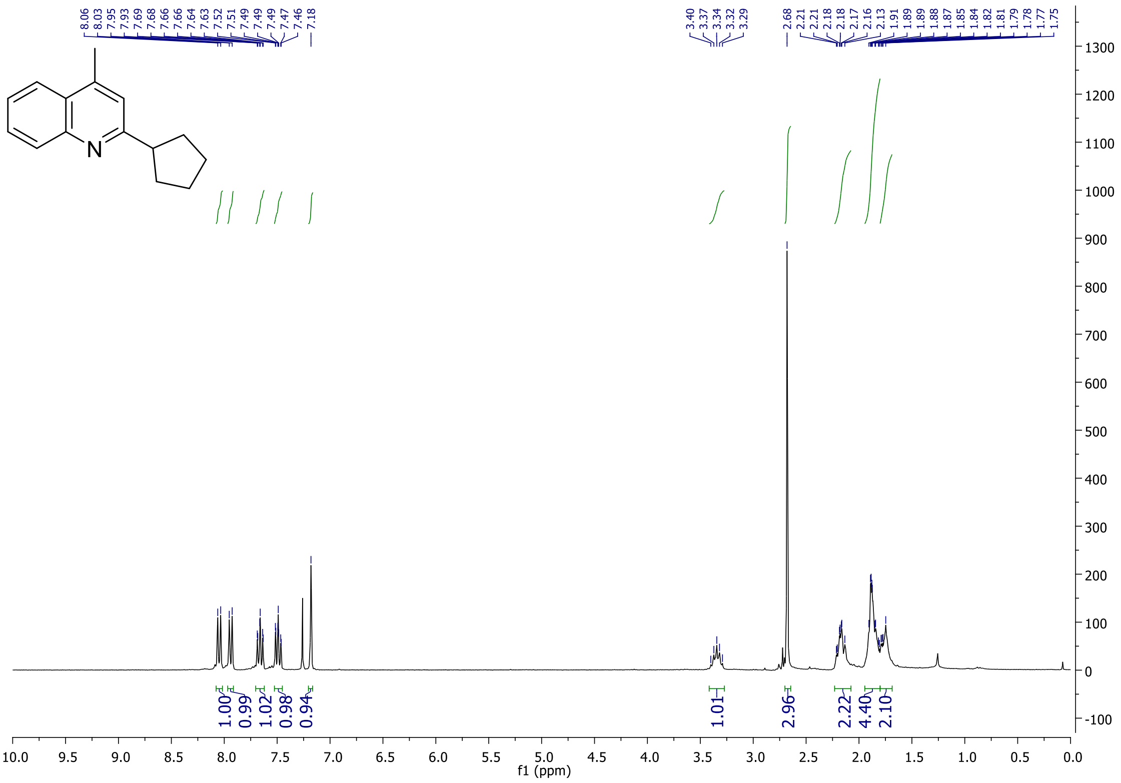

4-Methyl-2-(tetrahydrofuran-3-yl)quinoline (3f)

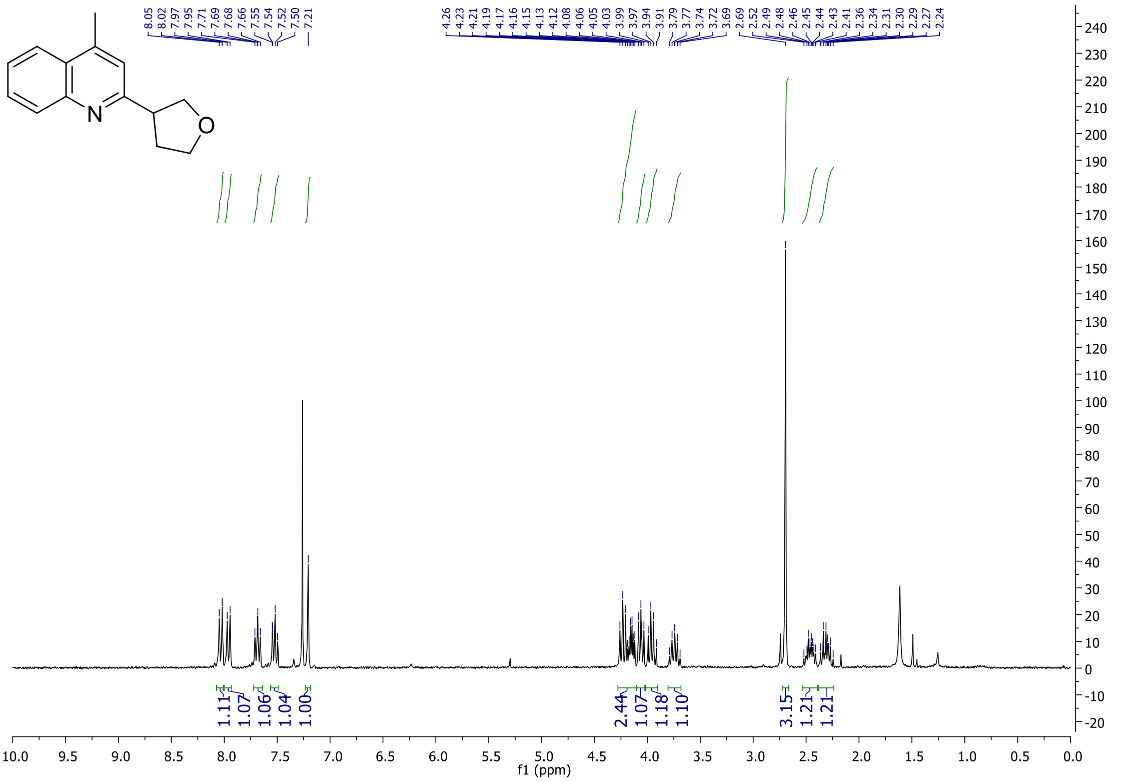

2-(*tert*-Butyl)-4-methylquinoline (3g)

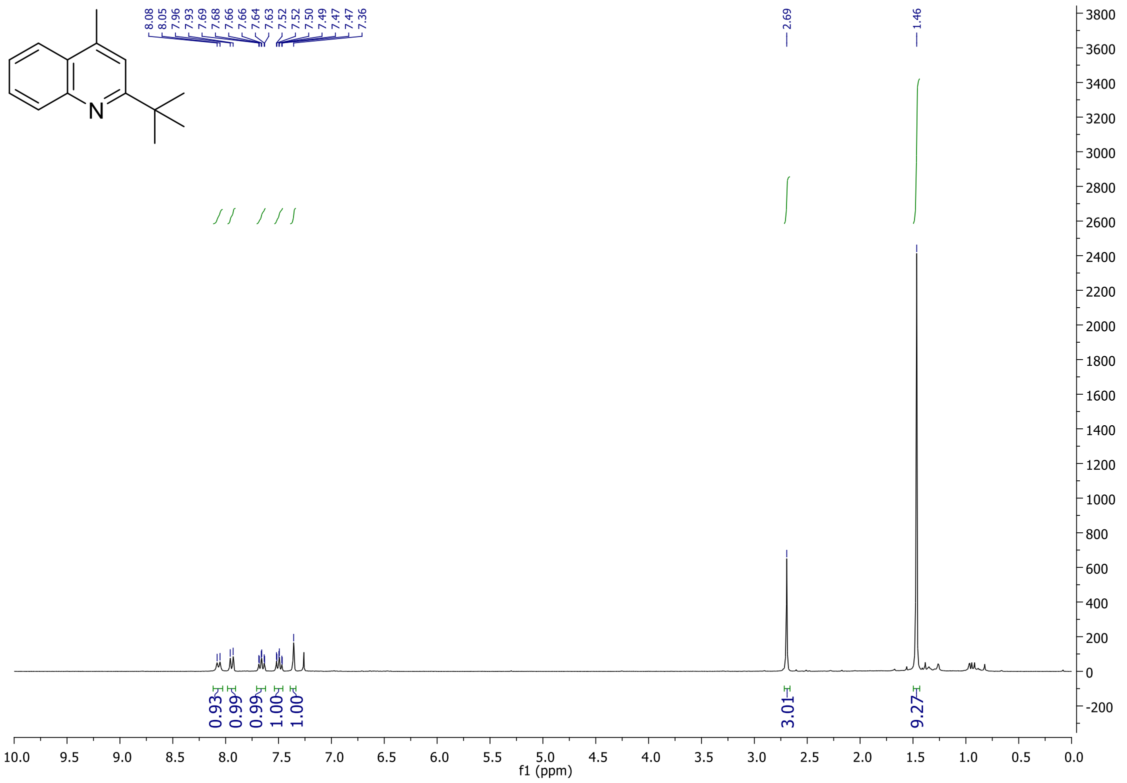

2-Ethyl-4-methylquinoline (3h)

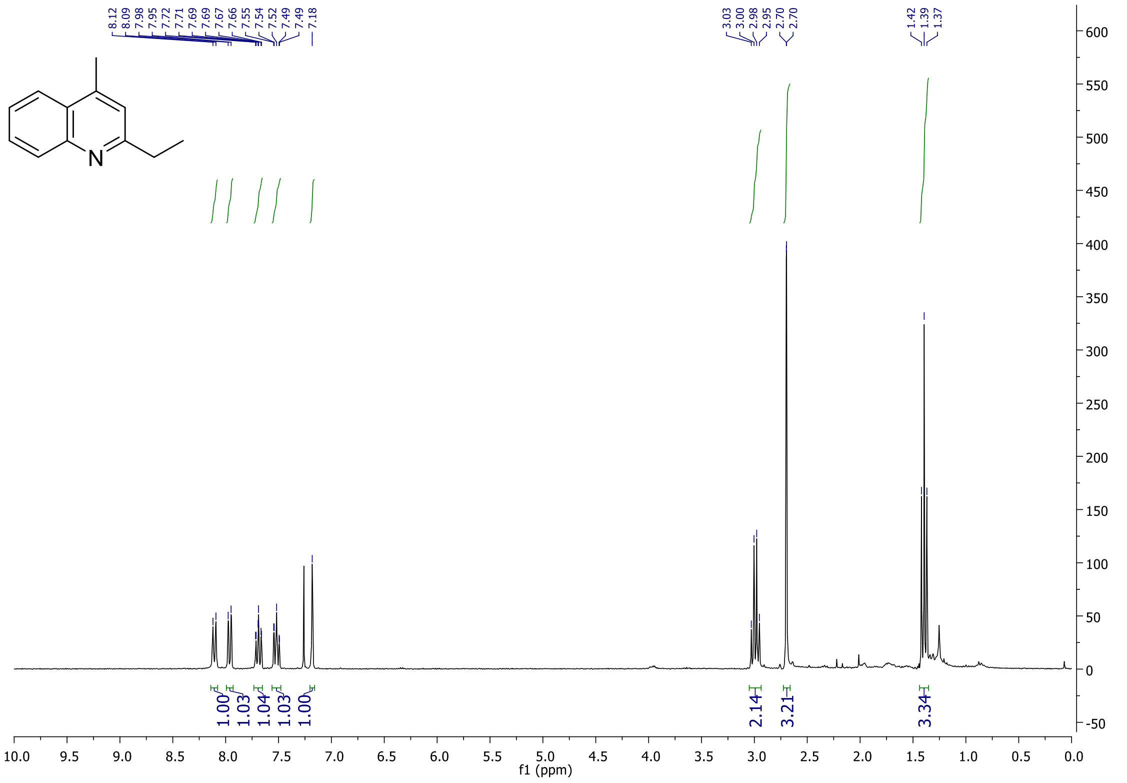

4-Methyl-2-phenethylquinoline (3i)

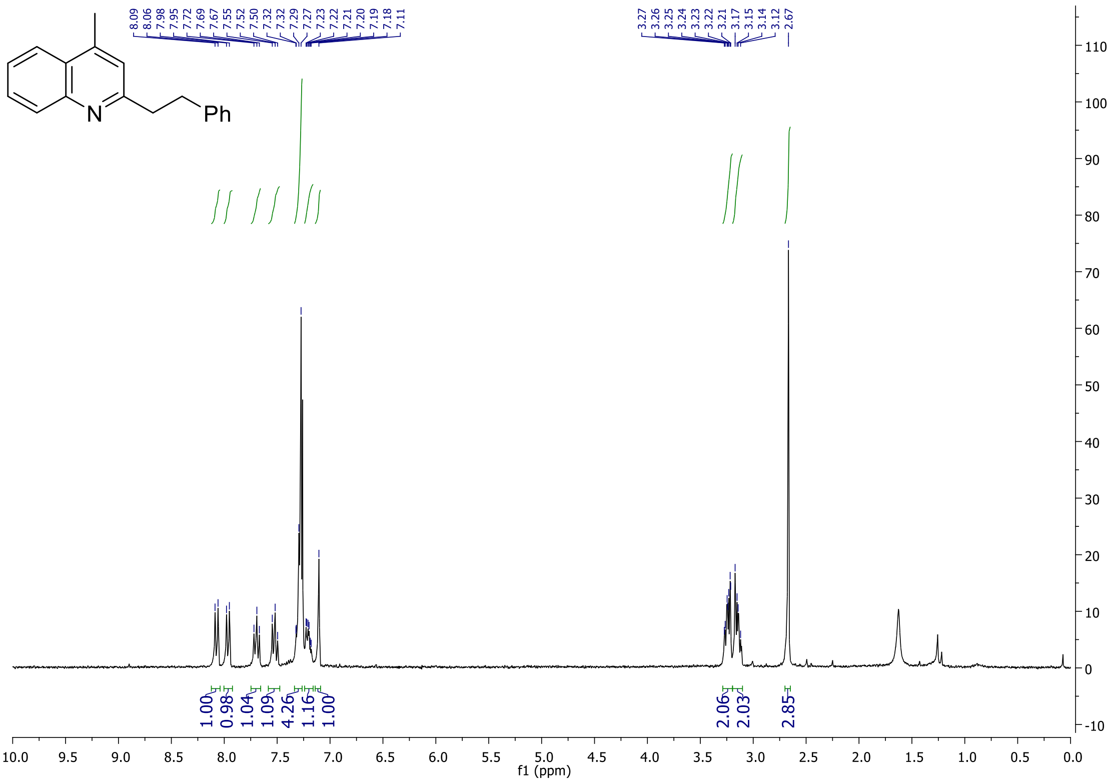

2-Cyclohexyl-7-methoxy-4-methylquinoline (3j)

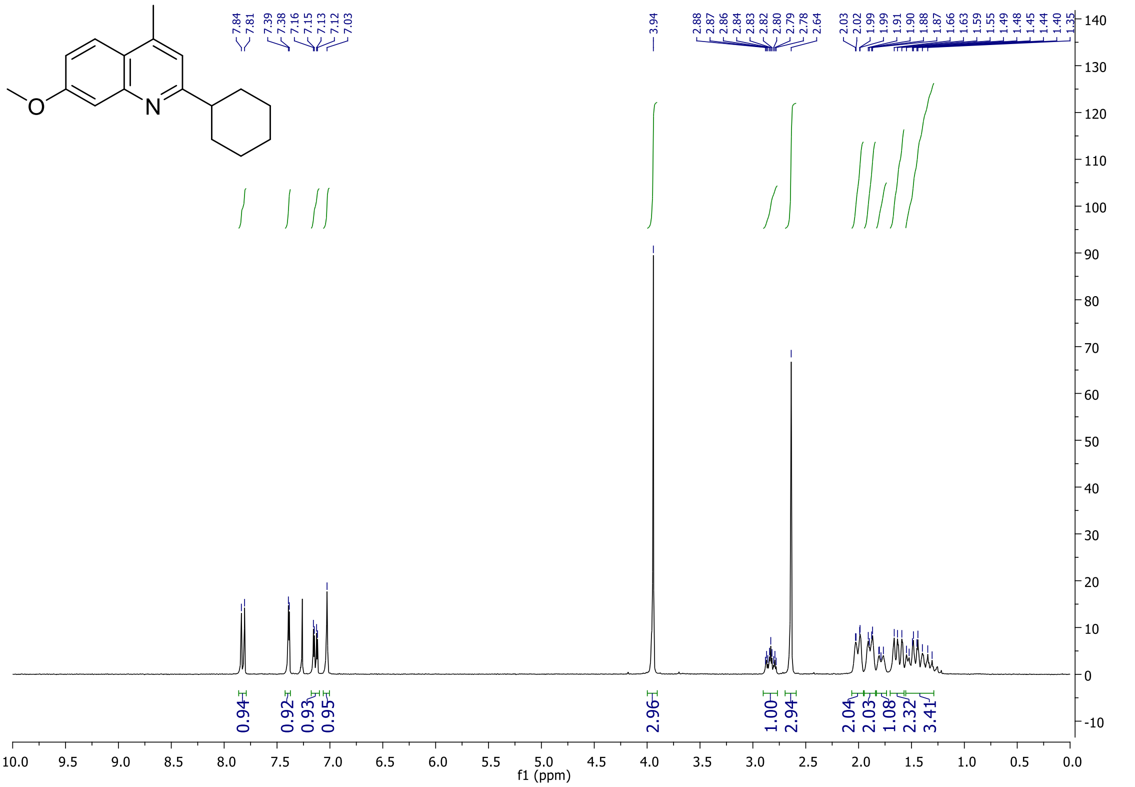

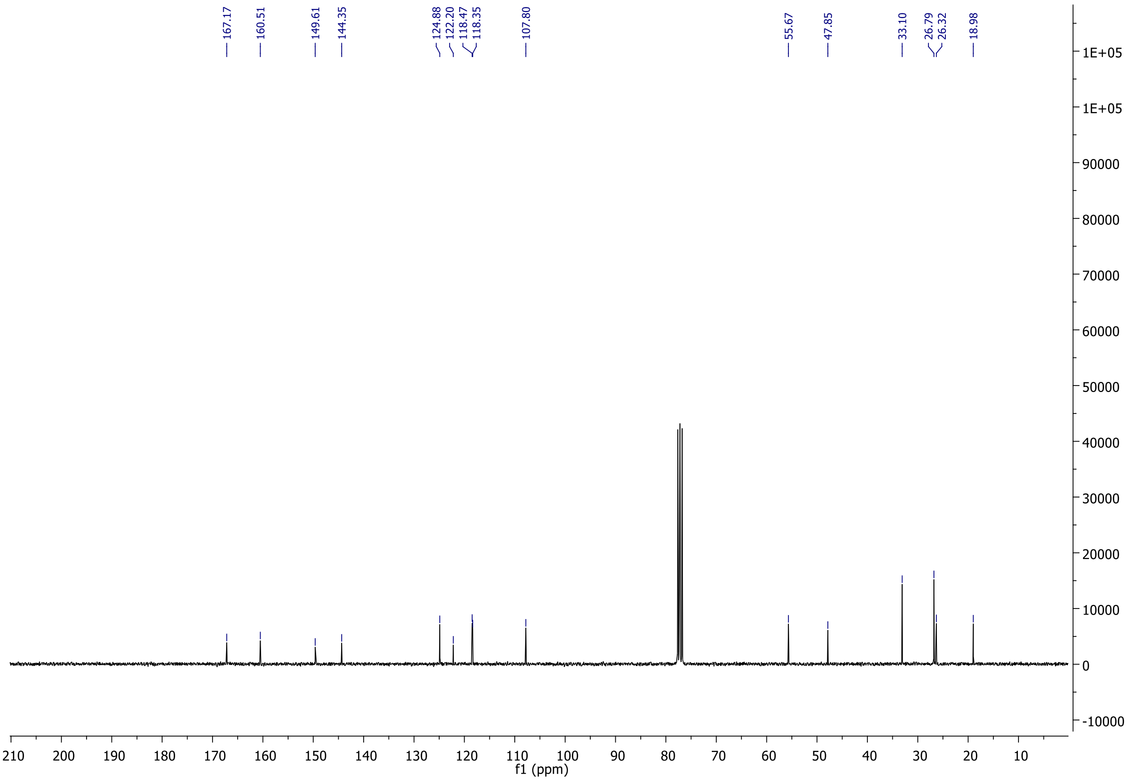

7-Bromo-2-cyclohexyl-4-methylquinoline (3k)

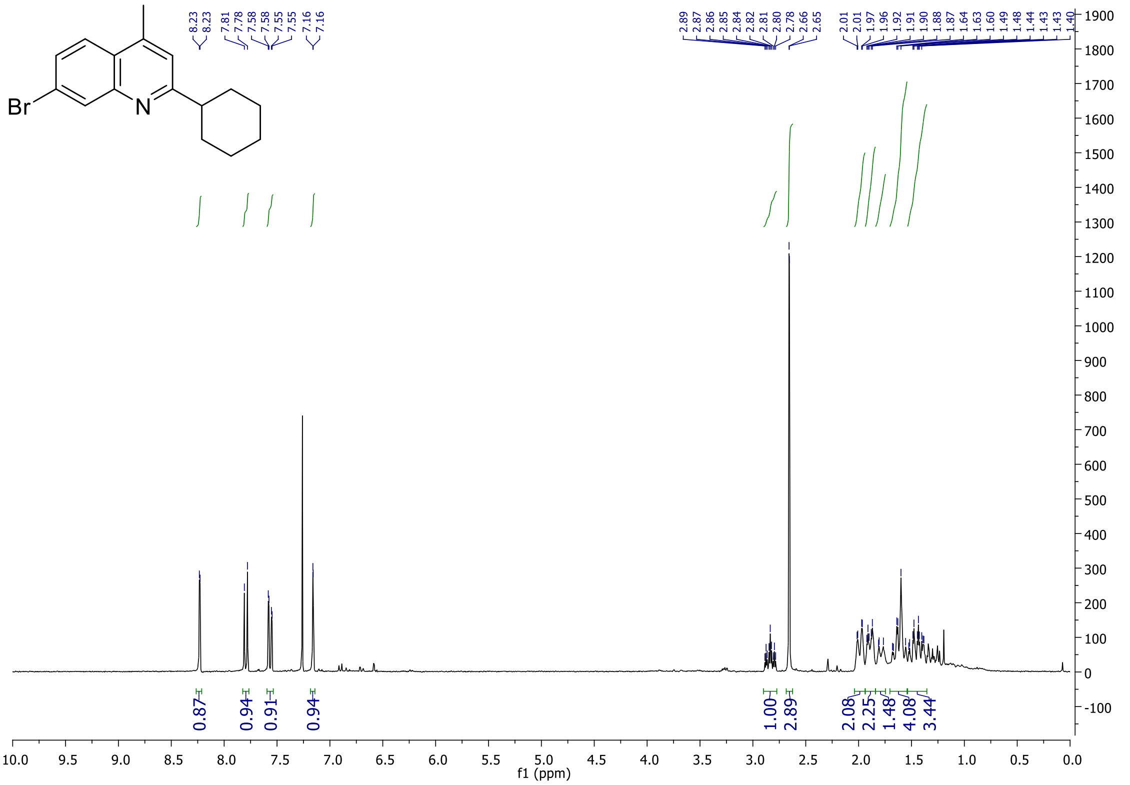

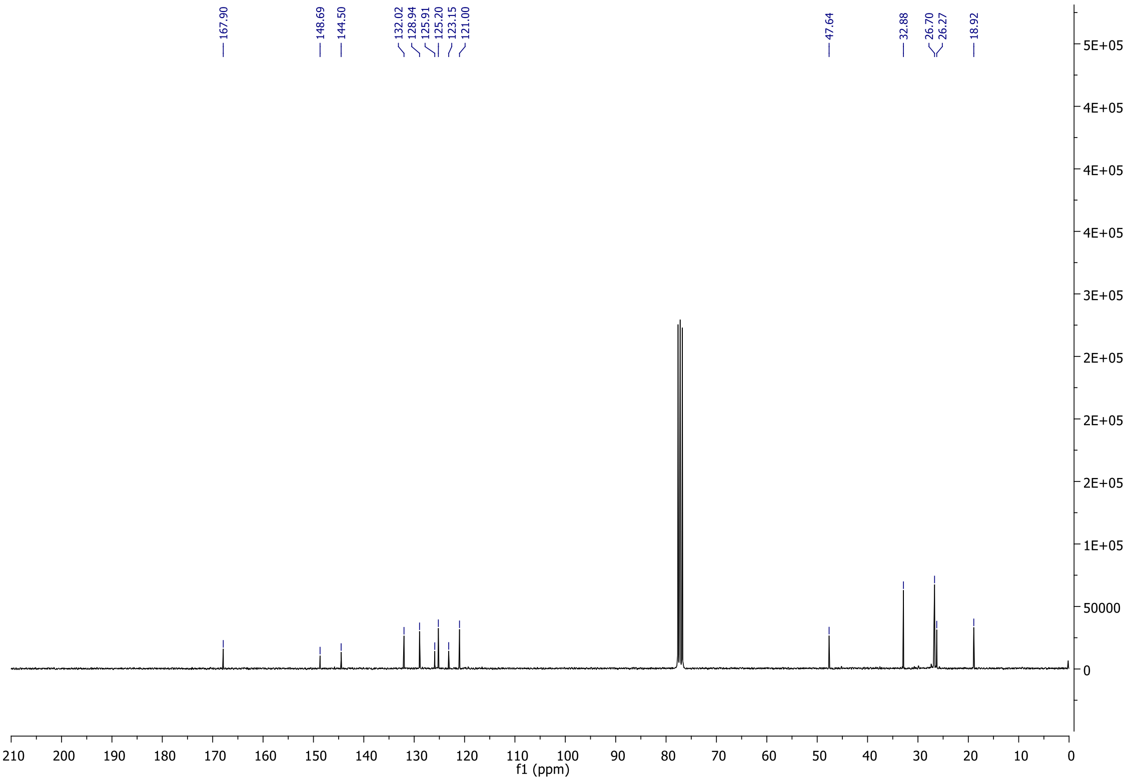

6-Bromo-2-cyclohexyl-4-methylquinoline (3l)

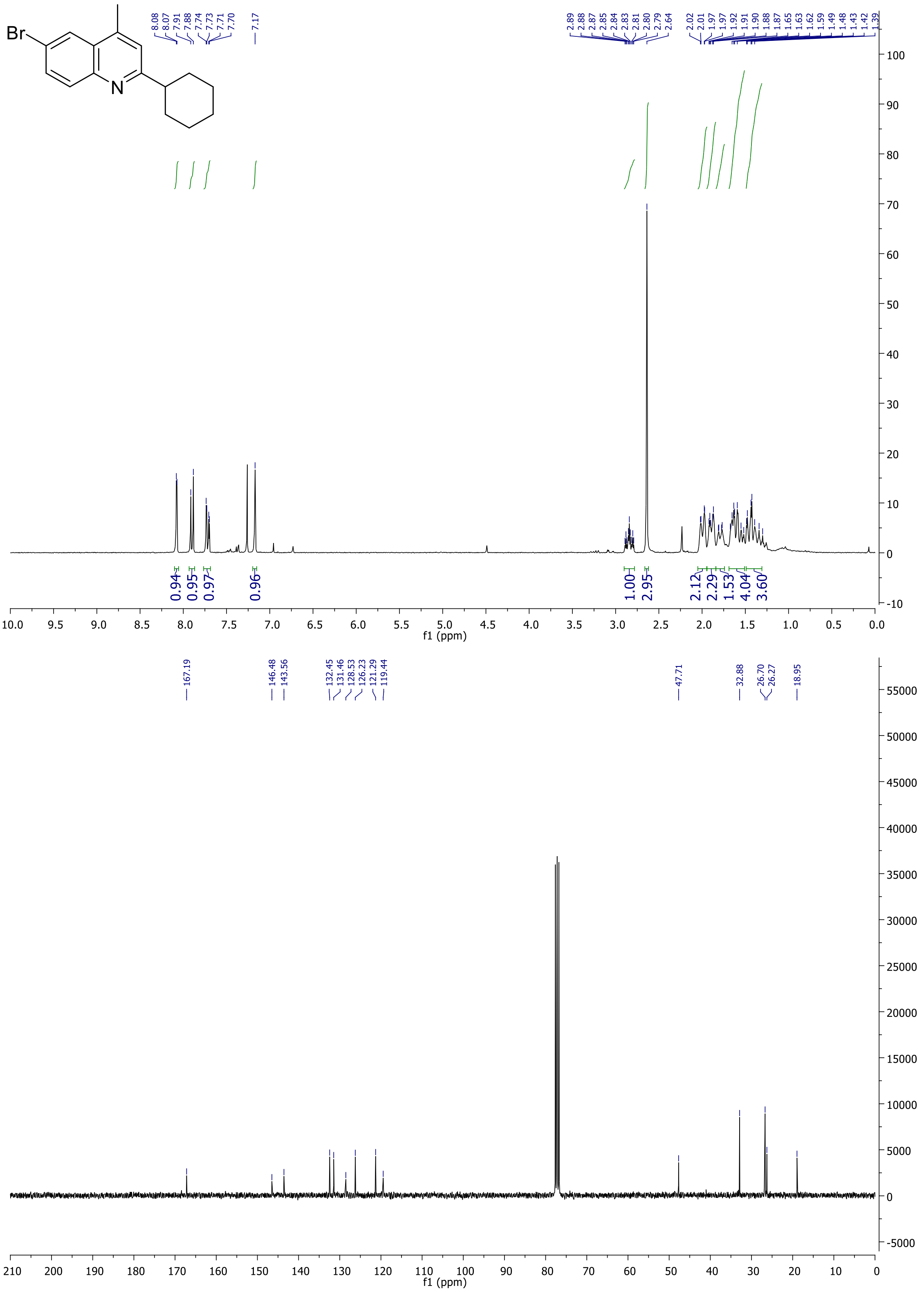

2-Cyclohexyl-4-phenylquinoline (3m)

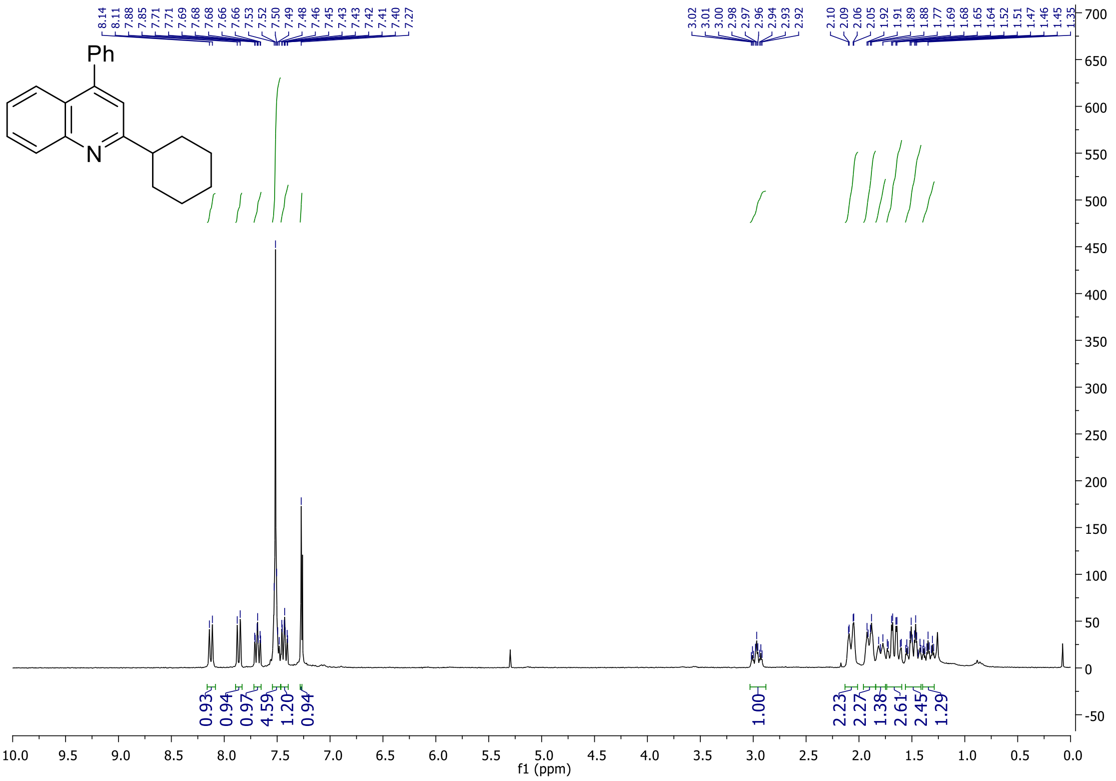

2-(2-Cyclohexylquinolin-4-yl)-1-(4-(trifluoromethyl)phenyl)ethan-1-one (3n)

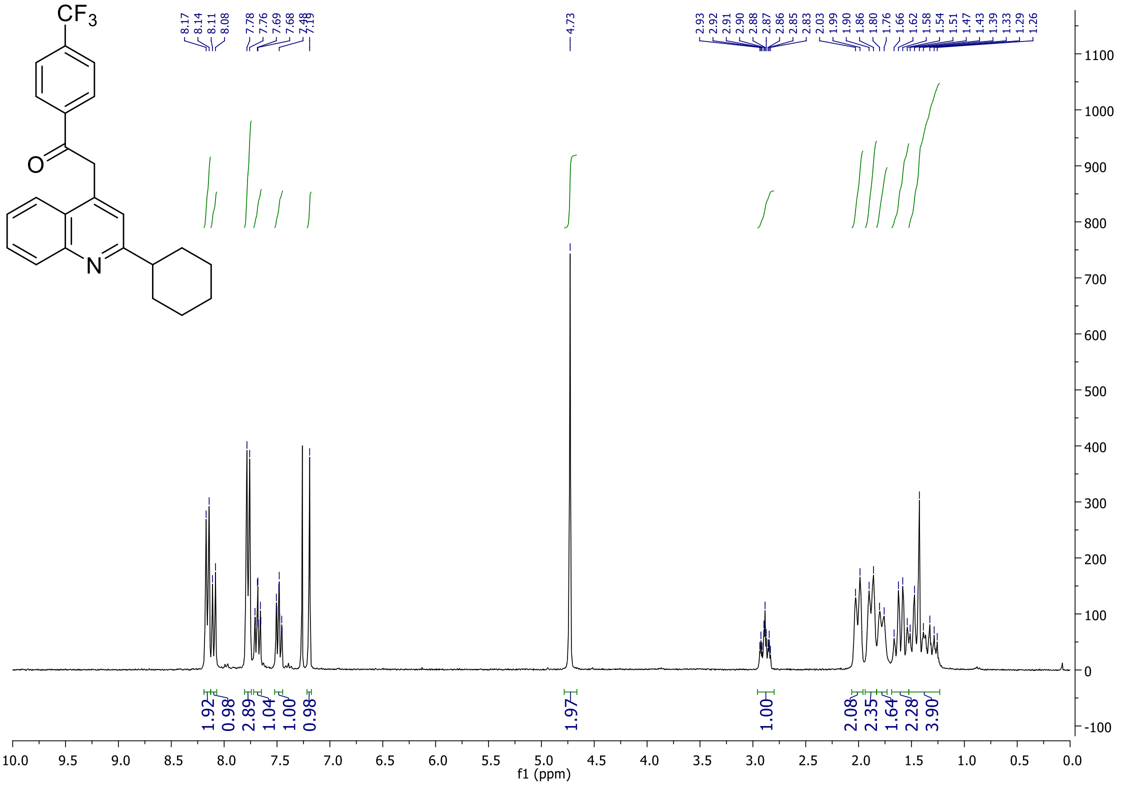

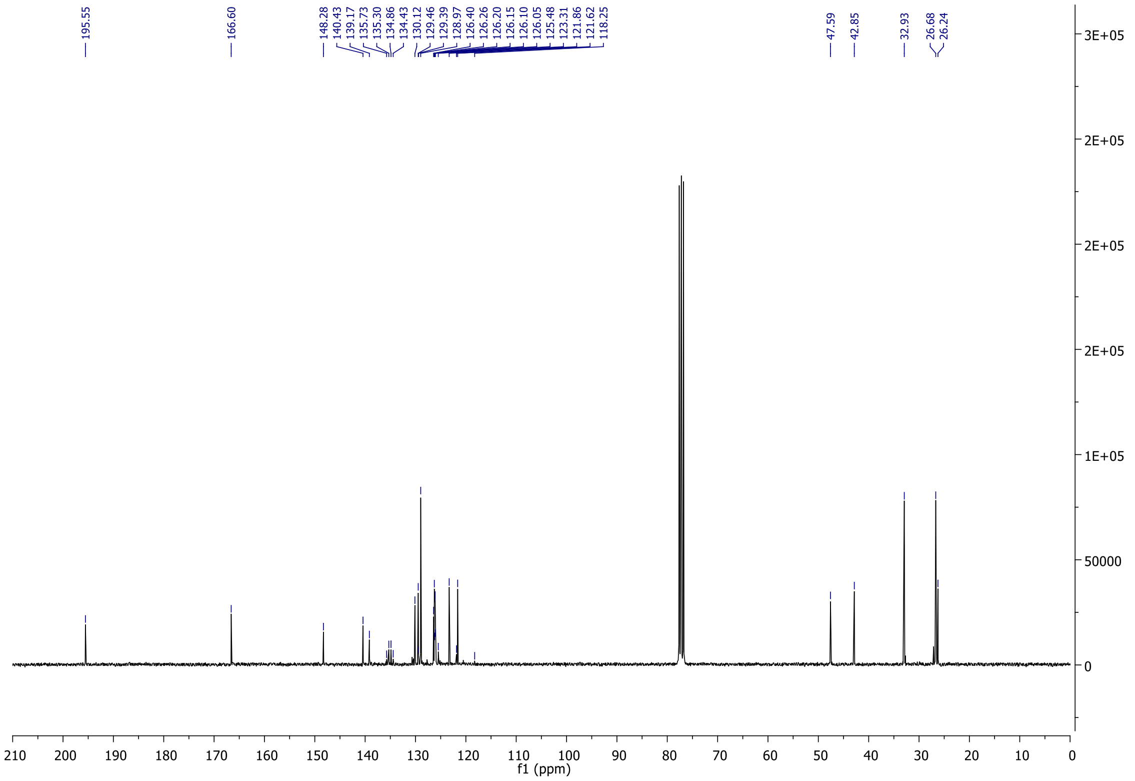

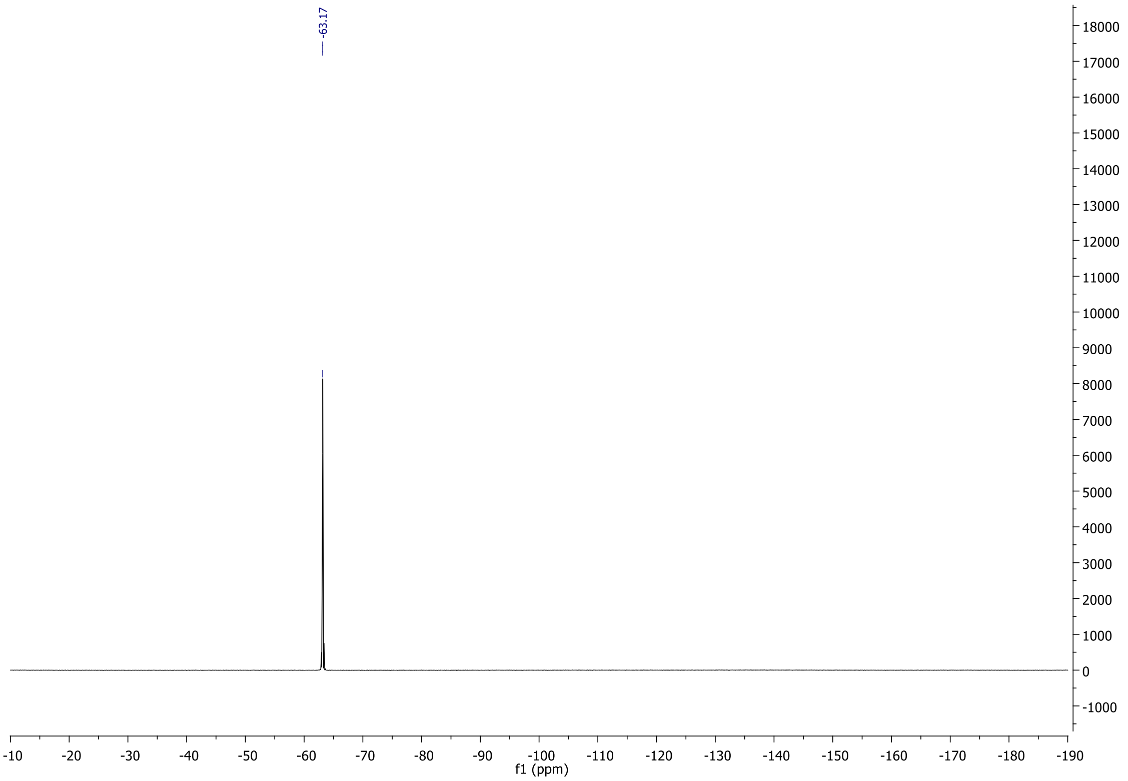

4-Bromo-2-cyclohexylquinoline (3o)

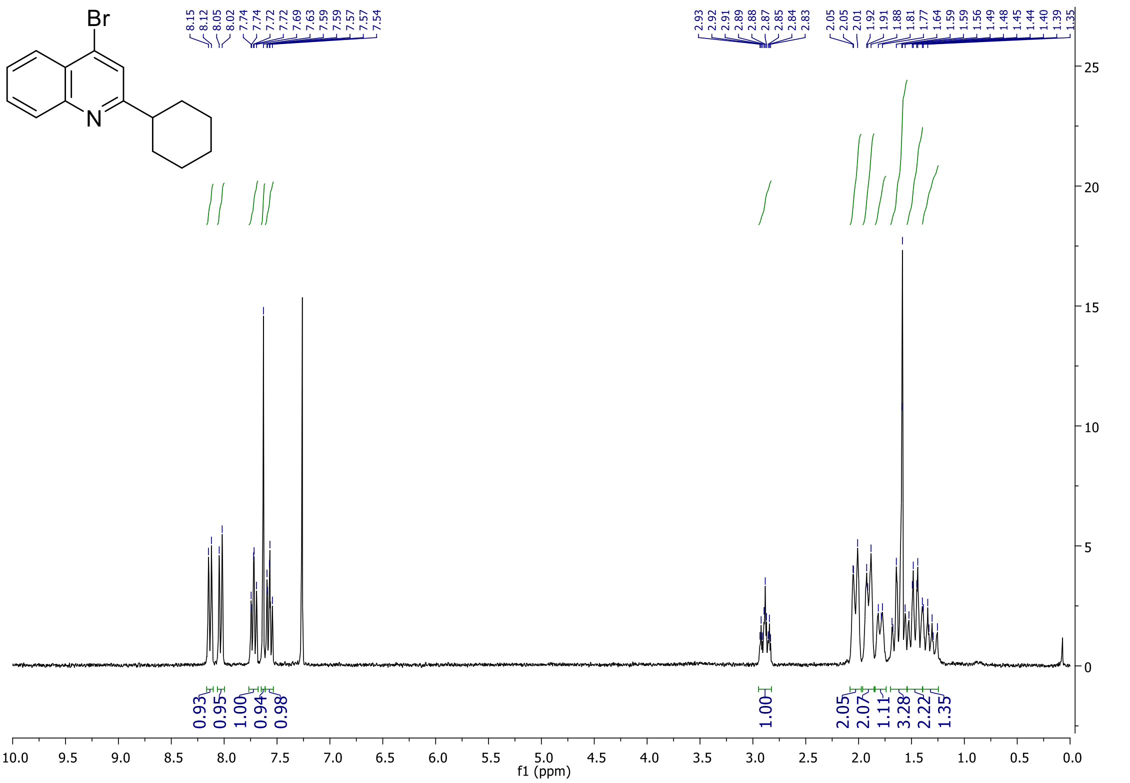

Methyl 6-bromo-2-cyclohexylquinoline-4-carboxylate (3p)

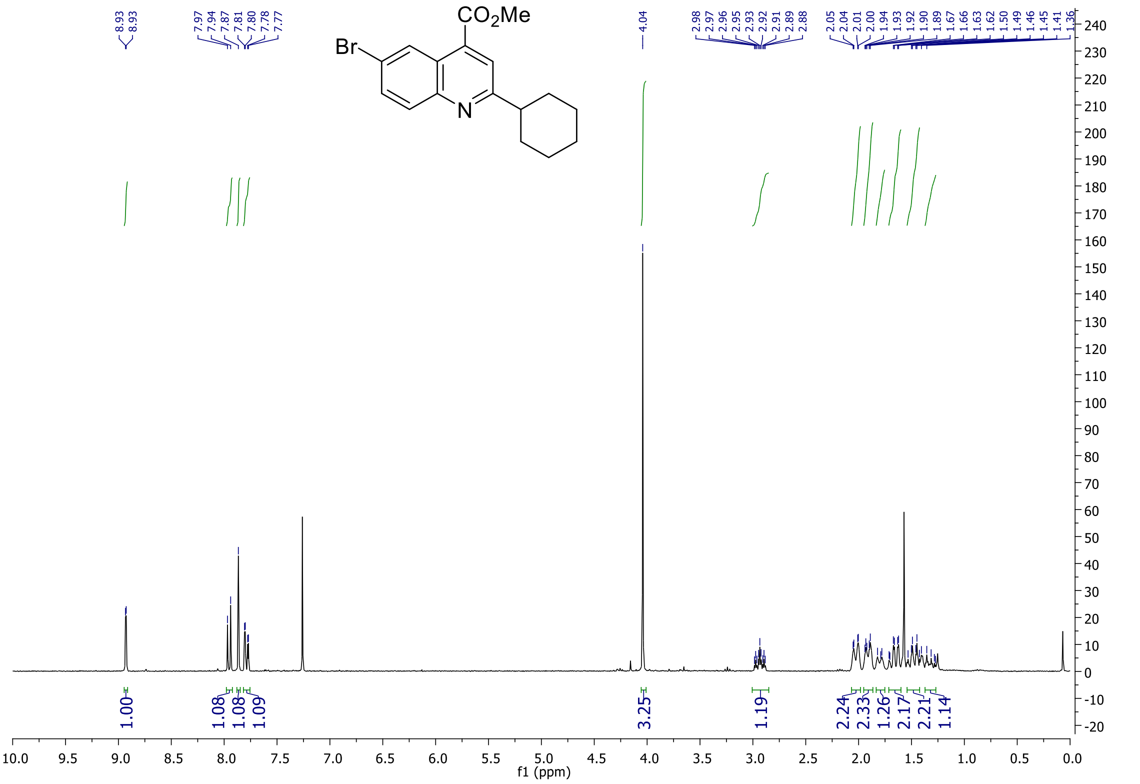

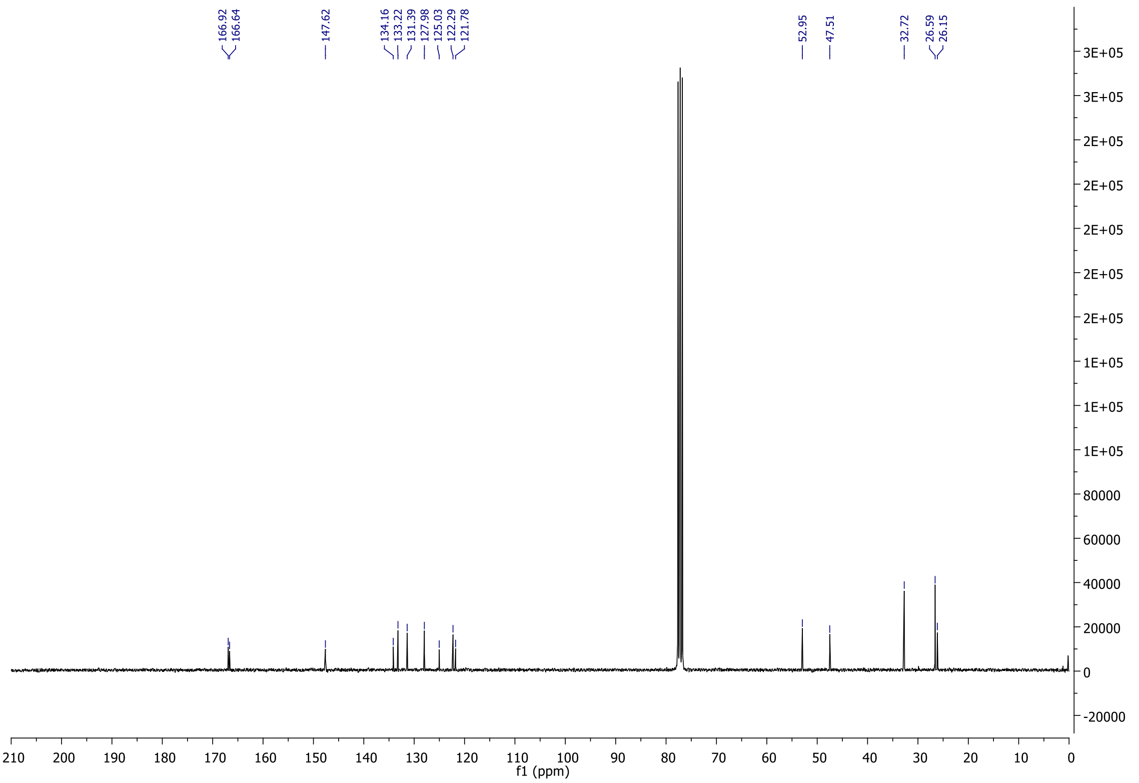

2-Cyclohexyl-N-methylquinoline-4-carboxamide (3q)

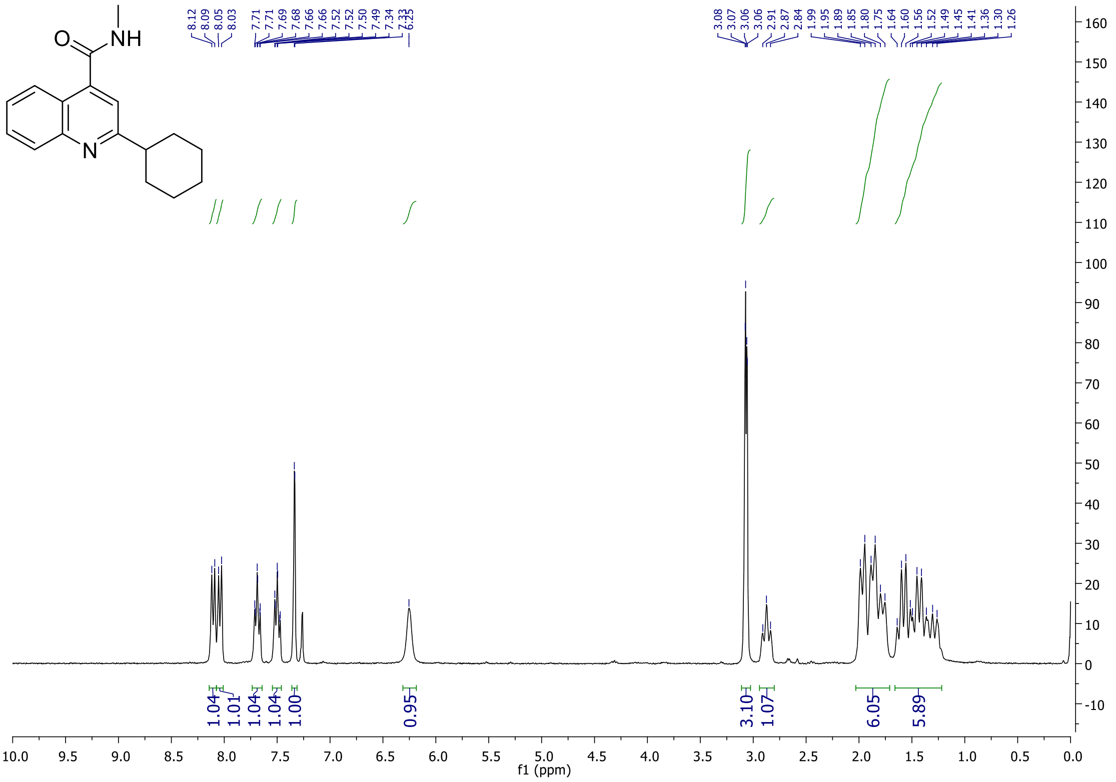

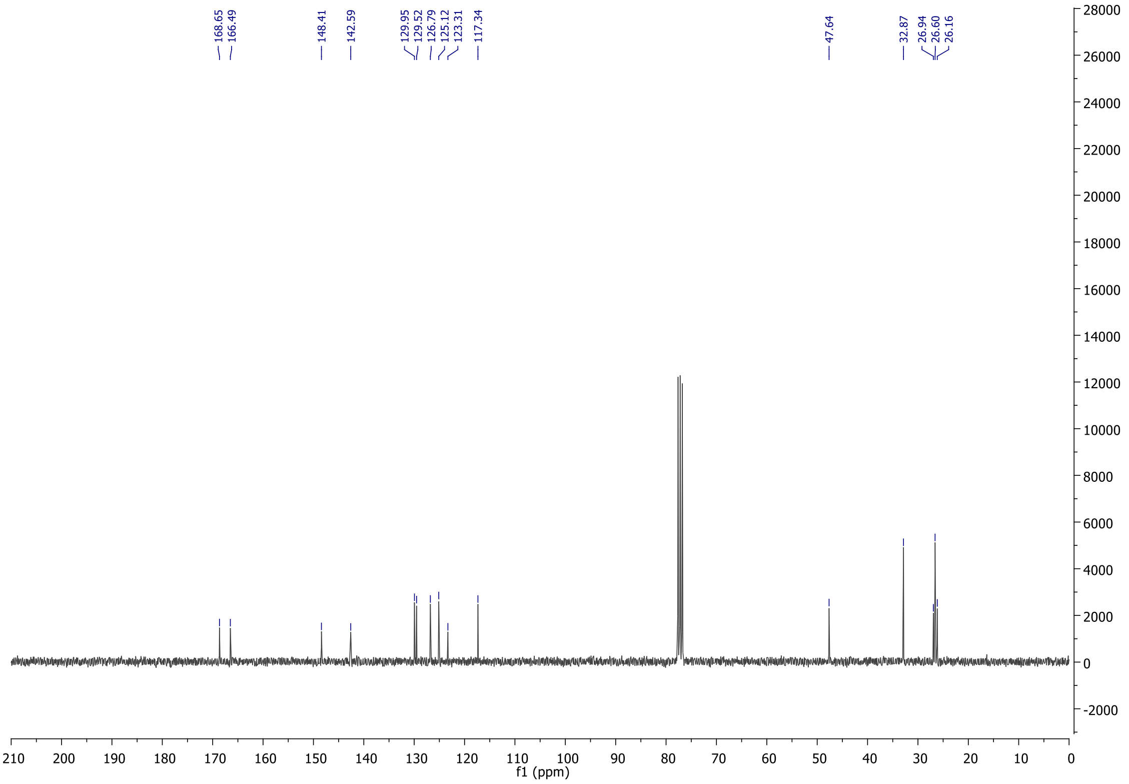

4-Cyclohexyl-2-methylquinoline (3r)

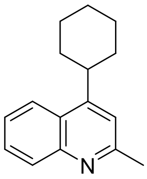

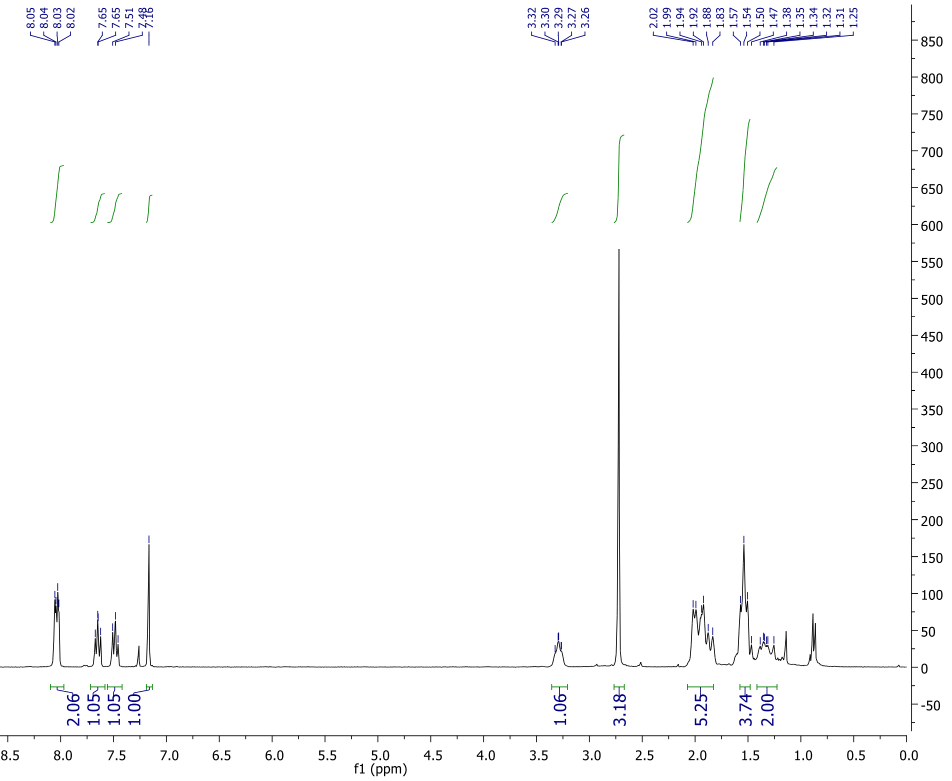

6-Chloro-4-cyclohexyl-2-methylquinoline (3s)

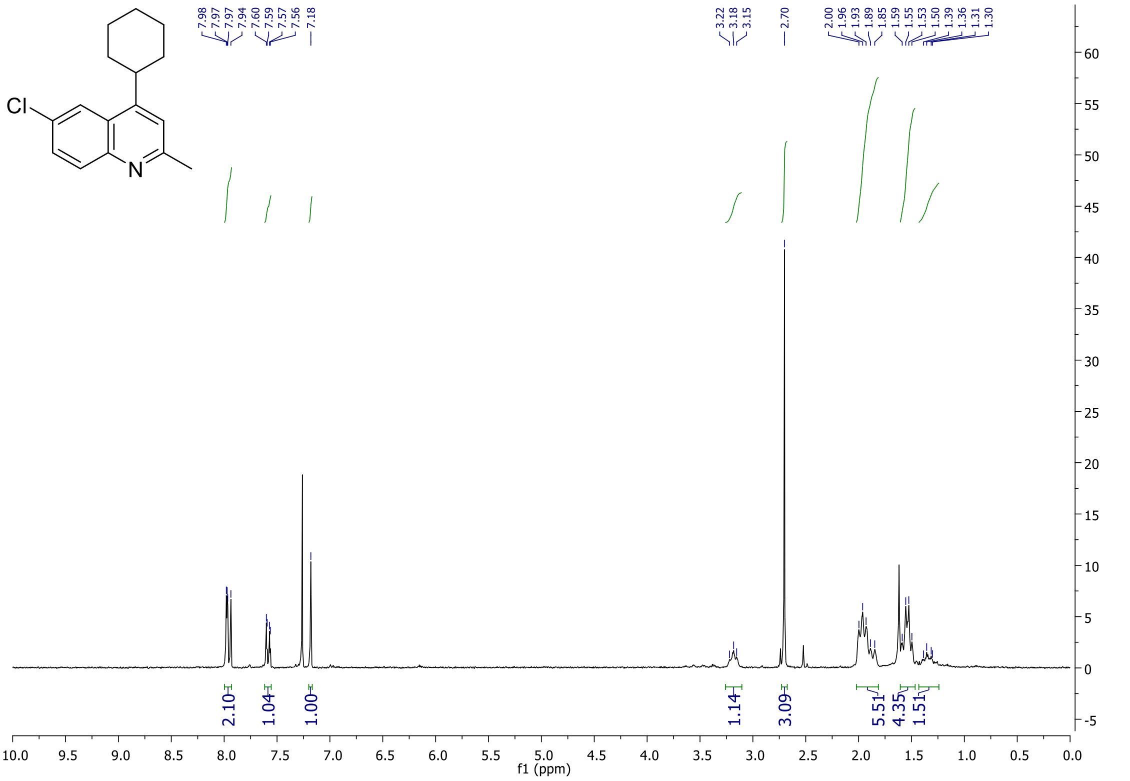

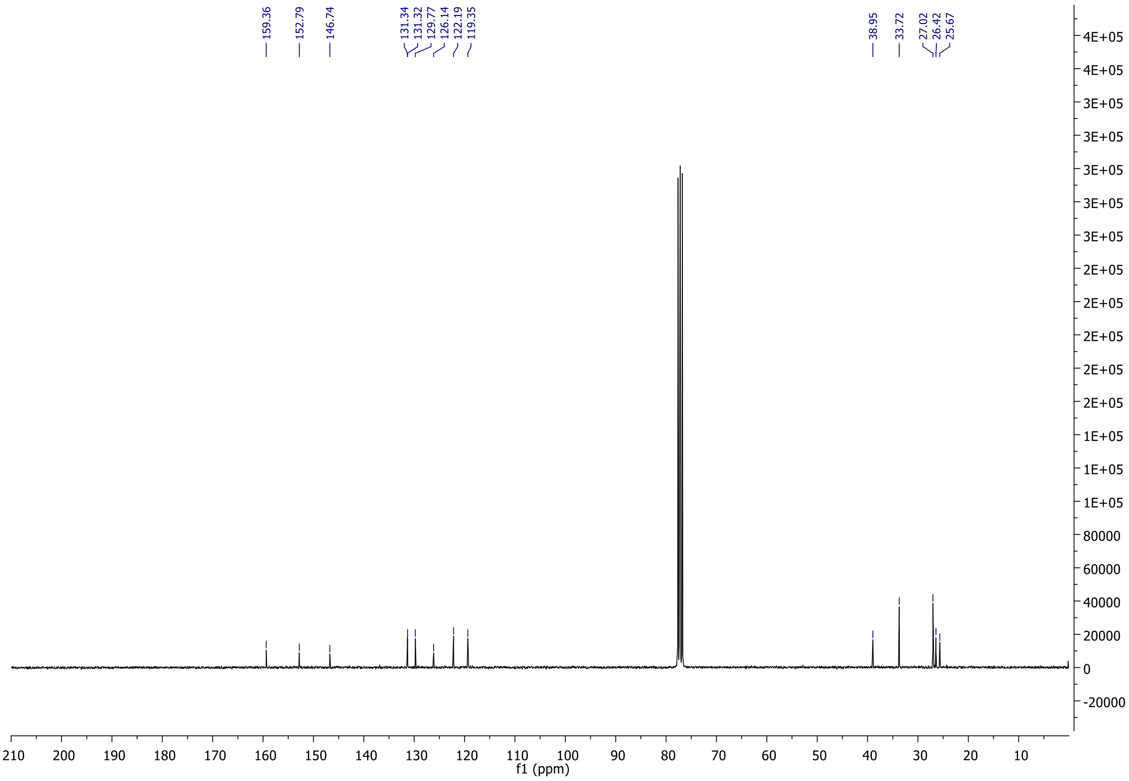

Methyl 4-cyclohexyl-2-methylquinoline-6-carboxylate (3t)

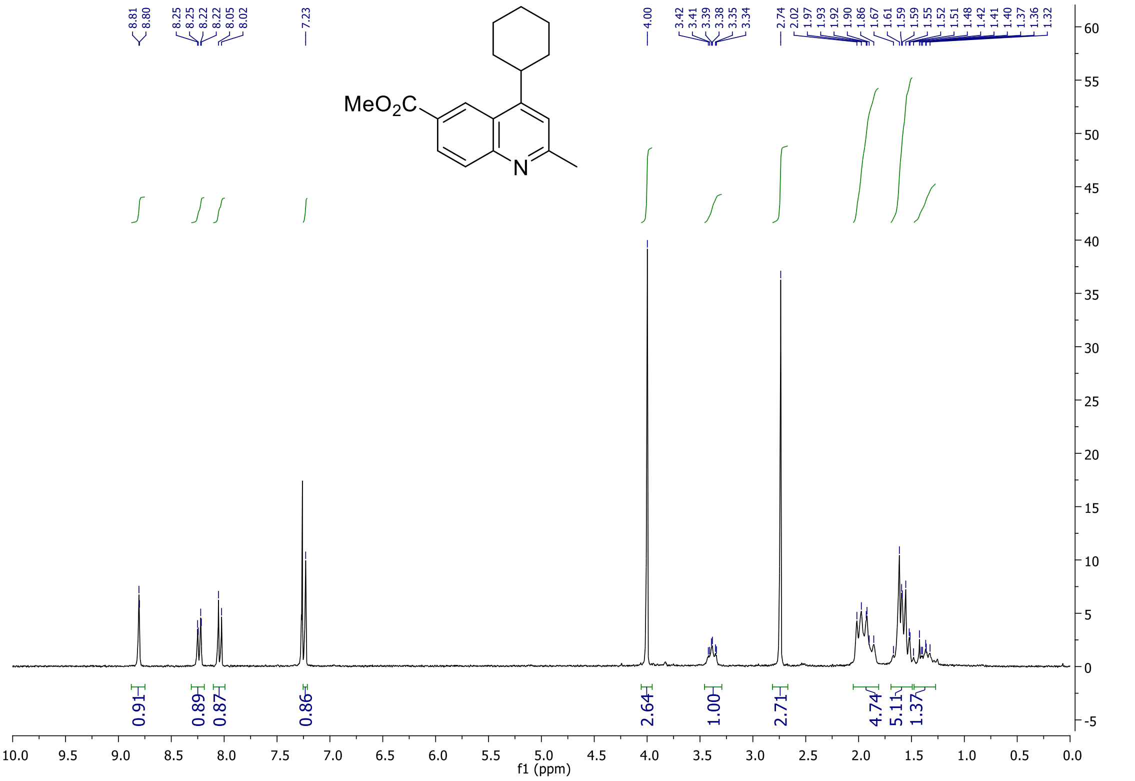

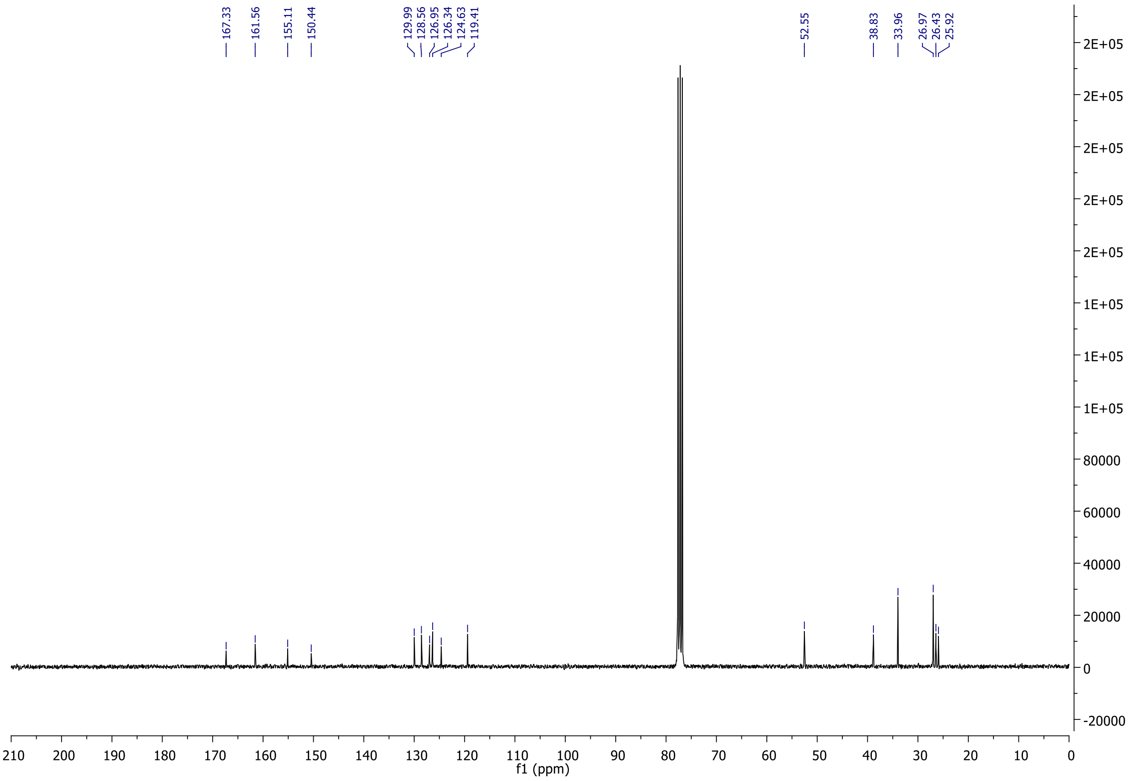

2-Cyclohexylquinoline (3u)

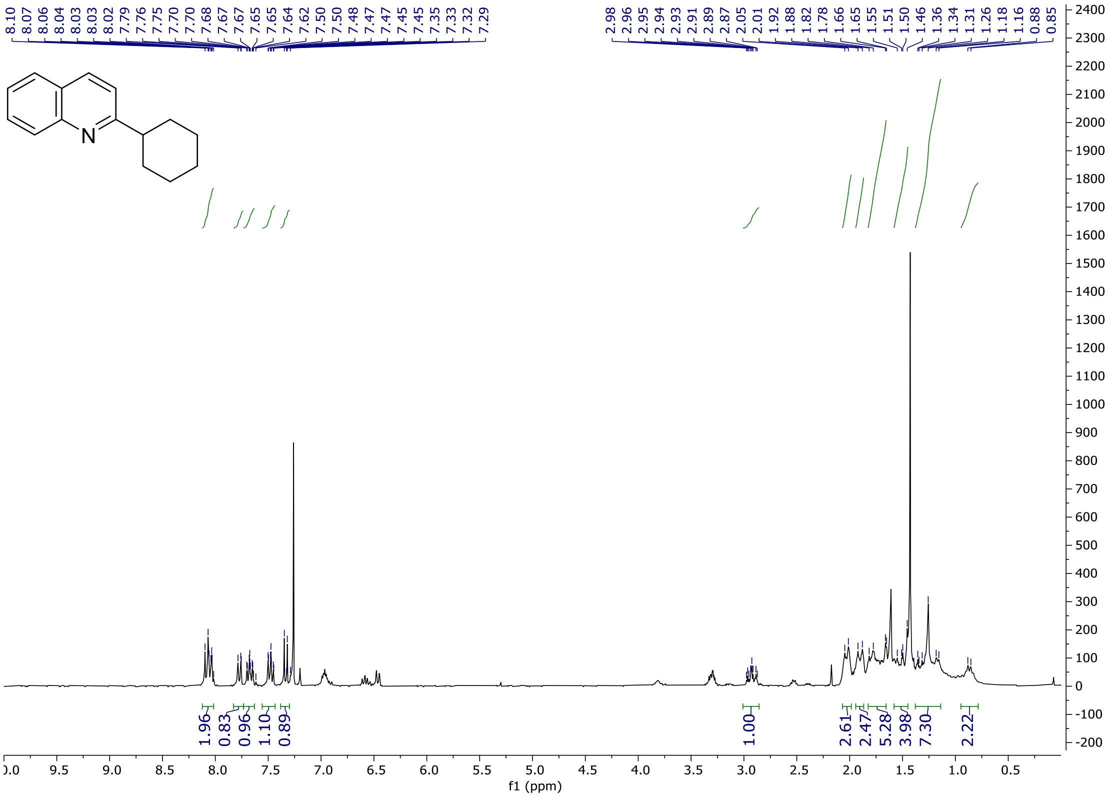

4-Cyclohexylquinoline (3u’)

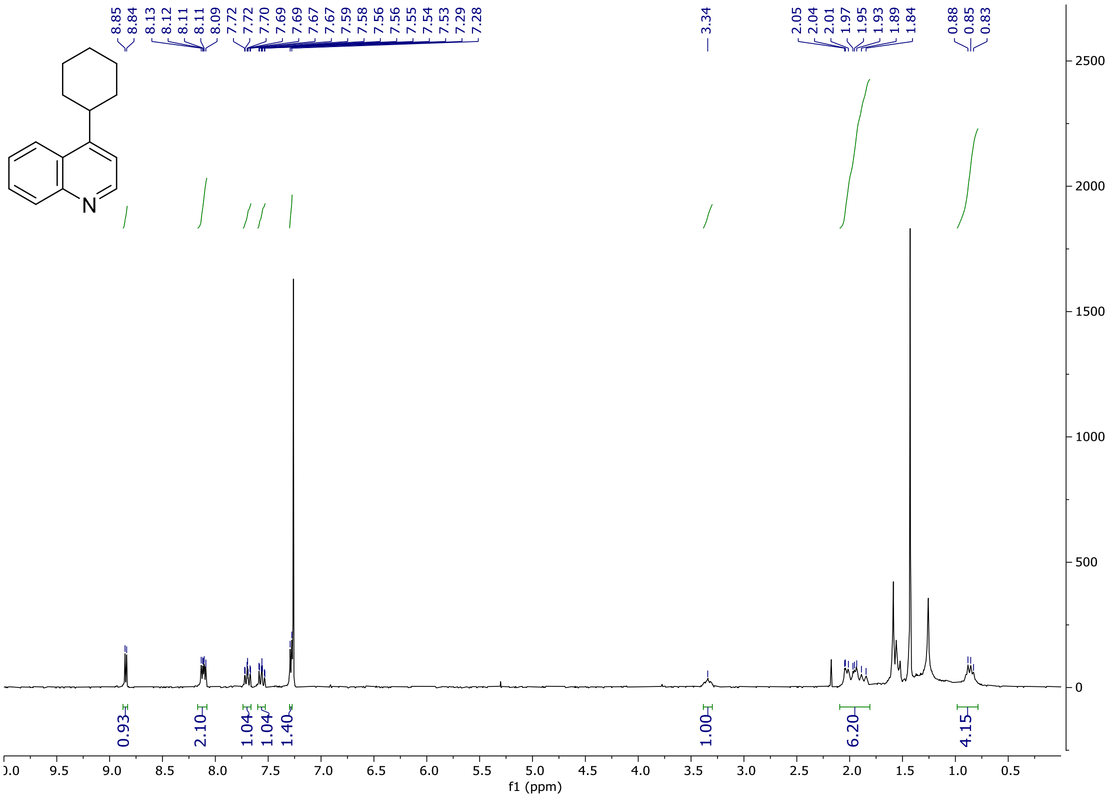

1-Cyclohexyl-3-methylisoquinolin-4-ol (3v)

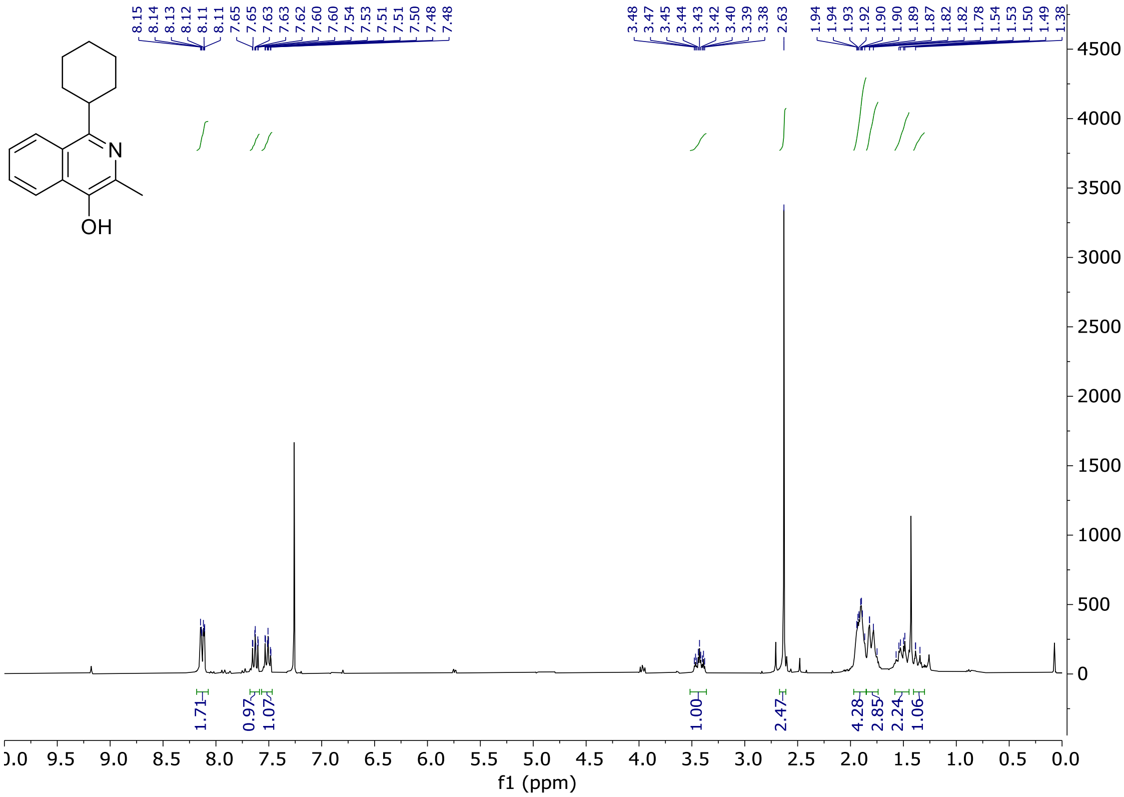

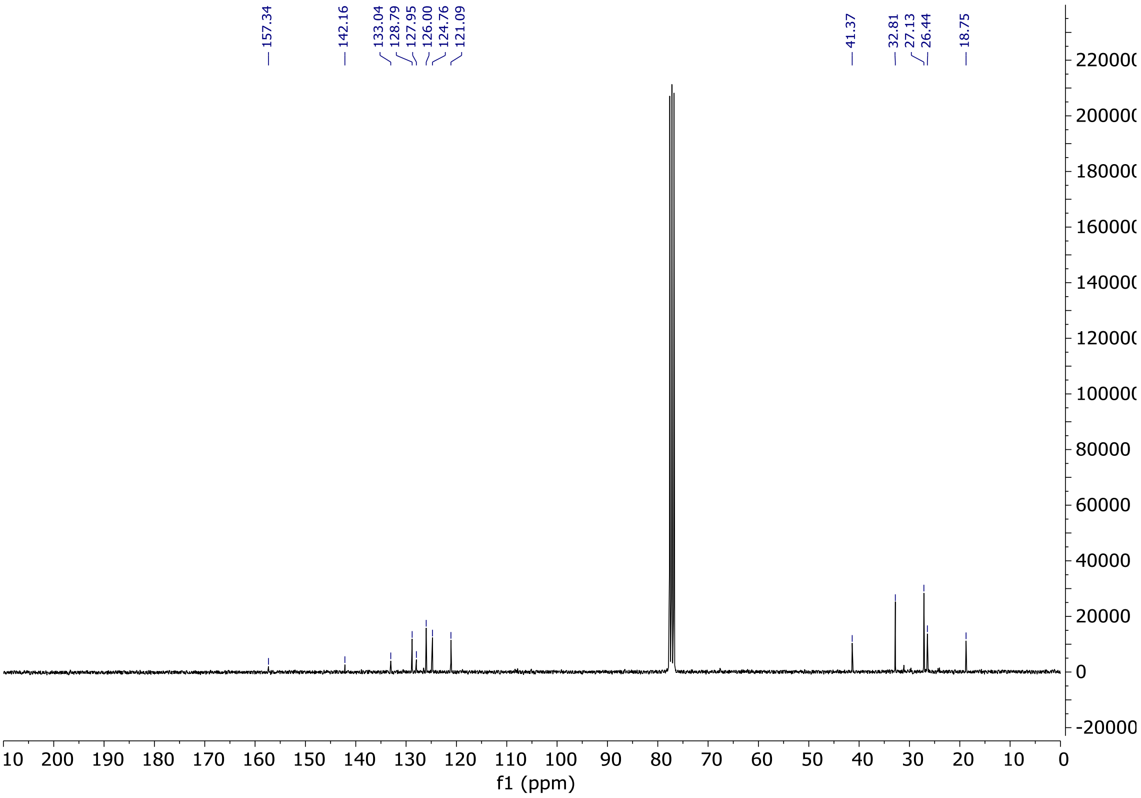

2-Cyclohexyl-4,7-diphenyl-1,10-phenanthroline (3w)

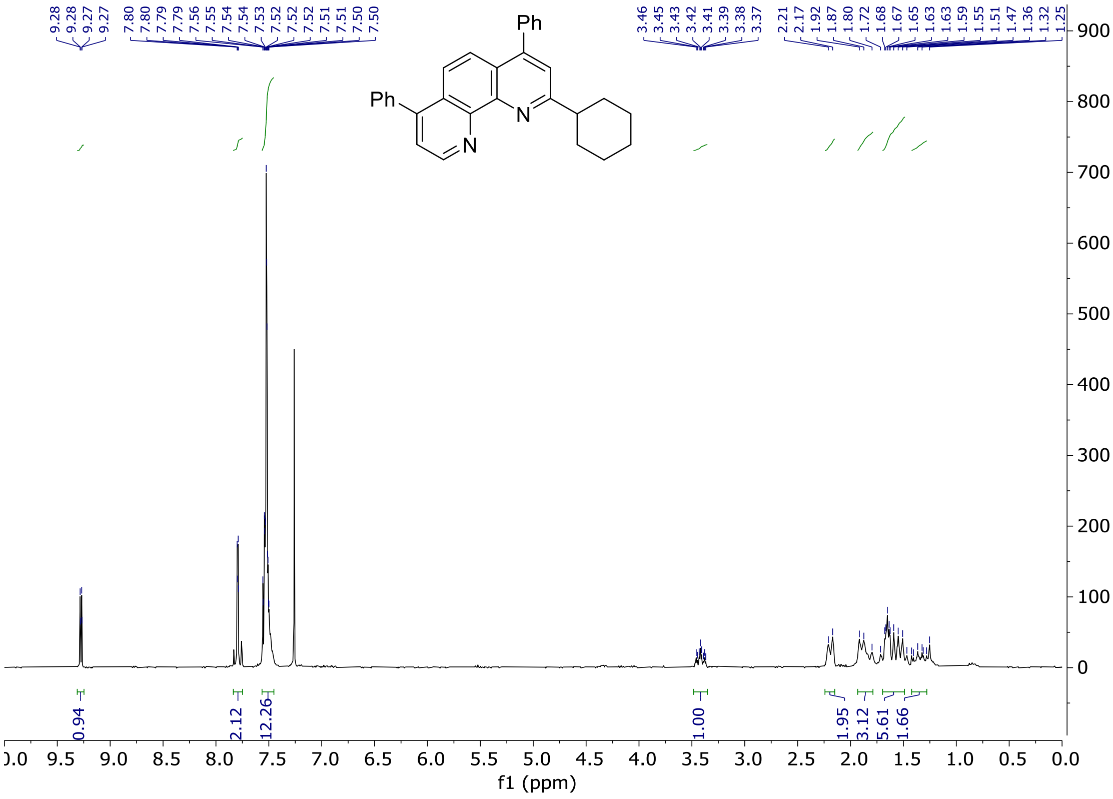

6-Cyclohexylphenanthridine (3x)

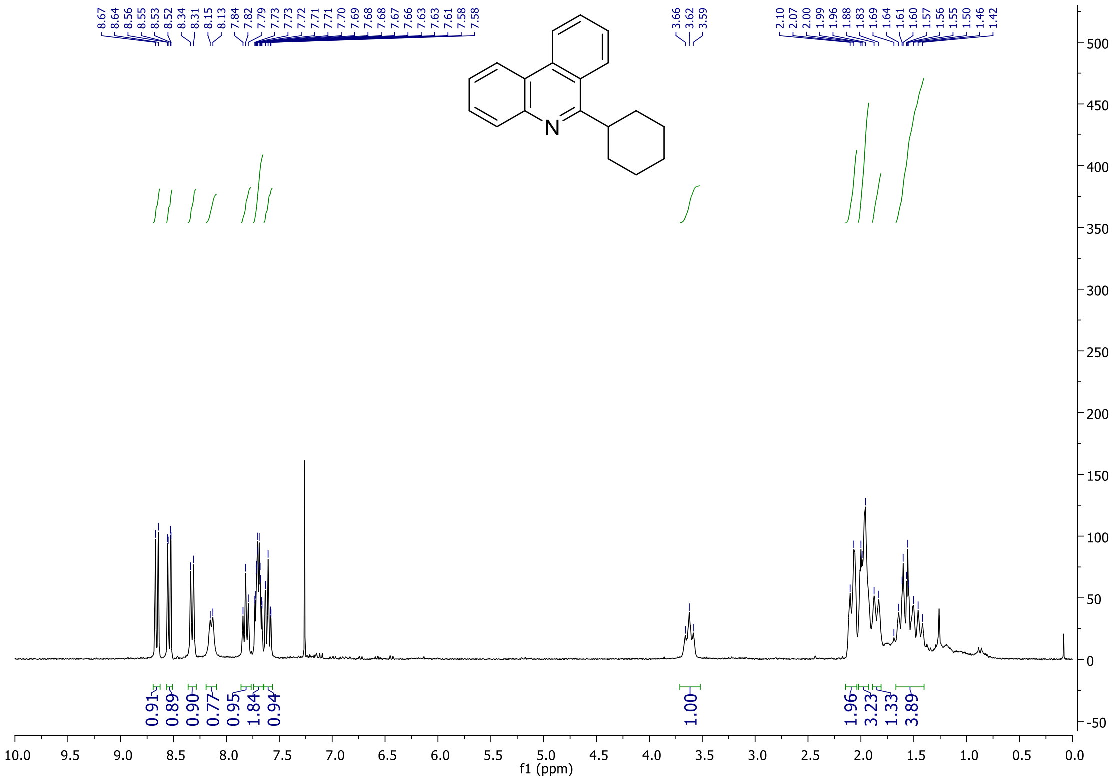

2-Cyclohexylbenzo[d]thiazole (3y)

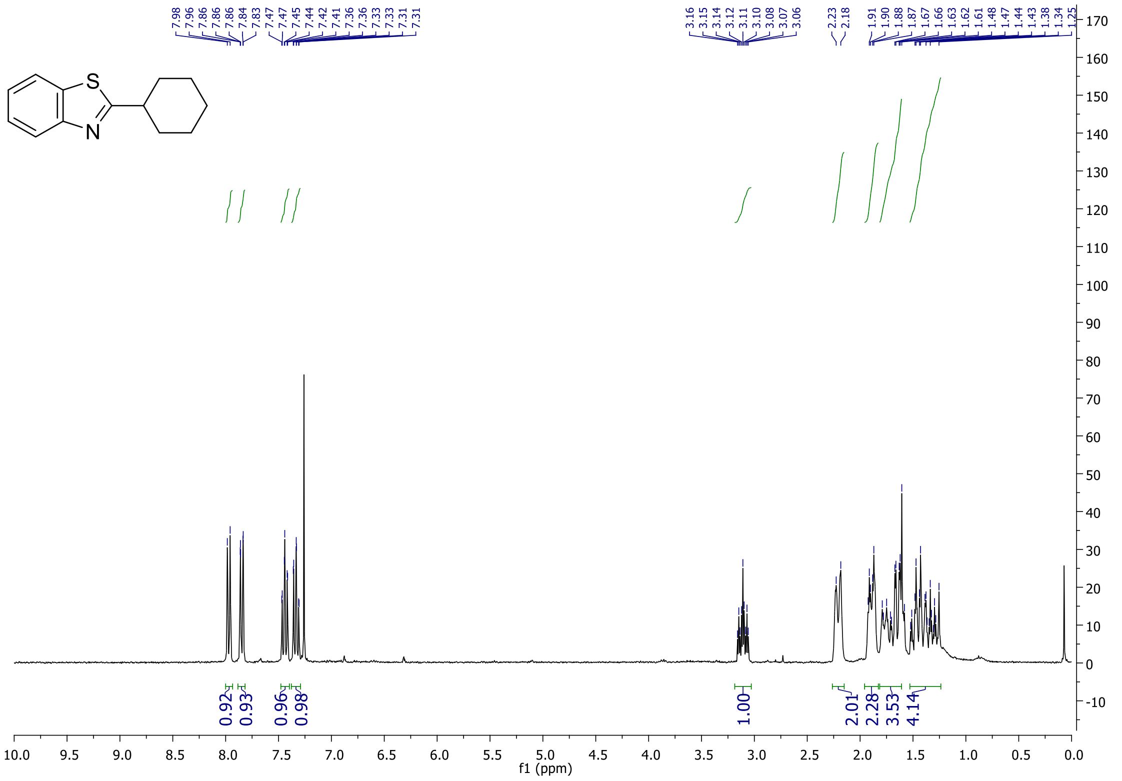

2-Cyclohexyl-1-methyl-1*H*-benzo[*d*]imidazole (3z)

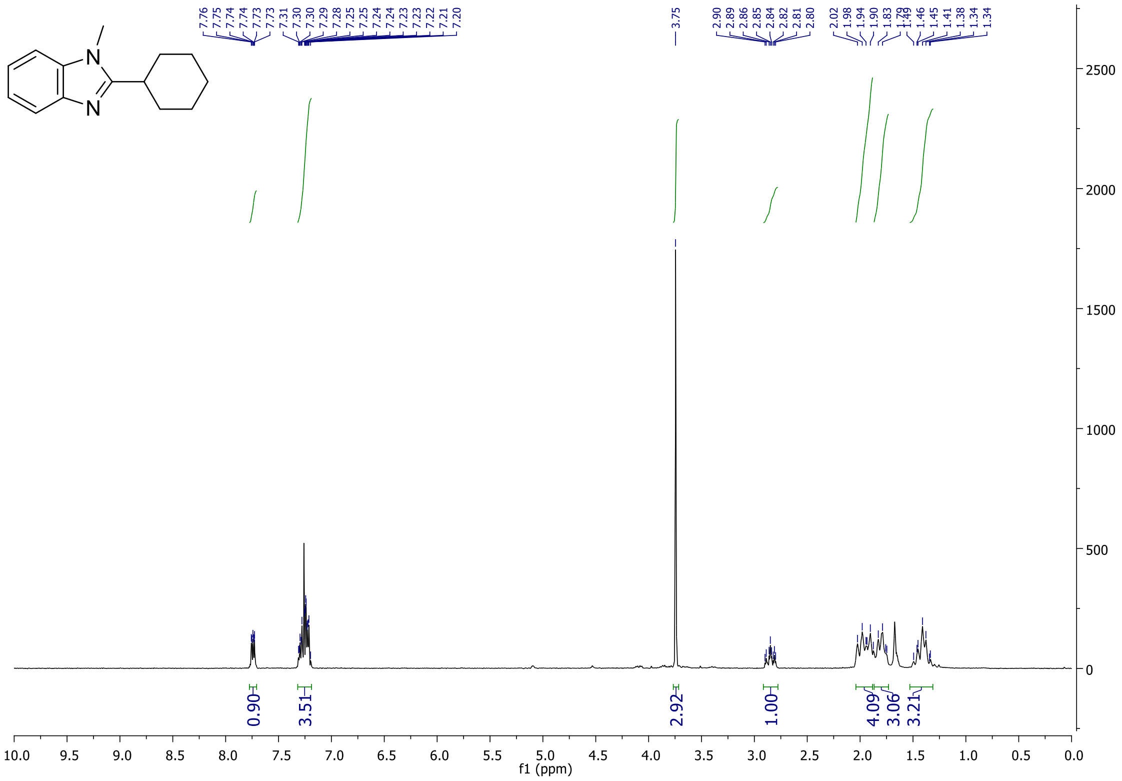

5,7-Dichloro-2-cyclohexyl-4-(4-fluorophenoxy)quinoline (3aa)

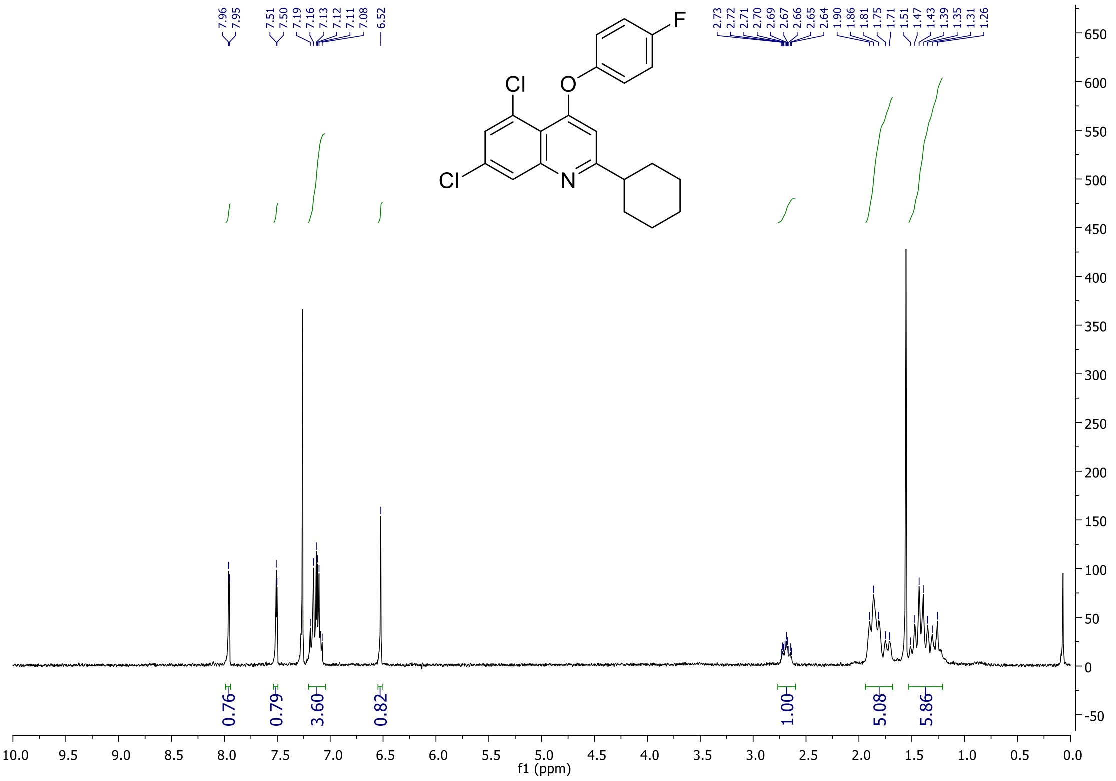

(*R*)-(2-Cyclohexyl-6-methoxyquinolin-4-yl)((1*S*,2*S*,4*S*,5*R*)-5-vinylquinuclidin-2-yl)methanol (3ab)

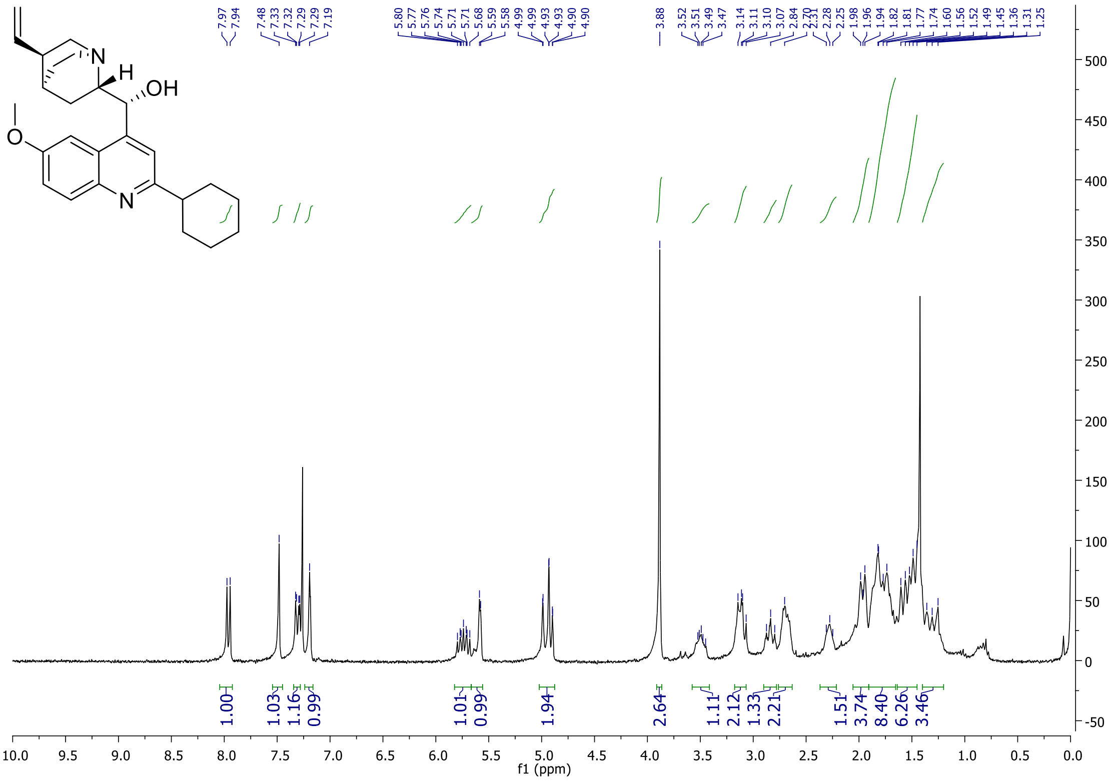

(*R*)-(2-Cyclohexylquinolin-4-yl)((1*S*,2*S*,4*S*,5*R*)-5-vinylquinuclidin-2-yl)methanol (3ac)

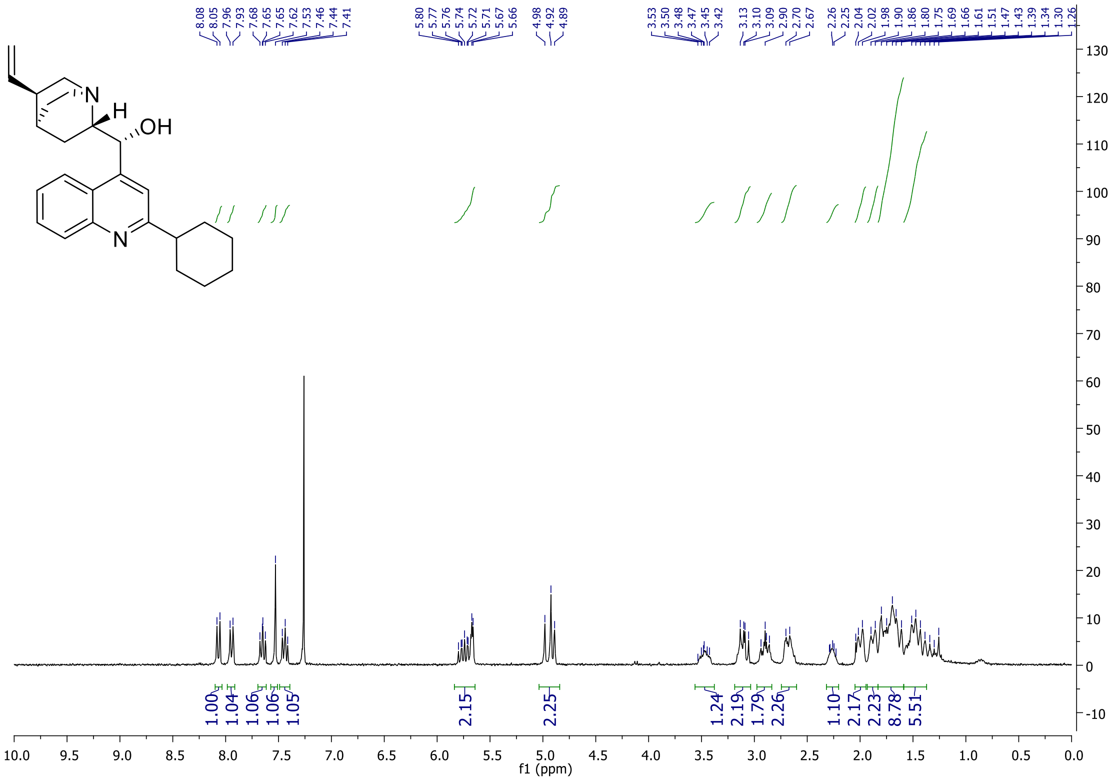

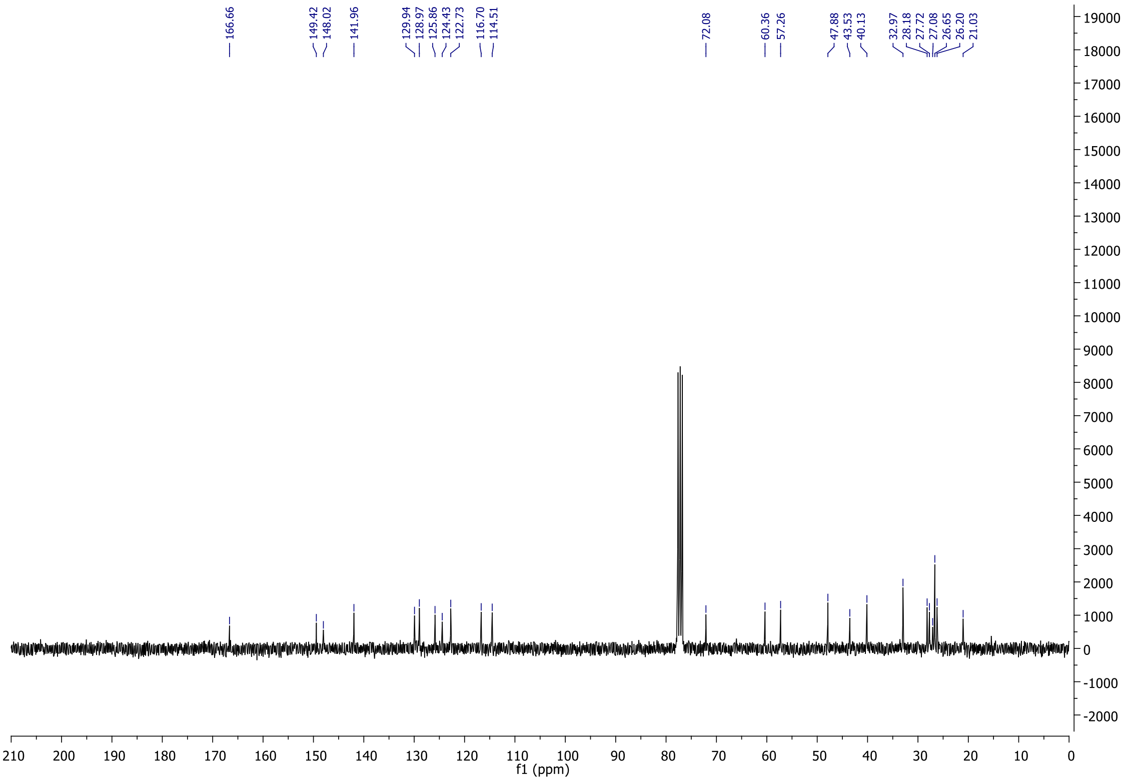

(*S*)-11-Cyclohexyl-4-ethyl-4-hydroxy-1,12-dihydro-14*H*-pyrano[3',4':6,7]indolizino[1,2-*b*] quinoline-3,14(4*H*)-dione (3ad)

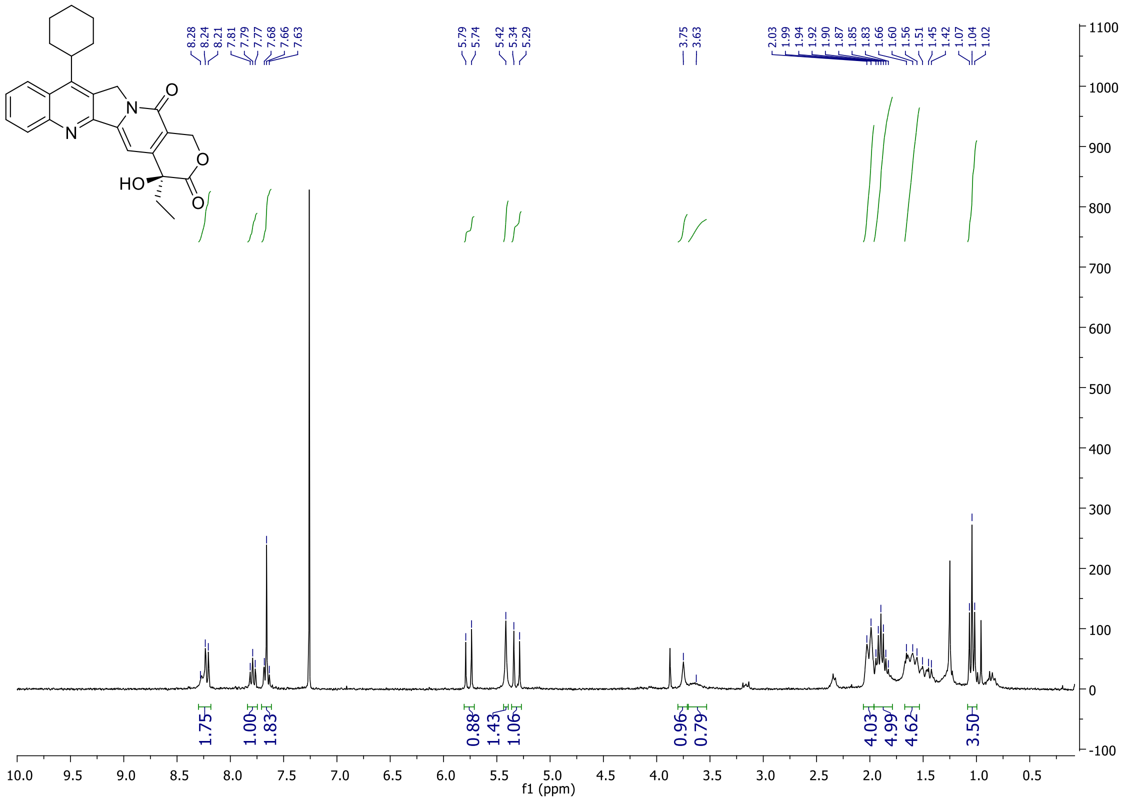

Methyl 6-bromo-2-isopropylquinoline-4-carboxylate (6)

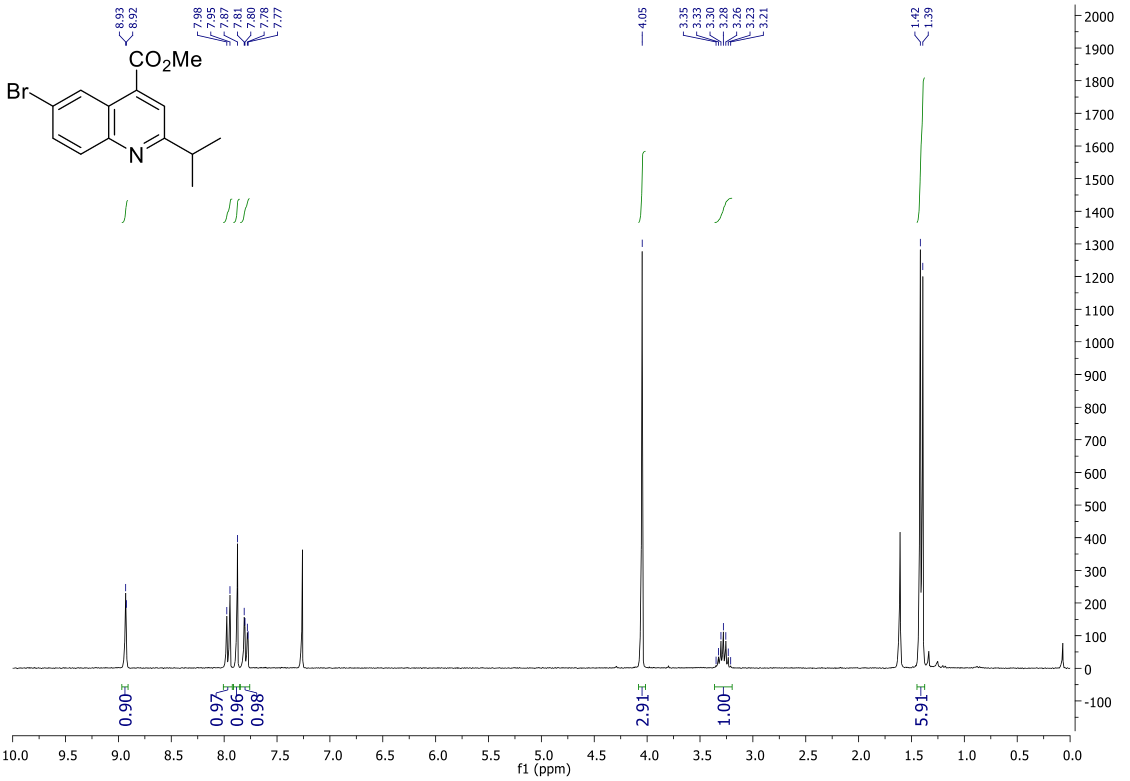

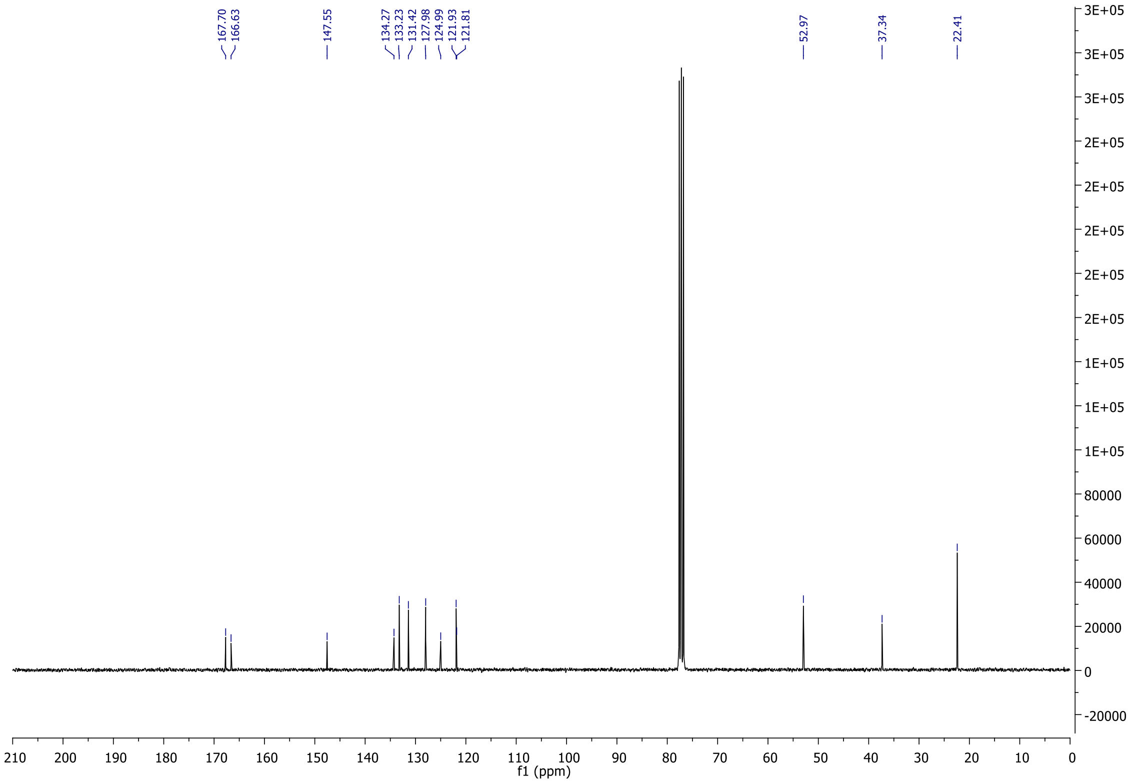

Methyl 2-cyclohexylquinoline-4-carboxylate (8)

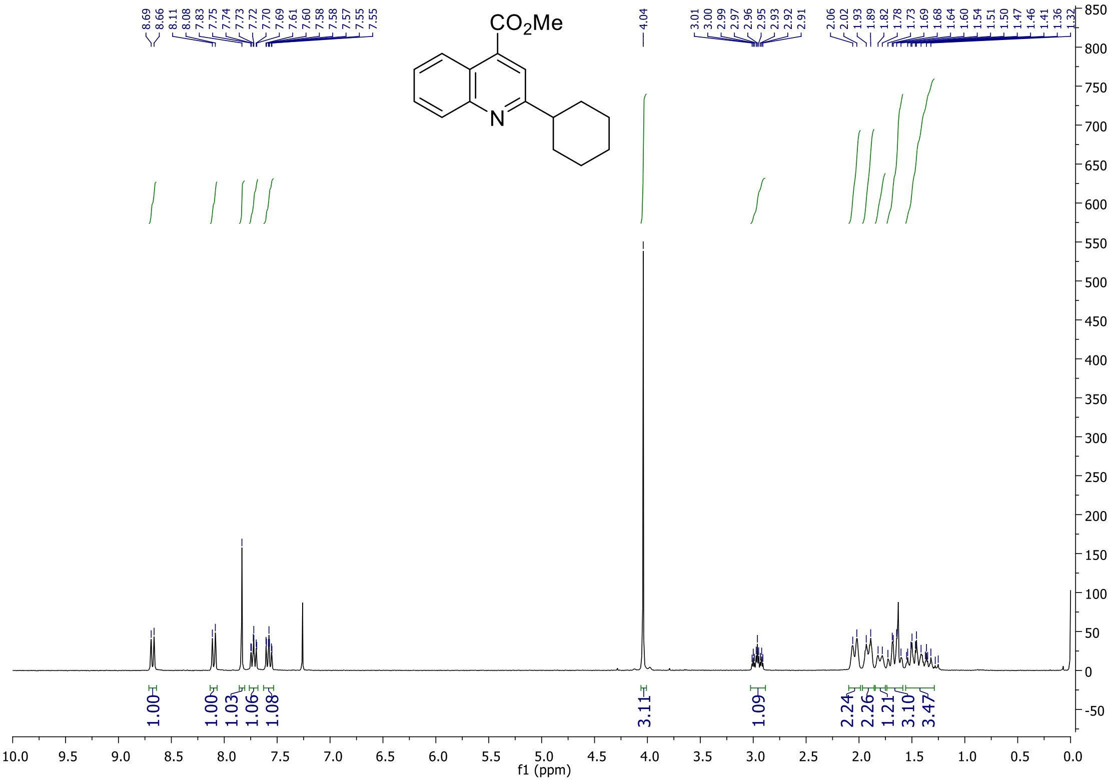

2-Cyclohexylquinoline-4-carboxylic acid (9)

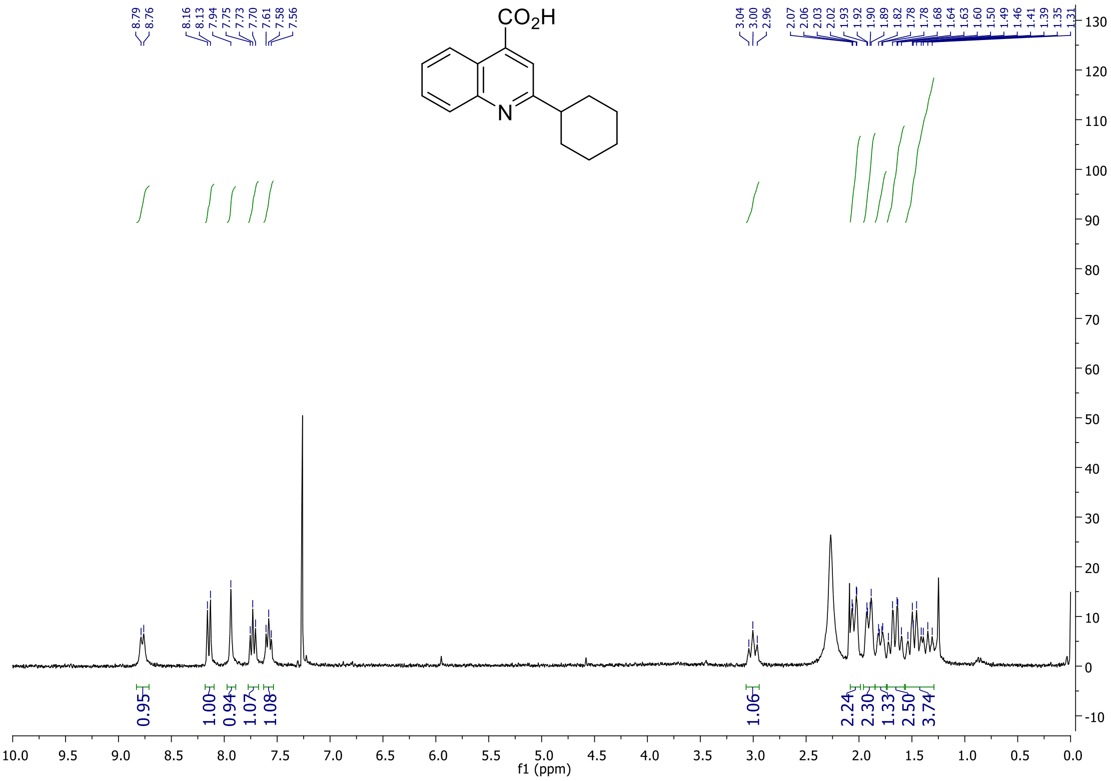

3-Cyclohexyl-*N*,2-diphenylpropanamide (11)

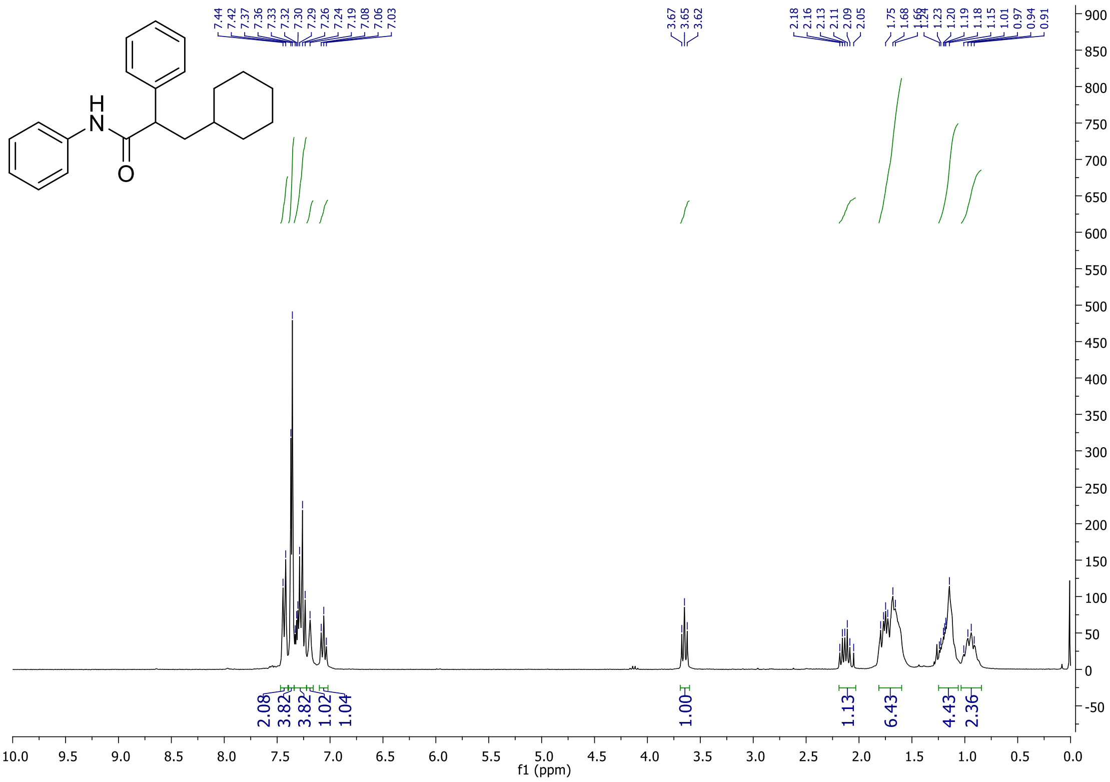

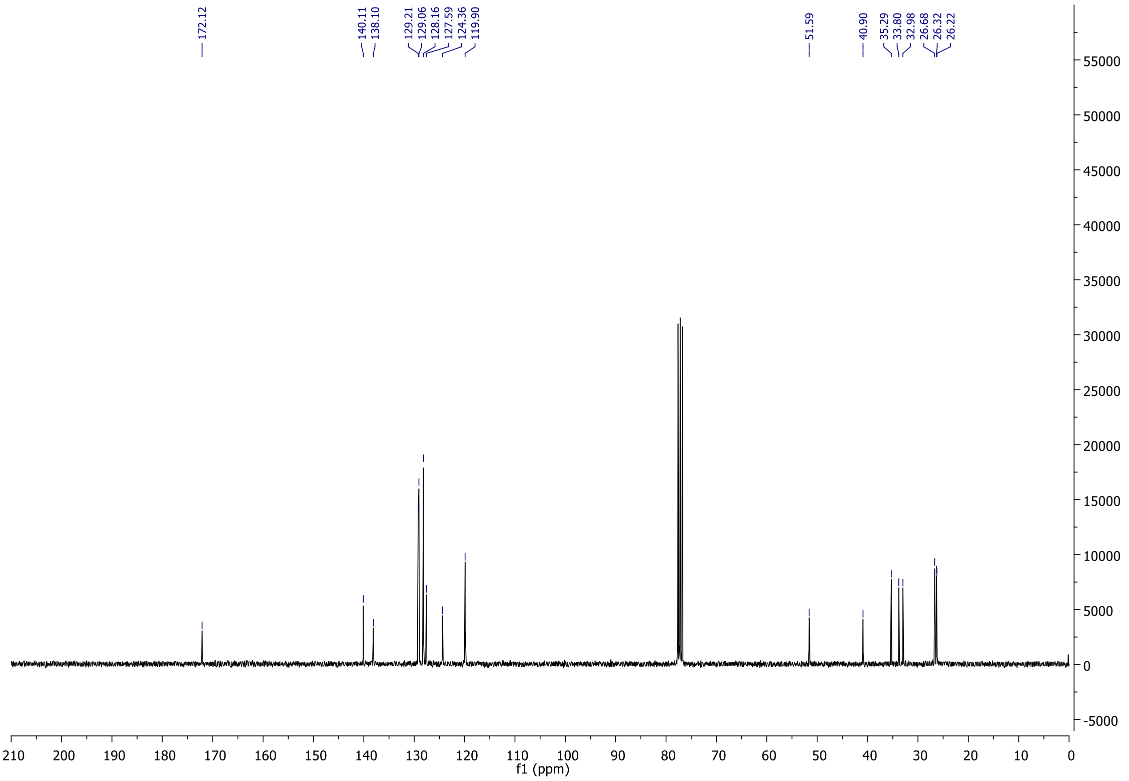

9-(*tert*-Butyl)-9,10-dihydroacridine (4a)

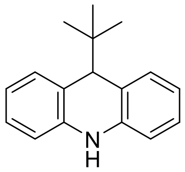

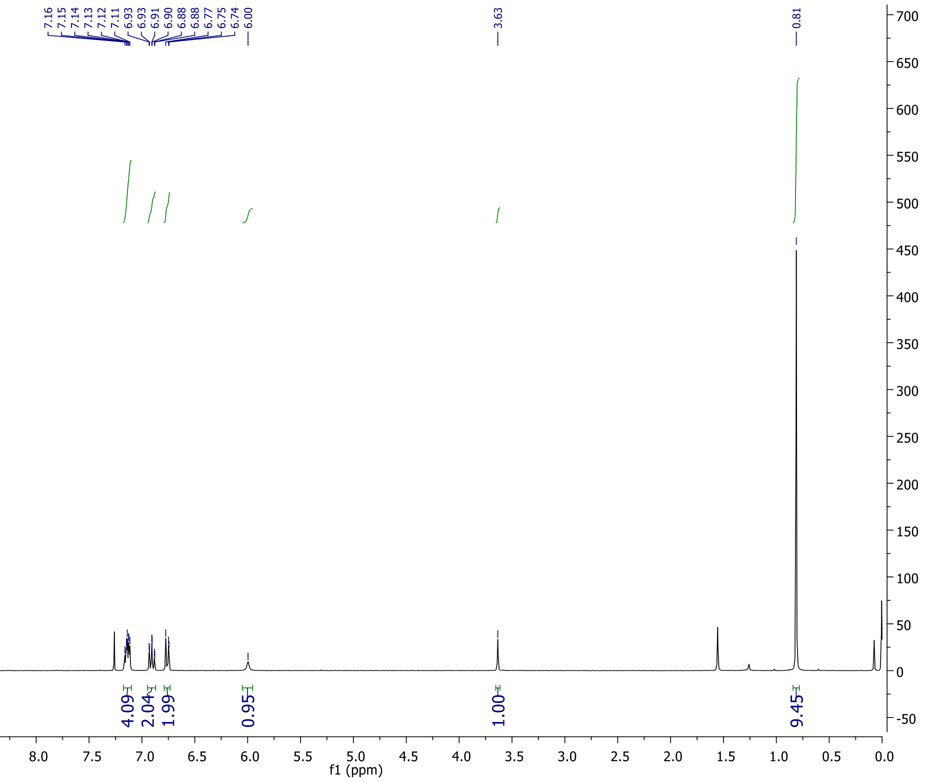

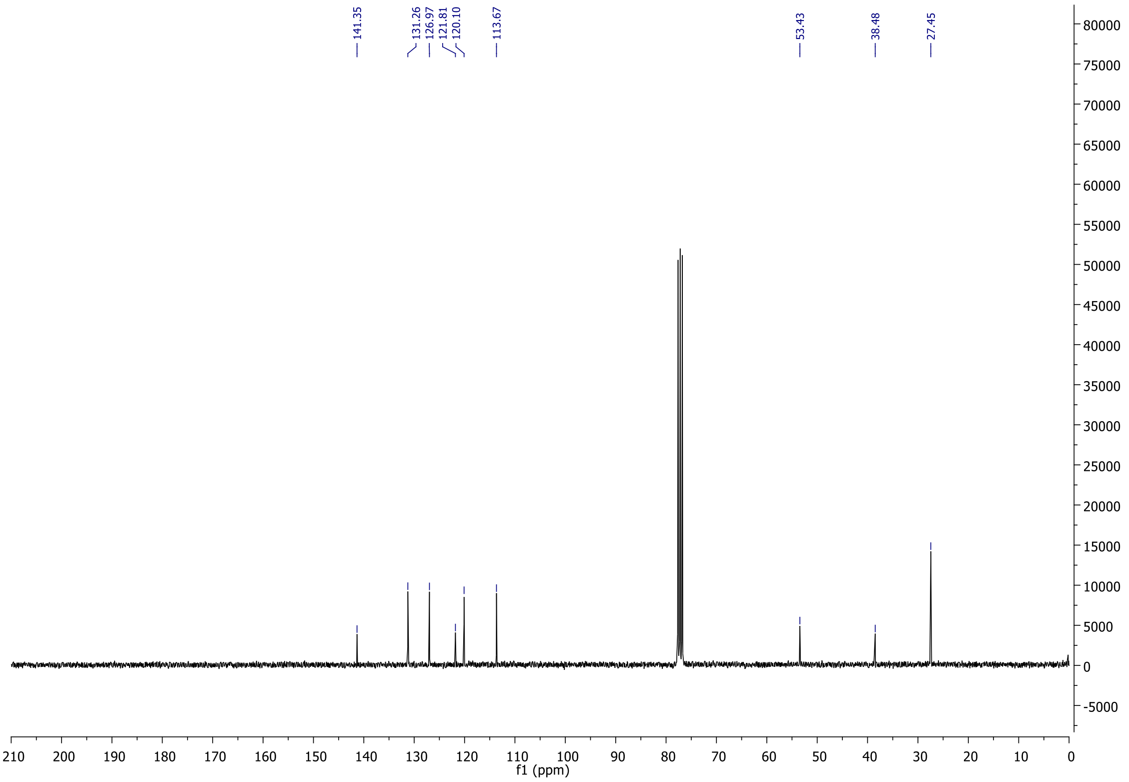

9-Allyl-9,10-dihydroacridine (4b)

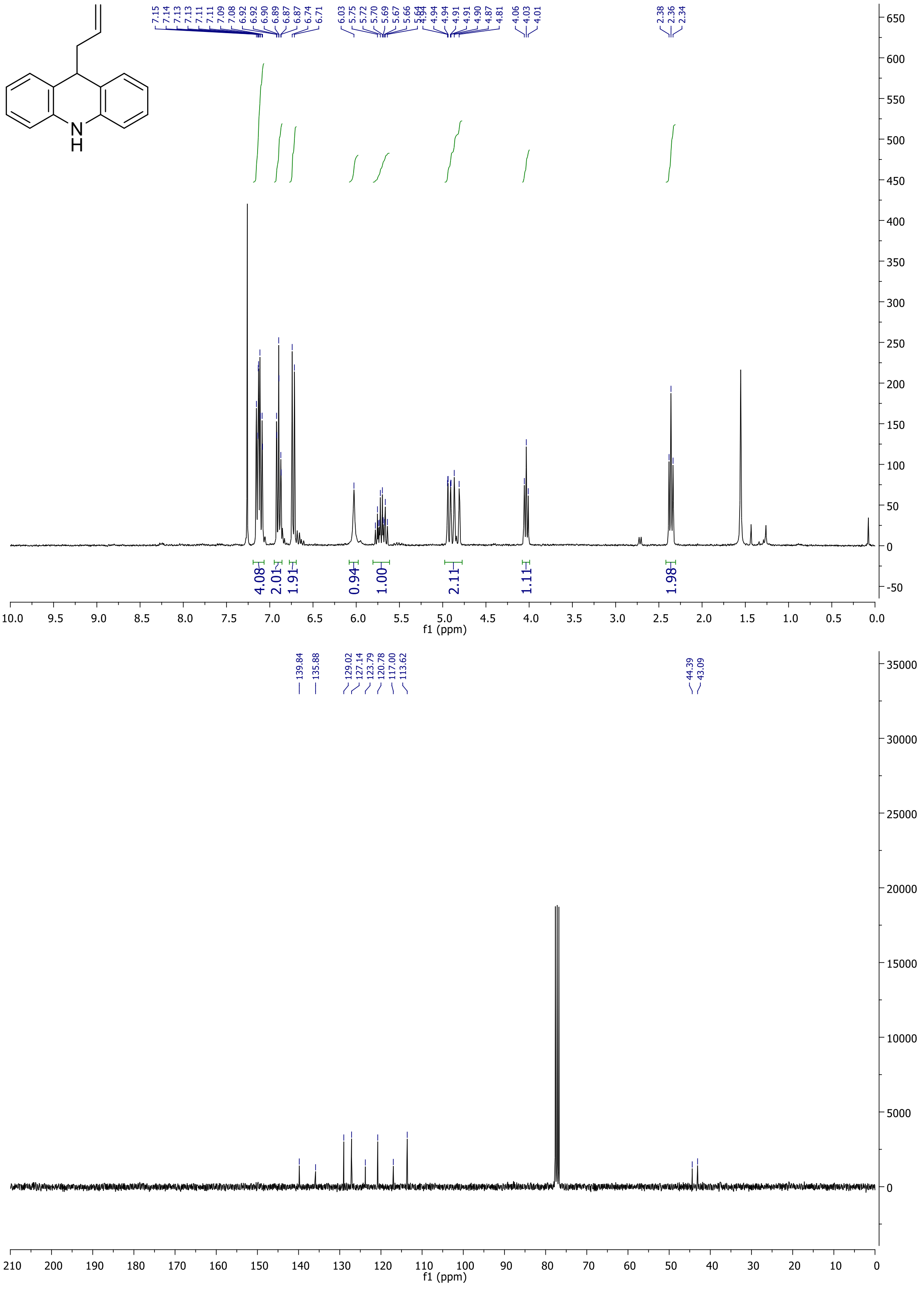

9-Benzyl-9,10-dihydroacridine (4c)

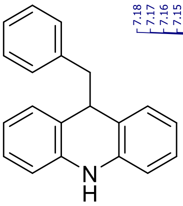

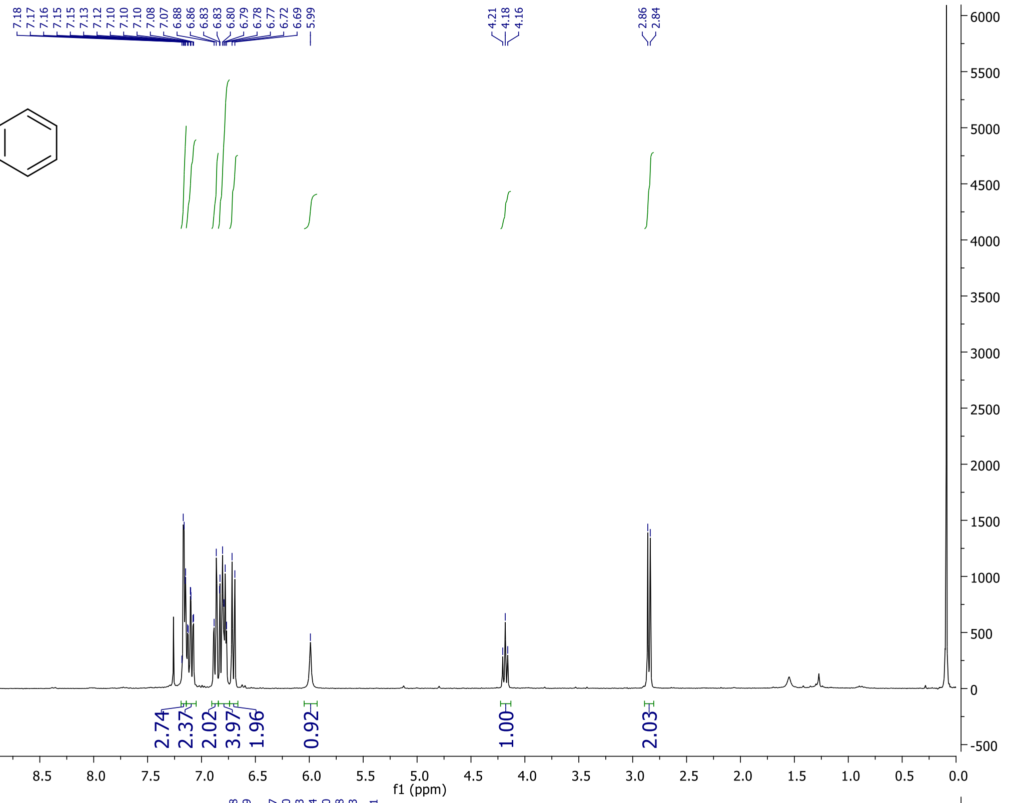

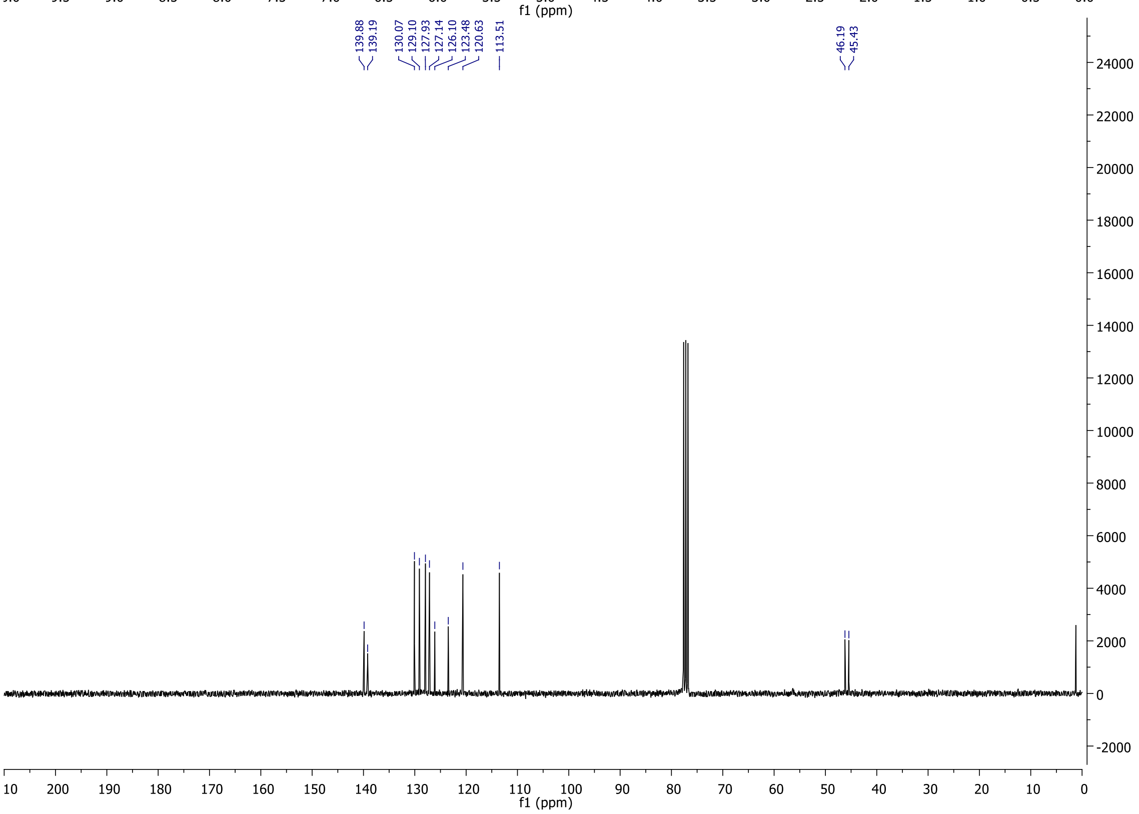

9-(4-Chlorobenzyl)-9,10-dihydroacridine (4d)

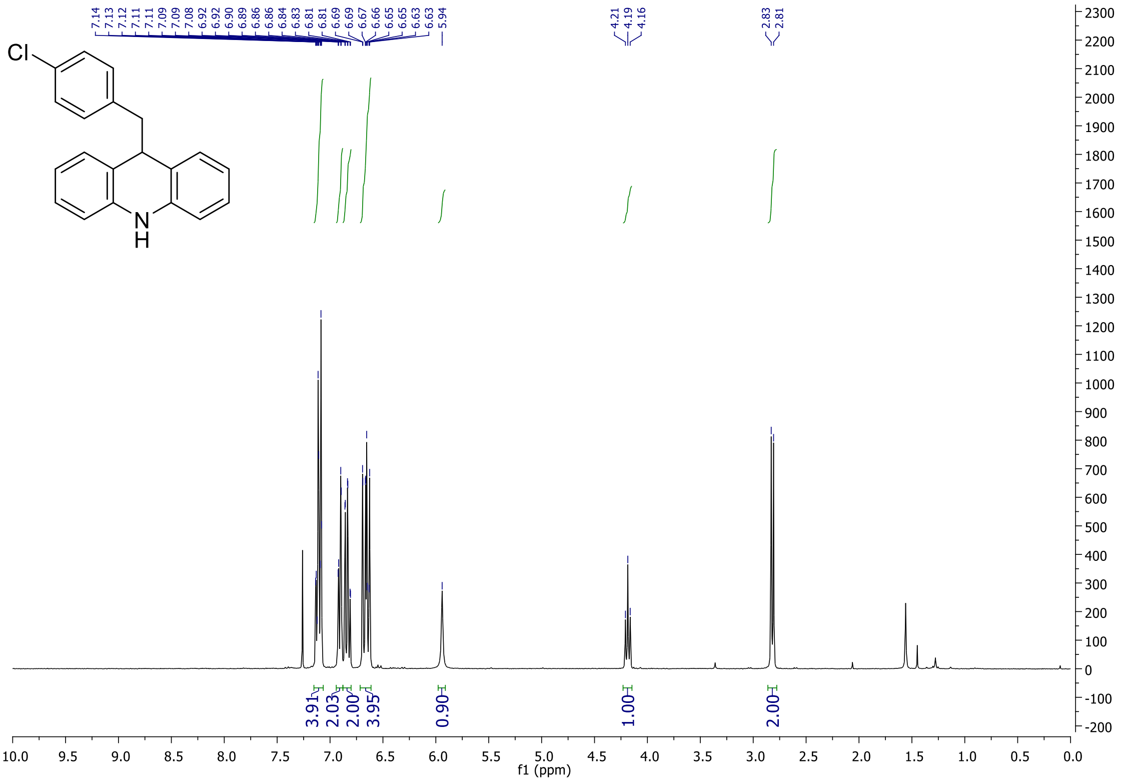

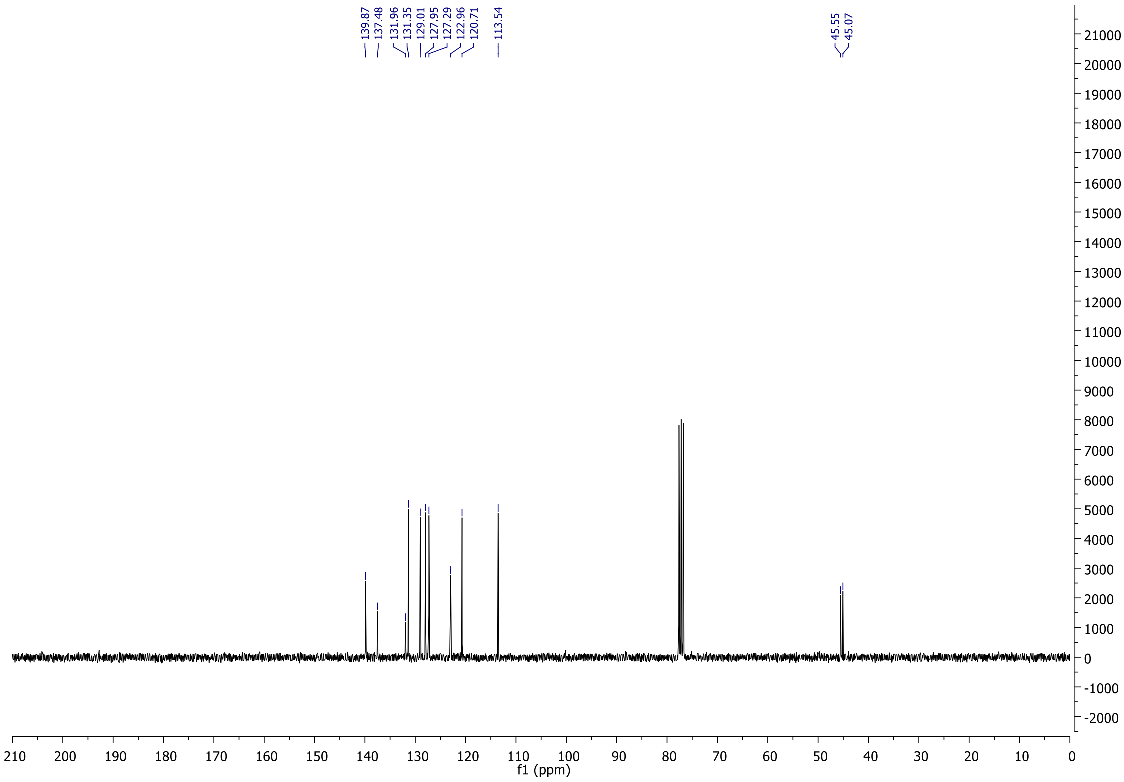

9-(2-Iodobenzyl)-9,10-dihydroacridine (4e)

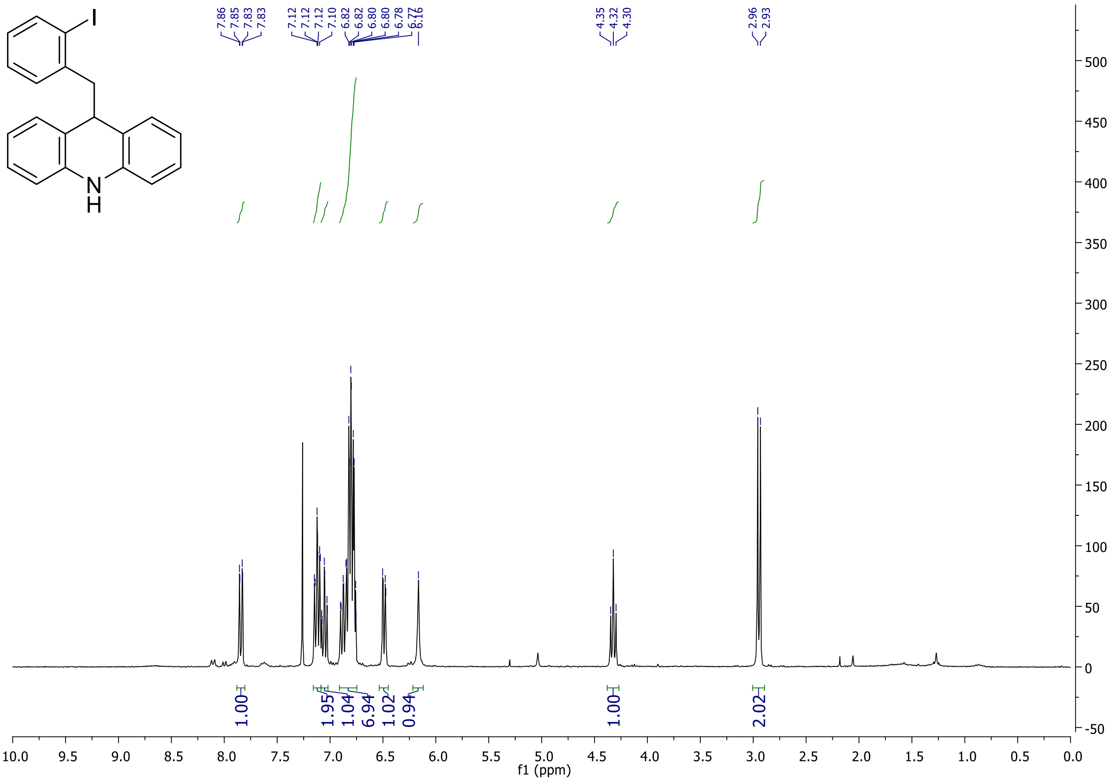

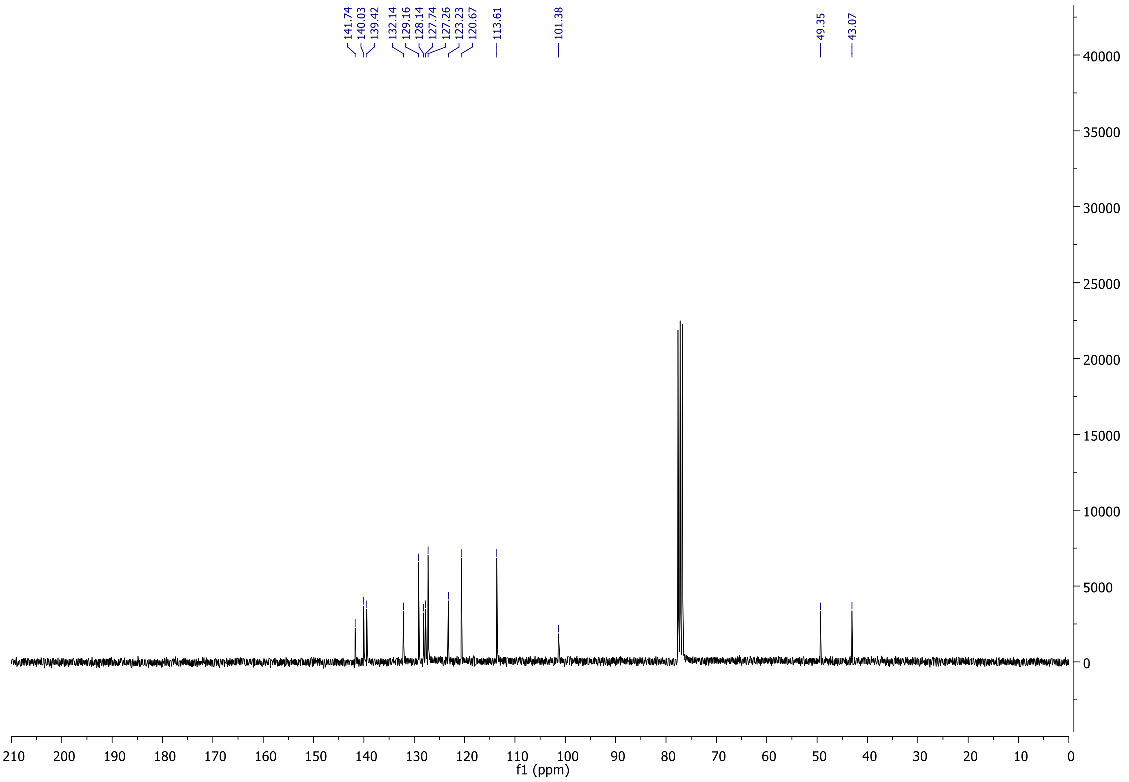

9-(4-(Trifluoromethyl)benzyl)-9,10-dihydroacridine (4f)

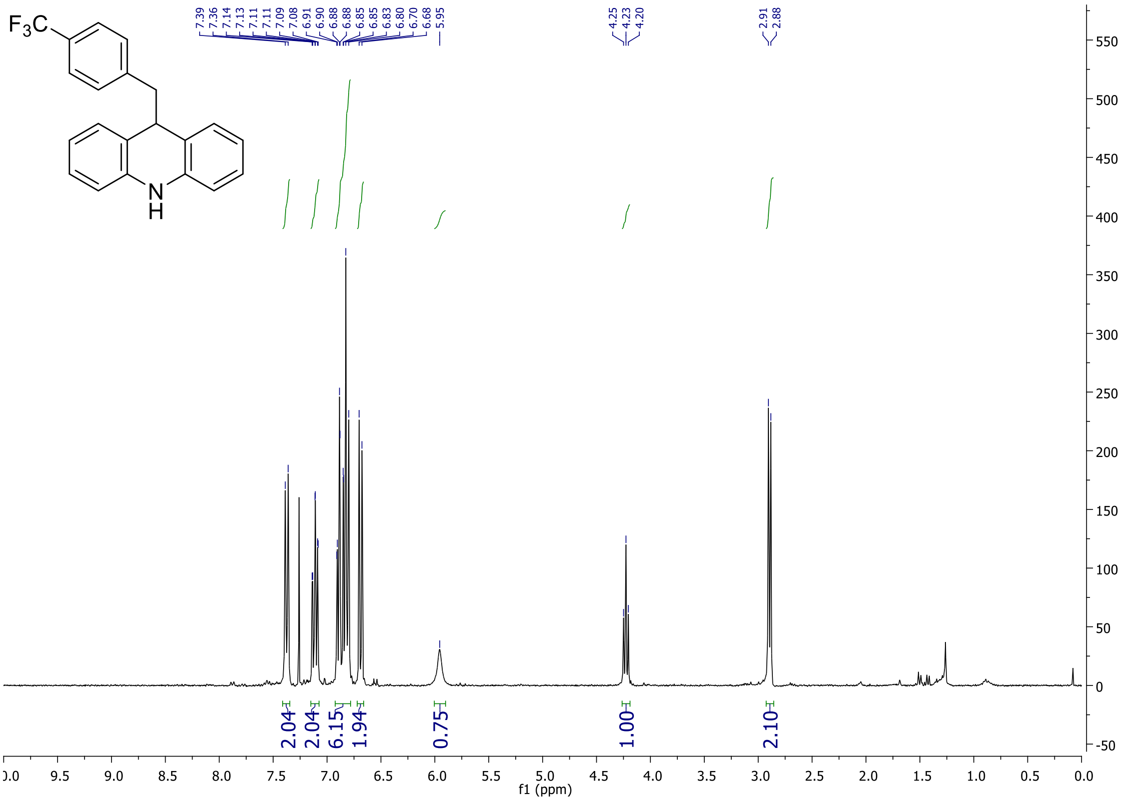

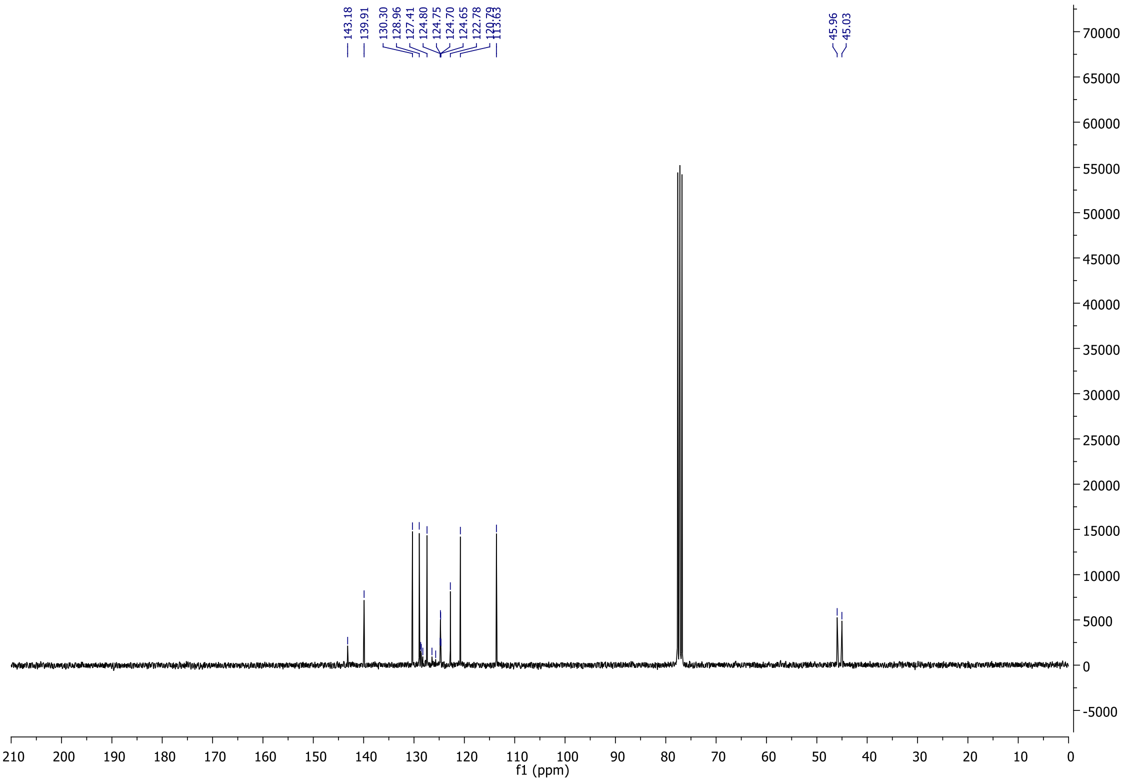

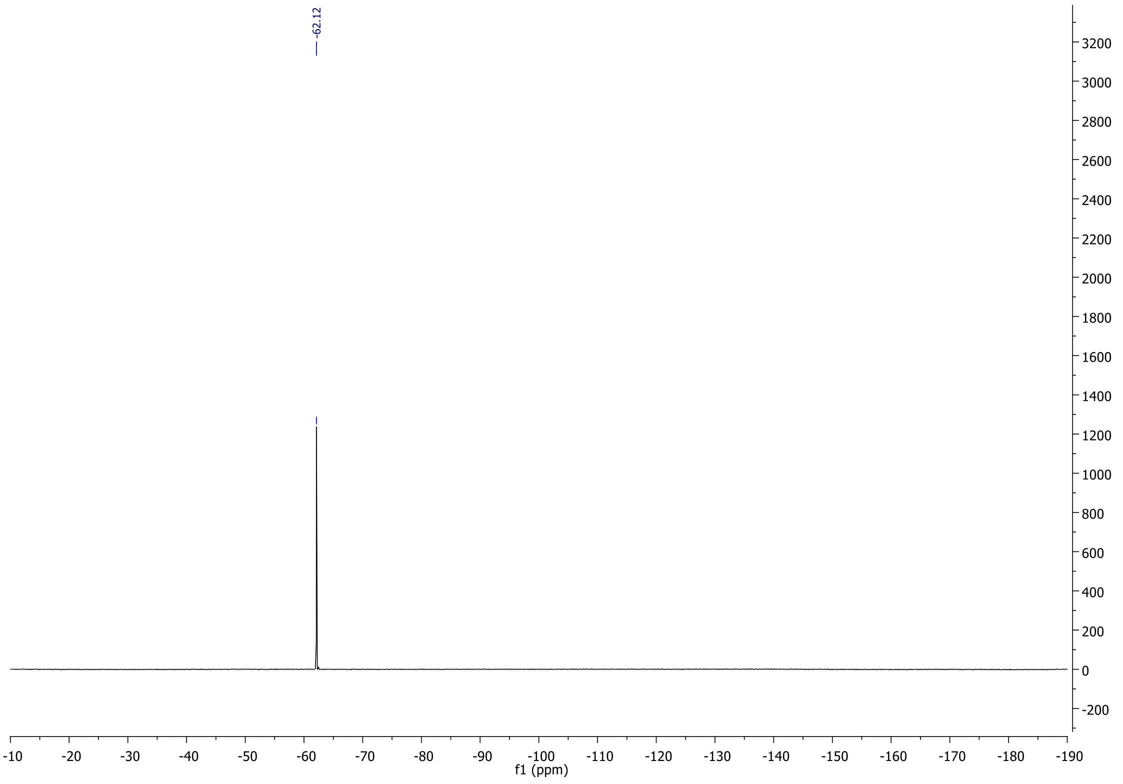

-62.12
3200
3000
2800
2600
2400
2200
2000
1800
1600
1400
1200
1000
800
600
400
200
0
-200
-10 -20 -30 -40 -50 -60 -70 -80 -90 -100 -110 -120 -130 -140 -150 -160 -170 -180 -190
f1 (ppm)

9-(3,5-Dimethoxybenzyl)-9,10-dihydroacridine (4g)

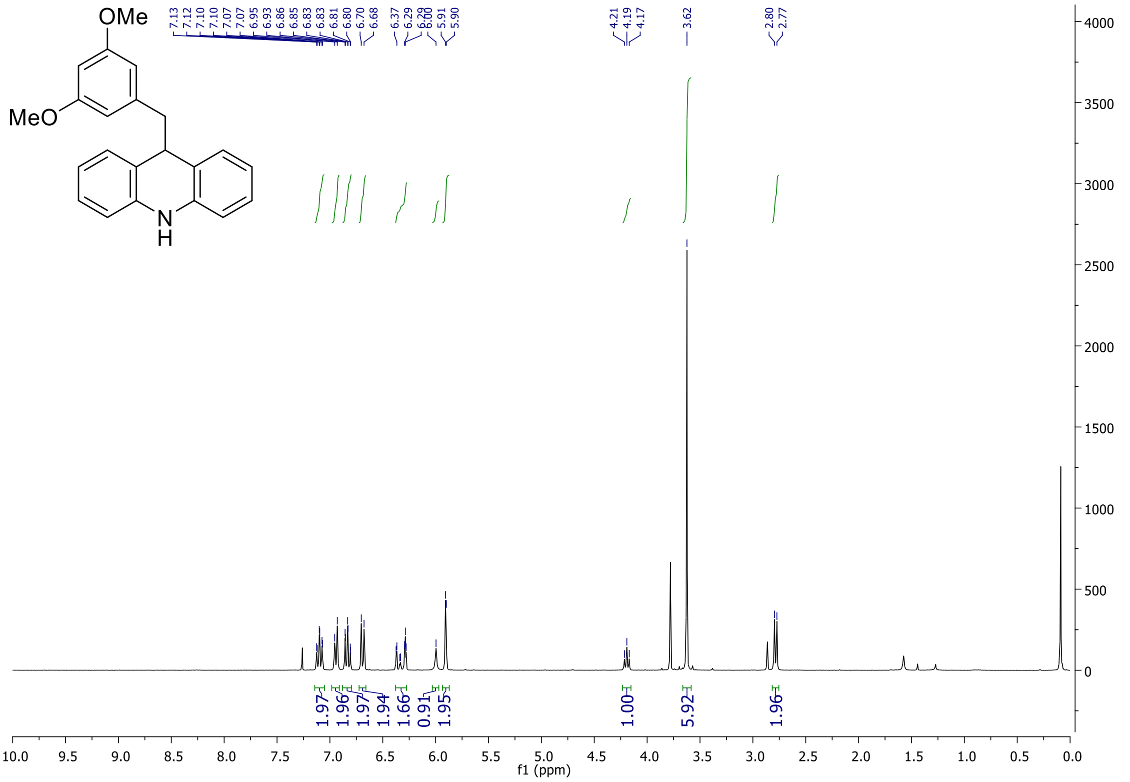

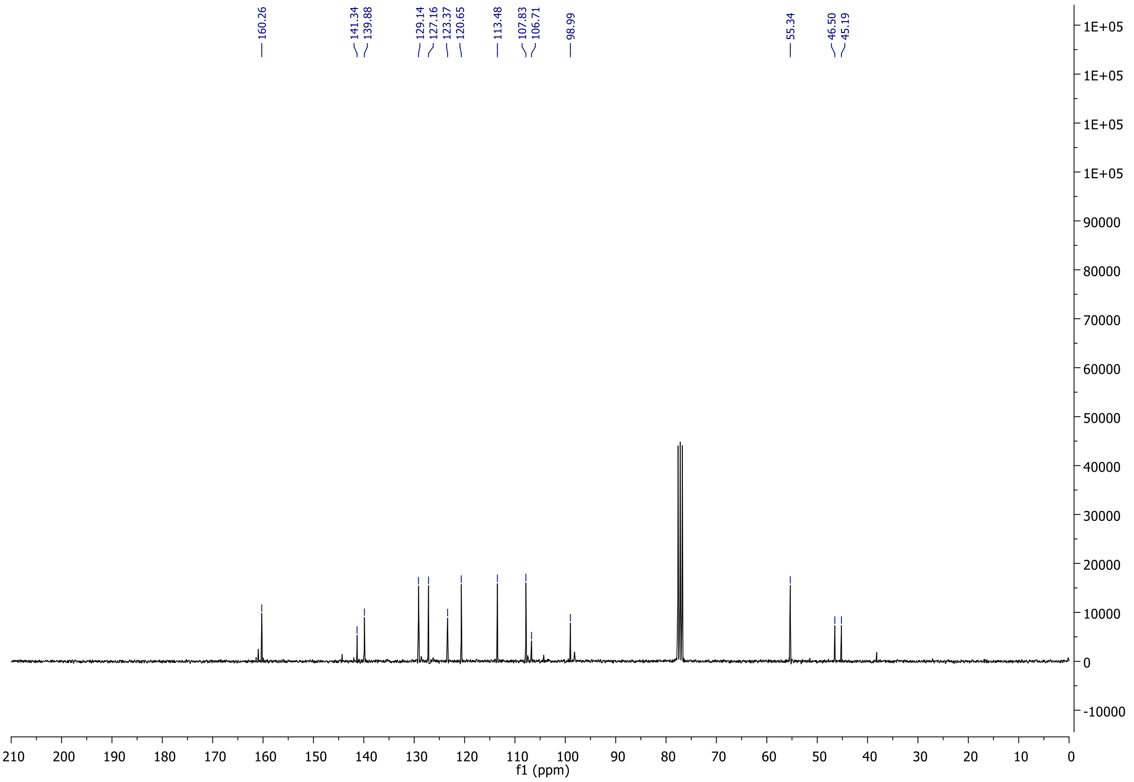

9-(Benzo[*d*][1,3]dioxol-5-ylmethyl)-9,10-dihydroacridine (4h)

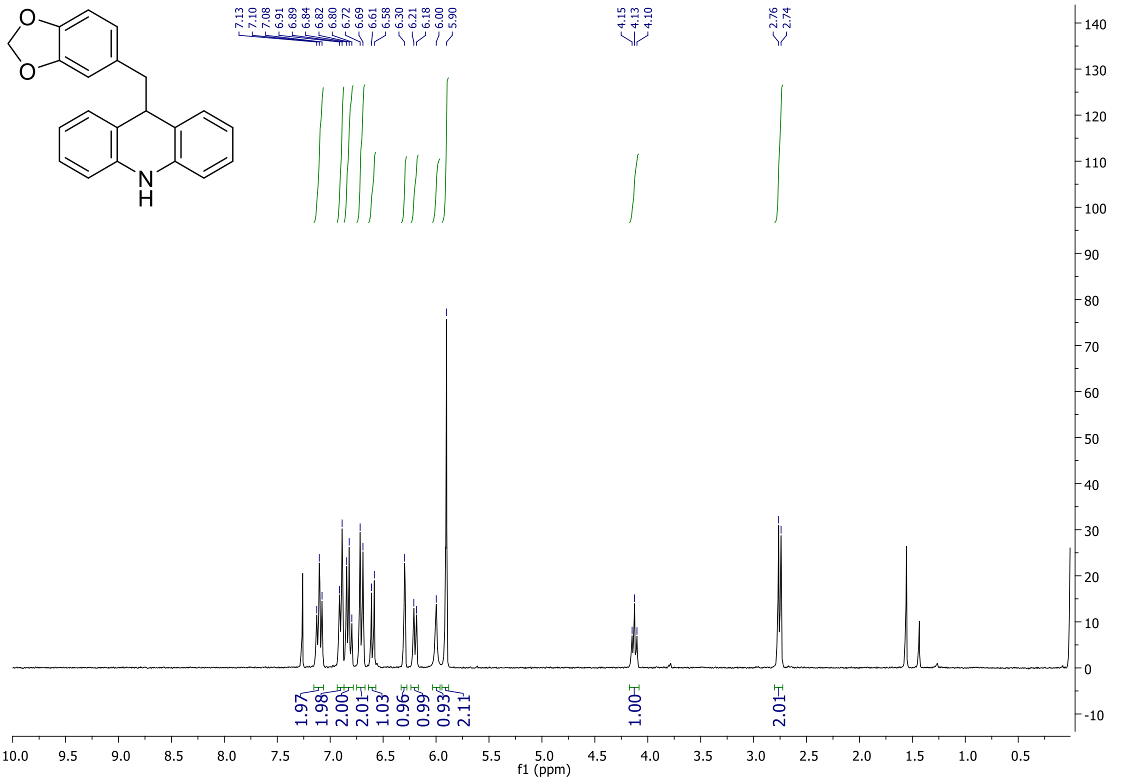

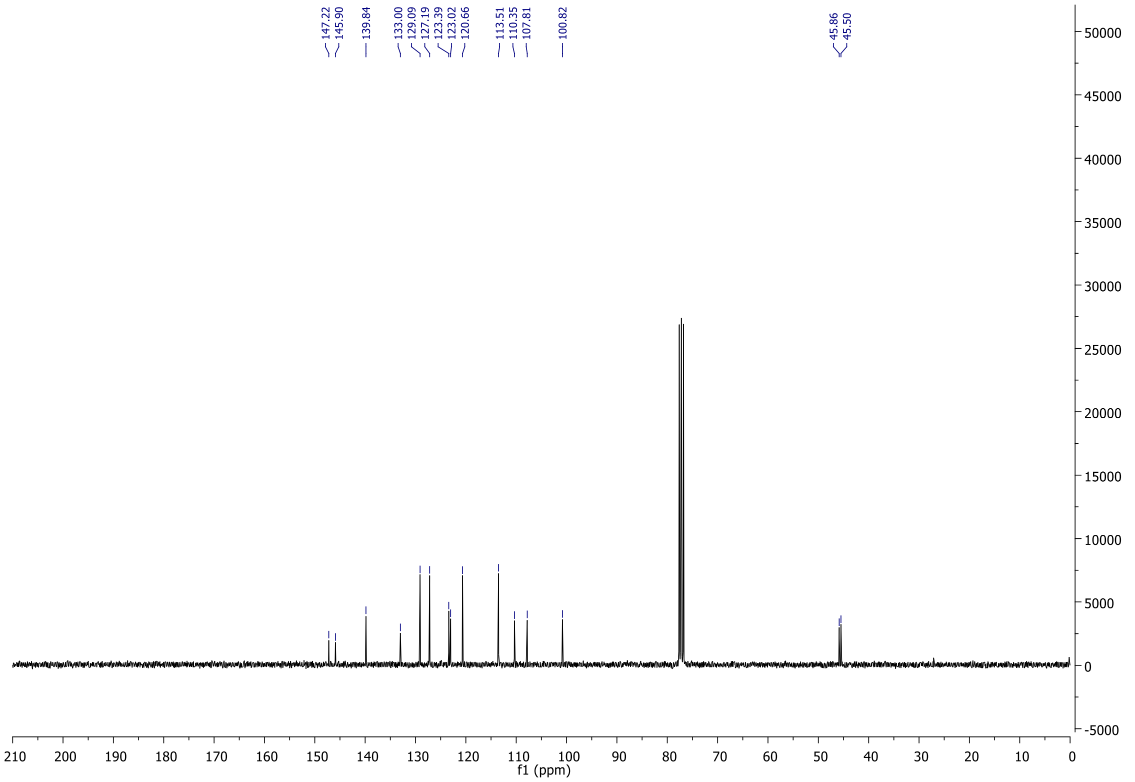

6-Bromo-2-isopropylquinoline-4-carboxylic acid (7) *NMR Solvents: $CDCl_3$ + few drops CD_3OD*

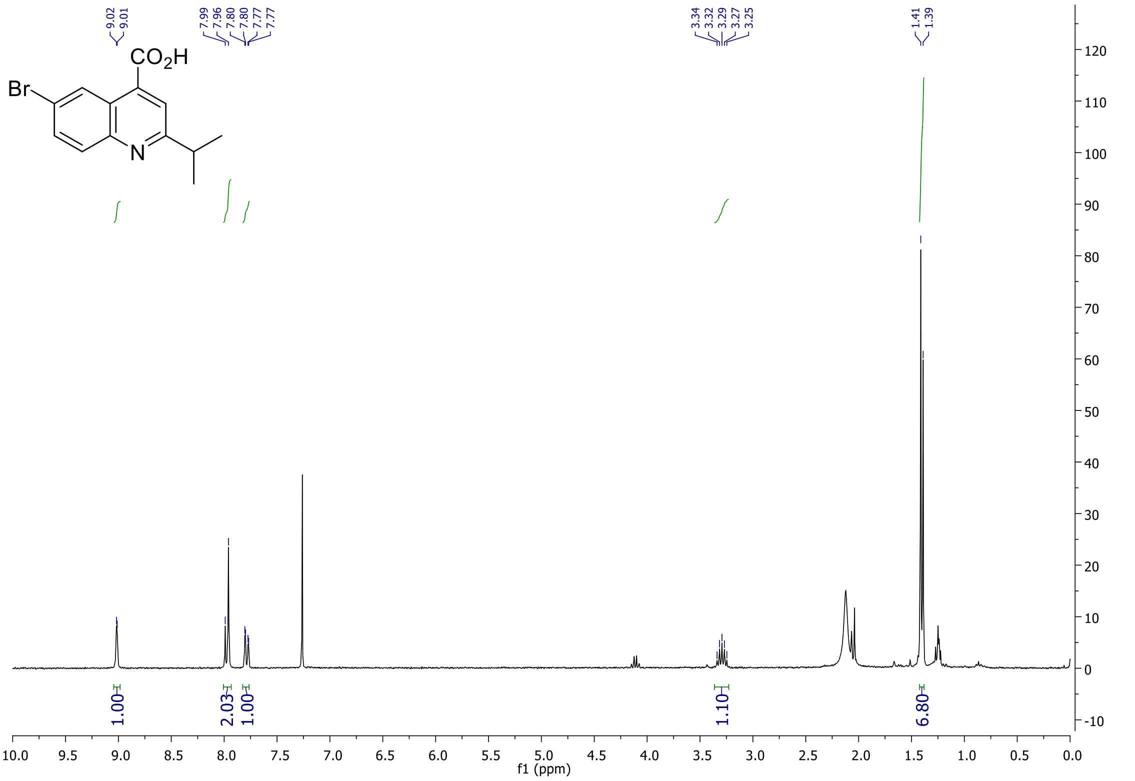

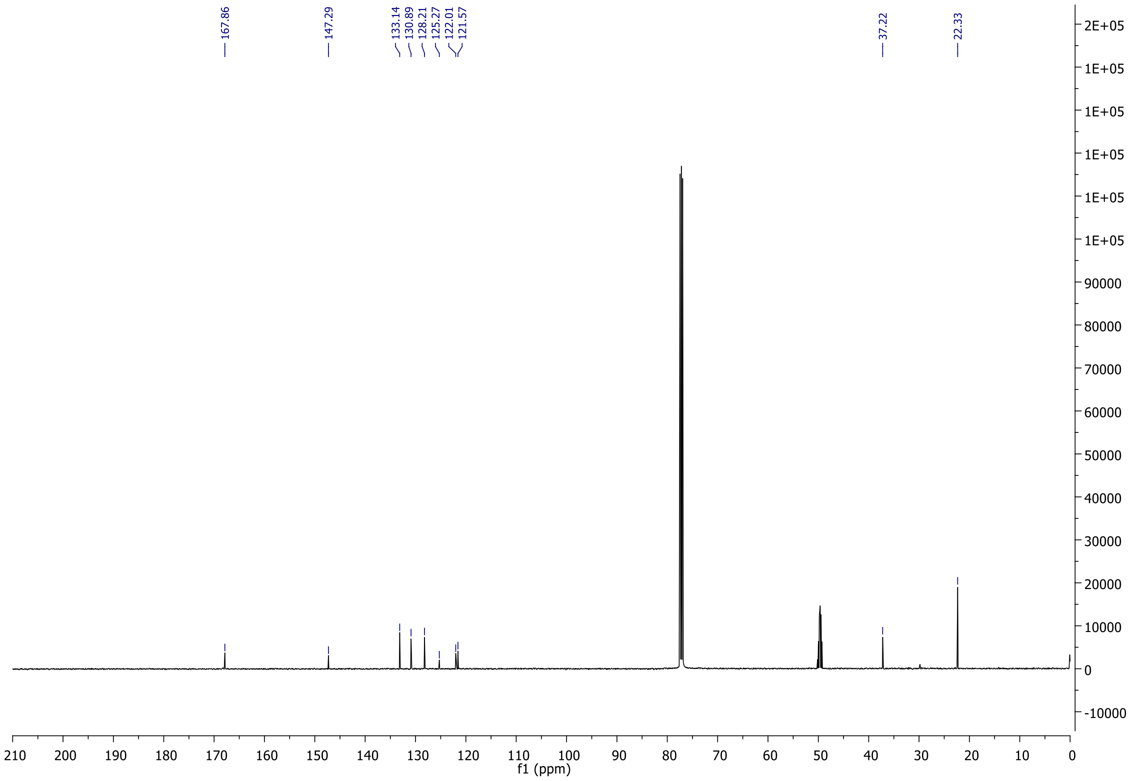

9-(Benzo[d][1,3]dioxol-5-ylmethyl)acridine (5)

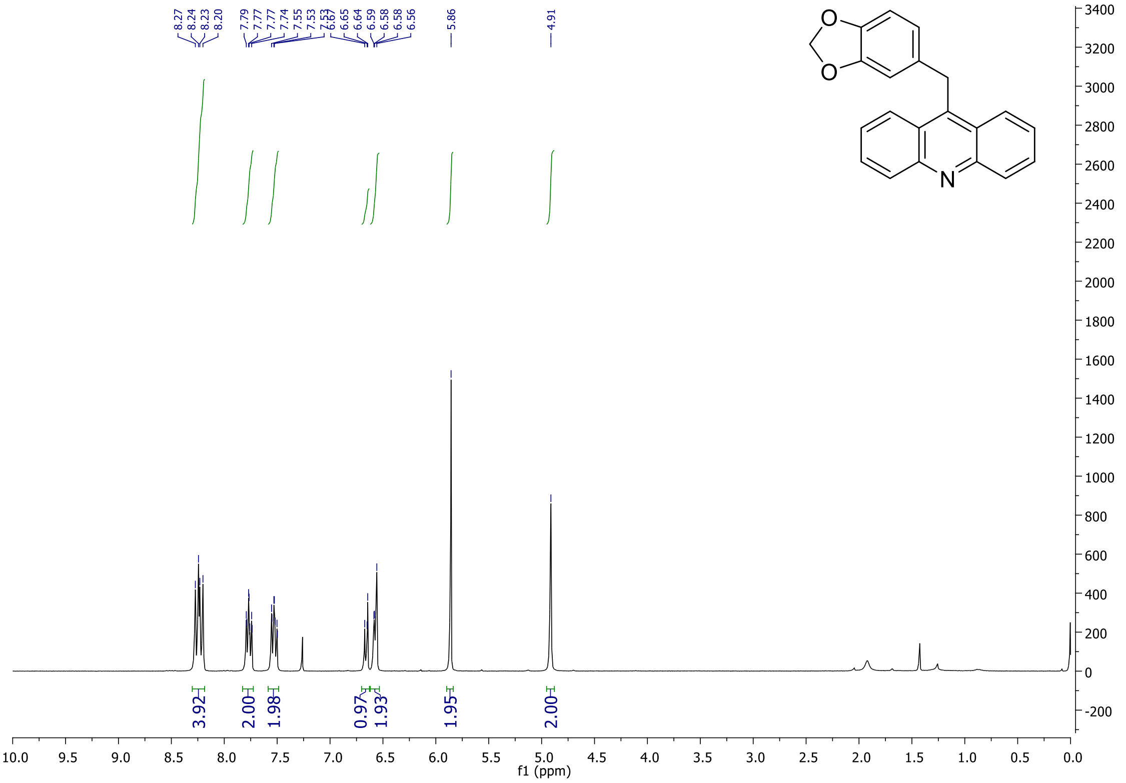

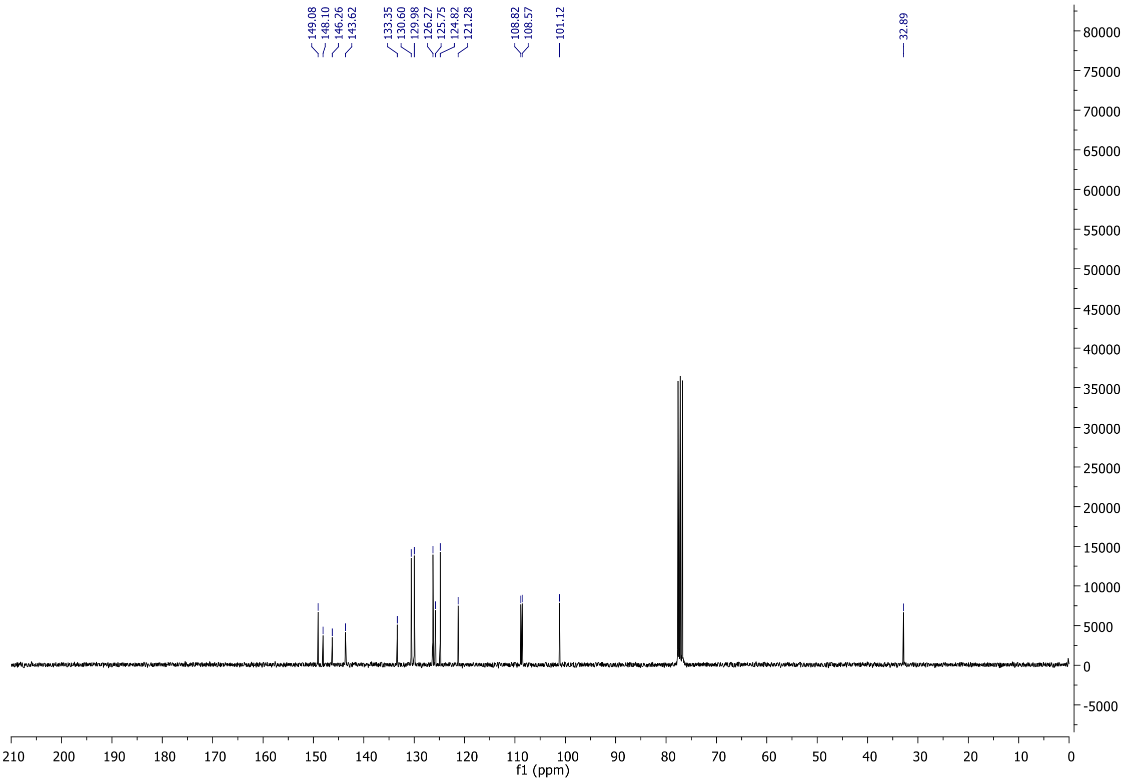

7. Cyclic Voltammetry

CVs were performed under argon atmosphere at room temperature, using 0.25 M tetrabutylammonium hexafluorophosphate ($TBAPF_6$) solution in acetonitrile (CH_3CN) as electrolyte. Measurements were carried out by using an Ivium CompaqStat potentiostat interfaced with a computer. A standard three-electrode electrochemical cell was used. Potentials were referred to an Ag/AgCl, $TBAPF_6$ 0.4 M reference electrode in ethylene glycol, and measured potentials were calibrated using an internal Fc/Fc+ standard. The working electrode used to perform the experiments was a glassy carbon electrode. The counterelectrode consisted of a Pt electrode immersed in a conductive solution.

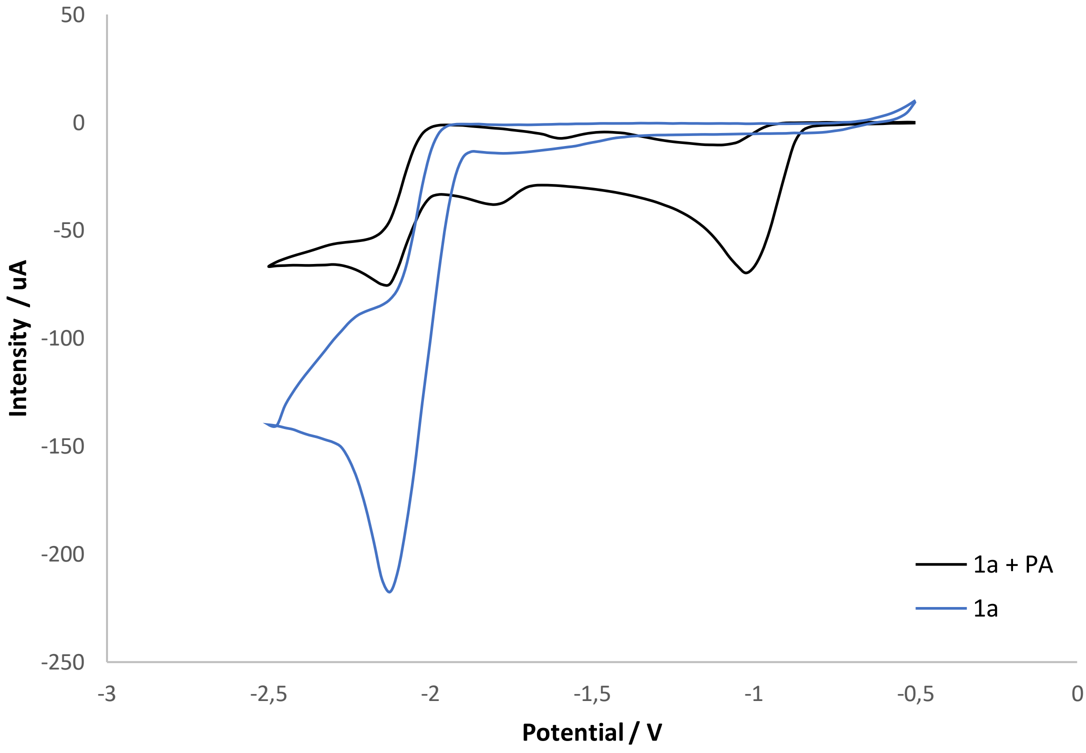

Figure S1. Cyclic voltammetry of 4-methylquinoline (**1a**) (blue) *vs* 4-methylquinoline (**1a**) activated with 1 equivalent of diphenyl phosphate (black). They were measured in CH_3CN (0.25 M $TBAPF_6$) at 100 mV/s using glassy carbon electrode as WE, Ag/AgCl as RE and Pt bar as CE.

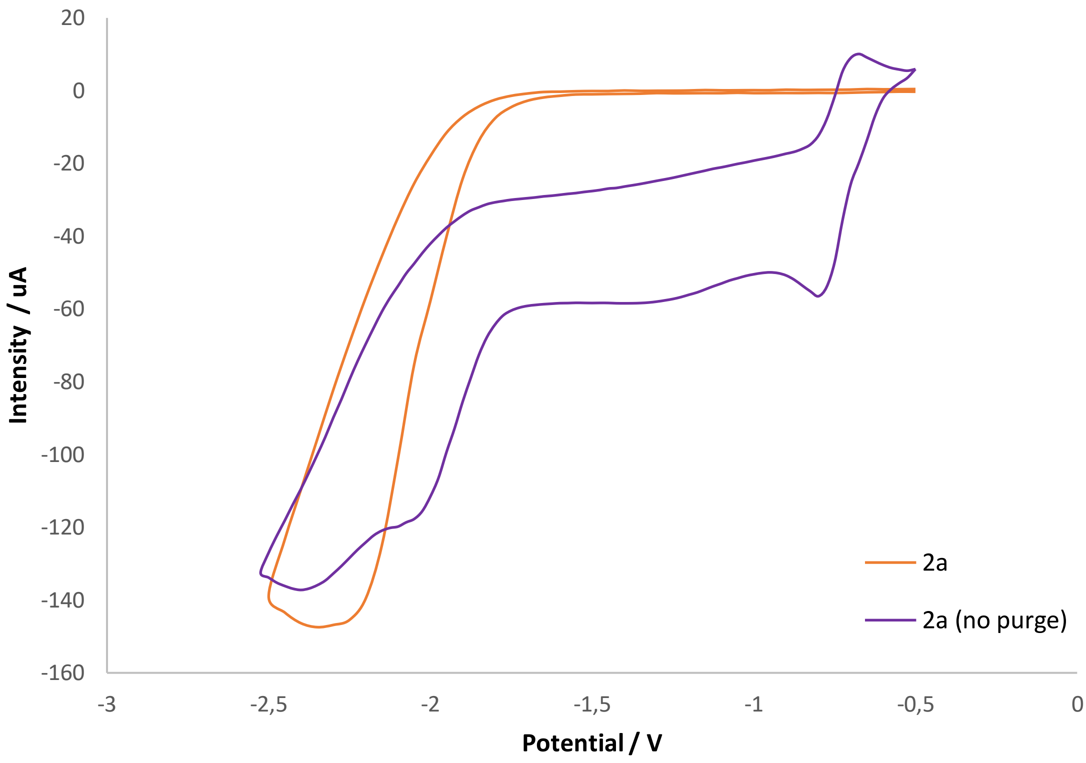

Figure S2. Cyclic voltammogram of iodocyclohexane (**2a**) under argon (orange) *vs* iodocyclohexane (**2a**) with the oxygen from the solvent (purple). They were measured in CH_3CN (0.25 M $TBAPF_6$) at 100 mV/s using glassy carbon electrode as WE, Ag/AgCl as RE and Pt bar as CE.

8. References

1. For the synthesis of starting material **1i** and **1j** see: Taylor, Alexander M., WO2018218133, **2018**. For the synthesis of starting material **10** see: Fei, H.; Xu, Z.; Wu, H.; Zhu, L.; Jalani, H. B.; Li, G.; Fu, Y.; Lu, H**.** Stereospecific Electrophilic Fluorocyclization of α,β-Unsaturated Amides with Selectfluor. *Org. Lett.*, **22** (7), 2651–2656 (2020).
2. a) Wang, Y.; Gu, M. The Concept of Spectral Accuracy for MS. *Anal. Chem.*, **82**, 7055-7062 (2010); b) Y. Wang, Methods for Operating MS Instrument Systems, United States Patent N°. 6,983,213, **2006**; c) Ochiaia, N.; Sasamoto, K.; MacNamara, K. Characterization of sulfur compounds in whisky by full evaporation dynamic headspace and selectable one-dimensional/two-dimensional retention time locked gas chromatography–mass spectrometry with simultaneous element-specific detection. *Journal of Chromatography A*, **1270**, 296-304 (2012); d) Ho, H.; Lee, R.; Chen, C.; Wang, S.; Li Z.; Lee, M. Identification of new minor metabolites of penicillin G in human serum by multiple-stage tandem mass spectrometry. *Rapid Commun Mass Spectrom.*, **25**, 25-32 (2011).
3. Li, Z.; Wang, X.; Xia, S.; Jin, J. Ligand-Accelerated Iron Photocatalysis Enabling Decarboxylative Alkylation of Heteroarenes. *Org. Lett.*, **21**, 4259-4265 (2019).
4. Presset, M.; Fleury-Brégeot, N.; Oehlrich, D.; Rombouts, F.; Molander, G.A. Synthesis and Minisci Reactions of Organotrifluoroborato Building Blocks. *J. Org. Chem.*, **78**, 4615-4619 (2013).
5. Zhang, X.-Y.; Weng, W.-Z.; Liang, H.; Yang, H.**;** Zhang, B. Visible-Light-Initiated, Photocatalyst-Free Decarboxylative Coupling of Carboxylic Acids with *N*-Heterocycles. *Org. Lett.*, **20**, 4686-4690 (2018).
6. Hu, X.; Li, G.-X.; He, G.; Chen, G. Minisci C–H alkylation of N-heteroarenes with aliphatic alcohols *via* β-scission of alkoxy radical intermediates. *Org. Chem. Front.,* **6**, 3205-3209 (2019).
7. Wang, J.; Li, G.-X.; He, G.; Chen, G. Photoredox-Mediated Minisci Alkylation of N-Heteroarenes using Carboxylic Acids and Hypervalent Iodine. *Asian J. Org. Chem.,* **7**, 1307-1310 (2018).
8. Chen, X.; Sun, W.; Huang, S.; Zhang, H.; Lin, G.; Li, H.; Qiao, J.; Li, L.; Yang, S**.** Discovery of Potent Small-Molecule SIRT6 Activators: Structure–Activity Relationship and Anti-Pancreatic Ductal Adenocarcinoma Activity. *J. Med. Chem.,* **63**, 10474-10495 (2020).
9. Rammal, F.; Gao, D.; Boujnah, S.; Gaumont, A.-C.; Hussein, A. A.; Lakhdar, Sami. Visible-Light-Mediated C−H Alkylation of Pyridine Derivatives. *Organic Letters*, **22**, 7671-7675 (2020).

10. Dong, J.; Lyu, X.; Wang, Z.; Wang, X.; Song, H.; Liu, Y.; Wang, Q. Visible-light-mediated Minisci C–H alkylation of heteroarenes with unactivated alkyl halides using O2 as an oxidant. *Chem. Sci.*, **10**, 976-982 (2019).
